# Heavy-atom functionalization promotes triplet-assisted charge-transfer exciton transport in organic cocrystals

**DOI:** 10.1038/s41467-026-72317-8

**Published:** 2026-04-22

**Authors:** Yejun Xiao, Yaxiong Wei, Min Zhang, Rui Cai, Xuan Liu, Peng Xu, Shengye Jin, Jing Leng, Wenming Tian

**Affiliations:** 1 State Key Laboratory of Chemical Reaction Dynamics, Dalian Institute of Chemical Physics, Chinese Academy of Sciences, Dalian, China; 2 Anhui Province Key Laboratory for Control and Applications of Optoelectronic Information Materials, School of Physics and Electronic Information, Anhui Normal University, Wuhu, China; 3 University of Chinese Academy of Sciences, Beijing, China; 4 Instrumental Analysis Center, Dalian University of Technology, Dalian, China; 5 State Key Laboratory of Photoelectric Conversion and Utilization of Solar Energy, Dalian National Laboratory for Clean Energy, Dalian Institute of Chemical Physics, Chinese Academy of Sciences, Dalian, China

**Keywords:** Energy transfer, Fluorescence spectroscopy, Organic molecules in materials science

## Abstract

Charge-transfer (CT) states with long transport distance are highly desirable for boosting the performance of organic optoelectronic devices. Although micron-scale CT transport has been observed in cocrystals, effective strategies for enhancing the diffusivity of CT excitons remain a challenge. Herein, based on heavy atom effect (HAE), we successfully promote CT exciton transport in thermally activated delayed fluorescence (TADF) cocrystals through bromine-atom functionalization. In Br-functionalized cocrystal, the diffusivity of triplet CT excitons is enhanced by an order of magnitude, enabling a long-distance triplet-assisted CT transport exceeding 16 μm. By adjusting the Br content, the CT transport and TADF-related kinetics can be effectively modulated, thereby significantly enhancing the utilization of CT excitons and the photocurrent responses of cocrystals. Our findings provide compelling evidence that heavy-atom functionalization can serve as an effective strategy to promote CT transport, which is of great significance for the performance optimization of organic optoelectronic devices.

## Abstract (web summary)

Charge-transfer states with long transport distance have potential in organic optoelectronic devices, but it can be challenging to improve this. Here, the authors report the promotion of exciton transport in thermally activated delayed fluorescence cocrystals by bromine-atom functionalisation.

## Introduction

Organic optoelectronic devices have attracted significant attention due to their potential to revolutionize technologies ranging from displays to solar energy conversion, owing to their lightweight, flexible, and cost-effective nature^1–6^. In such devices, charge-transfer (CT) excitons, formed at the interface between donor (D) and acceptor (A) molecules, act as key intermediates that govern charge separation and recombination, thereby significantly influencing the device performance^1,7–10^. Moreover, benefiting from the spatial separation of electron-hole pairs in the CT state, the energy difference $\left(\varDelta {E}_{{ST}}\right)$ between their singlet and triplet states (^1^CT-^3^CT) is typically small^5,10,11^. This small gap facilitates efficient thermally activated delayed fluorescence (TADF) emission through the efficient reverse intersystem crossing (RISC), thus holding great promise for enhancing exciton utilization^5,11,12^.

**Fig. 1:**
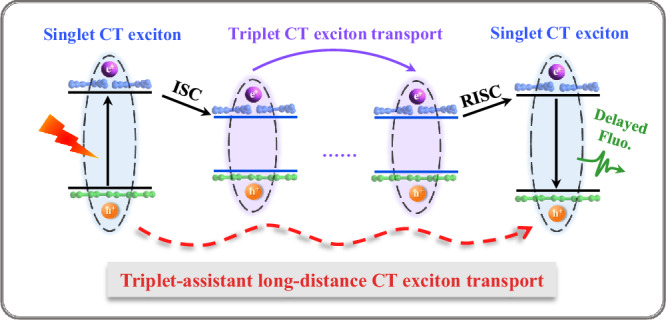
Schematic of triplet-assisted micron-scale CT exciton transport. Through intersystem crossing (ISC), singlet CT excitons can be rapidly converted into long-lived, diffusive triplet states for long-distance transport, followed by efficient reverse intersystem crossing (RISC) back to singlets. This enables an equivalent long-distance singlet CT exciton transport assisted by the triplet state.

The transport of CT excitons is a critical kinetic parameter that fundamentally governs the efficiency of functional processes in organic optoelectronics, such as photovoltaic conversion and electroluminescence^13–17^. Although highly mobile CT excitons have been observed in many inorganic-inorganic and organic-inorganic heterostructures (e.g., 2D WSe_2_-WS_1.16_Se_0.84_ and WS_2_-tetracene heterojunctions)^17,18^, their diffusion lengths in pure organic systems are often limited to within ~tens of nanometers due to intrinsically low diffusivity and short lifetimes^15,16^. Intriguingly, our recent study on an organic cocrystal has revealed that the intrinsic nanoscale transport of singlet CT state can be significantly extended to the micrometer scale through a triplet-assisted mechanism (Fig. 1), where singlet excitons can be quickly “stored” into long-lived triplet states through intersystem crossing (ISC), enabling long-distance transport before returning to the singlet via efficient RISC^19^. This route is broadly applicable to materials that combine efficient TADF with long-lived and diffusive triplet states, thereby providing a promising approach to enhance CT transport. However, the diffusivity of currently observed CT excitons remains low ( ~ 10^-4 ^cm^2^/s), and the RISC process in these organic cocrystals is also inefficient due to the weak spin-orbit coupling (SOC)^20–22^. These limitations substantially diminish the actual utilization of CT excitons despite their potential for long-distance migration. Therefore, developing effective strategies to simultaneously promote CT transport and RISC process is highly desirable for advancing organic optoelectronics.

Heavy atom effect (HAE) is a well-established strategy for device optimization, as it significantly enhances the SOC interaction in organic materials^23–26^. Heavy atoms (e.g., Br, I), which possess larger relativistic mass and electron density than light atoms (e.g., C, H), typically exhibit stronger atomic SOC^24,26^. The enhanced SOC effectively promotes both ISC and RISC processes, thereby enhancing the efficiency of TADF emission^24,27,28^. Moreover, some theoretical studies have indicated that incorporating heavy atoms may facilitate the transport of charged carriers by increasing intermolecular orbital overlap^29,30^. These findings suggest that HAE could potentially improve not only TADF-related kinetics but also charge transport properties. However, whether such enhancements extend to CT exciton transport remains unclear. To date, the influence of HAE on CT exciton transport has not been systematically investigated, and direct compelling experimental evidences are still lacking.

In this work, organic TADF cocrystals with well-defined crystalline structures and intense TADF emissions were employed as model systems for the exploration of CT exciton transport dynamics^31–34^. Two representative TADF cocrystals were synthesized: one with bromine functionalization (*trans*-4-bromostilbene-1,2,4,5-tetracyanobenzene, named as T_Br_-T_C_) and one without (*trans*−1,2-diphenylethylene-1,2,4,5-tetracyanobenzene, named as T_S_-T_C_). By using TADF-scanned imaging microscopy, the influence of HAE on the long-distance CT exciton transport was systematically investigated. Compared to T_S_-T_C_, incorporating Br groups in T_Br_-T_C_ not only significantly promotes its ISC and RISC kinetics, but also results in a 10-times enhancement of triplet CT exciton diffusivity and an average CT migration distance exceeding 16 μm. Moreover, we also found that by adjusting Br content in these cocrystals, CT transport dynamics can be effectively modulated, which plays a critical role in determining their photocurrent responses. These results establish HAE as an effective handle to synchronously boost RISC and long-distance CT transport in organic cocrystals.

## Results and discussion

### Crystal structures of T_S_-T_C_ and T_Br_-T_C_

**Fig. 2:**
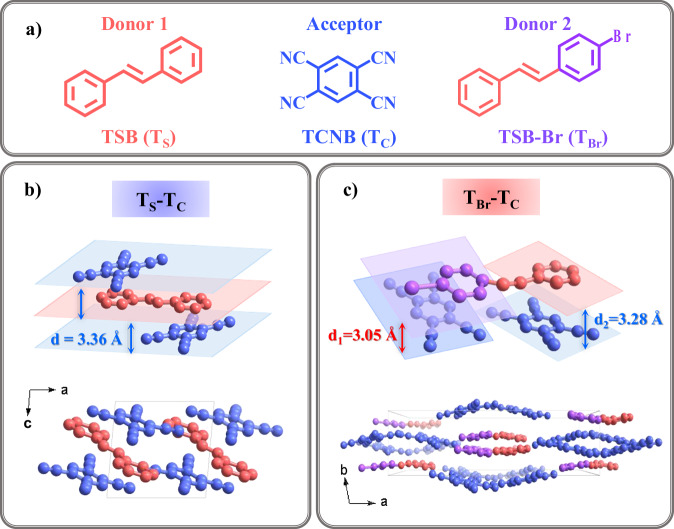
Structures of organic precursors and cocrystals. **a** Chemical structures of donors (T_S_ and T_Br_) and acceptor (T_C_) for the synthesis of T_S_-T_C_ and T_Br_-T_C_ cocrystals. The A-D-A unit structures and single crystal structures of **b** T_S_-T_C_ and **c** T_Br_-T_C_.

T_S_-T_C_ and T_Br_-T_C_ cocrystals with 1:2 D/A stoichiometry were synthesized through a simple solution drop-casting method^35,36^. In T_S_-T_C_, each benzene ring of TSB (*trans*−1,2-diphenylethylene, T_S_) donor is coupled with a TCNB (1,2,4,5-tetracyanobenzene, T_C_) acceptor (Fig. 2a), forming a symmetric A-D-A spatial structure with D-A intermolecular distance of ~3.36 Å (Fig. 2b). The growth of T_S_-T_C_ cocrystal further leads to its crystallization in a triclinic system with *P-1* space group (Fig. 2b and Supplementary Fig. 1). The cocrystal synthesis using Br-functionalized TSB (*trans*-4-bromostilbene, T_Br_, see Fig. 2a) as the alternative donor results in significant structural changes, as evidenced by the refined data from single-crystal X-ray diffraction (SXRD) analyses (Supplementary Table 1). The asymmetrical molecular structure of T_Br_ breaks the spatial symmetry of the A-D-A unit, and leads to the crystallization of T_Br_-T_C_ in a monoclinic system with *Cc* space group (Fig. 2c and Supplementary Fig. 2). The high consistency between the experimental and simulated powder XRD patterns of T_Br_-T_C_ indicates a good phase purity of the synthesized T_Br_-T_C_ cocrystal (Supplementary Fig. 3). The D-A distances in T_Br_-T_C_ were measured to be ~3.05 Å and ~3.28 Å for segments with and without Br group, respectively (Fig. 2c). Both distances slightly decrease compared to ~3.36 Å observed in T_S_-T_C_, indicating a HAE-induced CT coupling enhancement in T_Br_-T_C_^24^.

### Dynamic mechanism of CT state emission in T_S_-T_C_ and T_Br_-T_C_

**Fig. 3:**
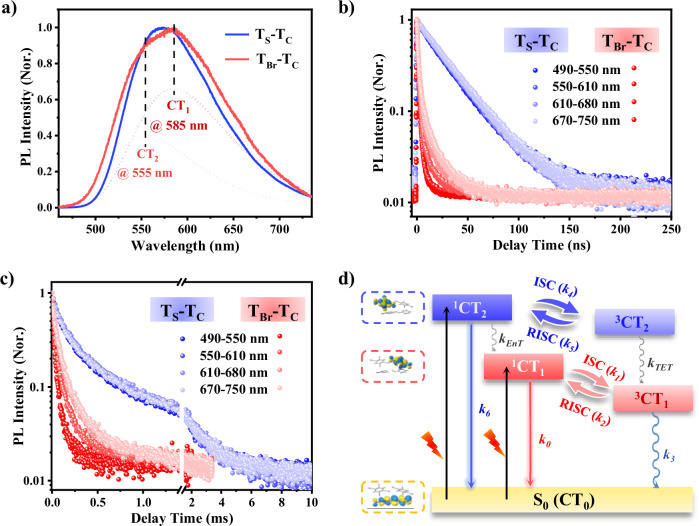
Carrier dynamics in T_S_-T_C_ and T_Br_-T_C_. **a** PL spectra of T_S_-T_C_ and T_Br_-T_C_ under the 375 nm excitation. The dashed lines are the PL spectra of CT_1_ (dark red) and CT_2_ (light red) state emissions after multi-peak fitting, which are centered at ~585 nm and ~555 nm, respectively. PL kinetics collected on **b** nanosecond and **c** millisecond timescales based on normal TCSPC technique and multipulse-excited TCSPC phosphorescence decay recording technique, respectively. **d** Schematic diagram for dual TADF emission mechanism in T_Br_-T_C_.

Photoluminescence (PL) measurements were initially conducted to investigate the characteristics of the CT states. The PL spectrum of T_S_-T_C_ is dominated by single emission peak at ~574 nm (Fig. 3a), while T_Br_-T_C_ exhibits clear double-peak emission feature. The PL kinetics of T_S_-T_C_ collected within nano- and milli-second time windows both exhibit similar decay profiles at different wavelength ranges (Fig. 3b, c), consistent with its CT-state features as previously reported^19,37^, indicating the single-component emission from CT state in T_S_-T_C_. Notably, the delayed PL kinetics in Fig. 3c have been corrected to eliminate interference from the initial fluorescence (see Supplementary Fig. 4 for details). After Br functionalization, both the prompt and delayed PL kinetics are accelerated and exhibit clear wavelength-dependent behaviors on both ns and ms timescales (Fig. 3b, c). The delayed PL spectra of T_Br_-T_C_, captured by the ICMOS camera, also show a continuous redshift in emission peak centered from ~555 nm to ~585 nm (Supplementary Fig. 5). These observations are consistent with the steady-state double-peak emission of T_Br_-T_C_ (Fig. 3a), potentially indicating the coexistence of dual luminescent components in T_Br_-T_C_.

To further unveil the influence of HAE on CT dynamics, theoretical calculations of energy levels in T_Br_-T_C_ were performed (Supplementary Table 2). The calculation results reveal that there are two energetically distinct CT states in T_Br_-T_C_, localized on subunits with and without Br group, respectively (denoted as CT_2_ and CT_1_ state, Supplementary Fig. 6). The energy level of CT_2_ state with Br functionalization is ~90 meV higher than that of CT_1_ state, in good agreement with the energy difference of ~114 meV derived from the double-peak positions in PL spectra of T_Br_-T_C_ (Fig. 3a). Therefore, it can be speculated that the dual-peak emission in T_Br_-T_C_ arises from electronic transitions of both CT_1_ and CT_2_ states. The SOC matrix elements between the singlet and triplet CT states in T_Br_-T_C_ and T_S_-T_C_ were also calculated (Supplementary Table 3). For T_Br_-T_C_, the values of <S_1_ | H_SO_ | T_1_> and <S_2_ | H_SO_ | T_2_> were 0.011 and 0.103 cm^-1^, respectively, both exceeding the corresponding value of <S_1_ | H_SO_ | T_1_> in T_S_-T_C_ (0.007 cm^-1^), directly verifying the HAE-induced SOC enhancement in T_Br_-T_C_. The enhanced SOC intensities lead to the accelerated PL kinetics for both CT_1_ and CT_2_ states in T_Br_-T_C_. Moreover, the distinct kinetic features of these two CT states, along with a potential CT_2_-to-CT_1_ energy transfer, contribute to the wavelength-dependent kinetic behavior observed in T_Br_-T_C_ (Fig. 3b, c). It is noteworthy that, despite having similar D-A subunits, the CT_1_ emission in T_Br_-T_C_ ( ~585 nm) is slightly redshifted relative to that in T_S_-T_C_ ( ~574 nm). This may originate from the shortened D-A distances in T_Br_-T_C_ (Fig. 2b, c), which enhances the CT coupling and results in a narrower band gap of its CT_1_ state ^38,39^.

Beyond clarifying the dual emission mechanism, we also aim to identify the origin of long-lived emissions in T_Br_-T_C_. On the one hand, the ${\triangle E}_{{ST}}$ values between the singlet and triplet states of each CT state in T_Br_-T_C_ are sufficiently small ( ≤ 10 meV, Supplementary Fig. 6), enabling the dual TADF emissions from both CT states, similar to the long-lived TADF emission observed in T_S_-T_C_^19,36^. On the other hand, the enhanced SOC intensity in T_Br_-T_C_ may facilitate the otherwise forbidden transition from the triplet CT state (^3^CT) to its ground state (CT₀), thereby increasing the likelihood of phosphorescence. To gain further insights, temperature-dependent PL measurements were performed on T_Br_-T_C_, with wavelength ranges of 490-550 nm and 670-750 nm intentionally selected for distinguishing CT_2_ and CT_1_ states, respectively. The delayed PL kinetics of CT_2_ state exhibit almost identical profiles at different temperatures (Supplementary Fig. 7a), while for the CT_1_ excitons, a significant increase in PL lifetime can be observed at temperature below 213 K (Supplementary Fig. 7b). The temperature insensitivity of ms-scale PL kinetics within the initial cooling range ( ≥ 213 K) aligns with the TADF behavior previously observed in T_S_-T_C_, suggesting the long-lived TADF emission from both CT states in T_Br_-T_C_ at temperature above 213 K. A similar temperature threshold can be identified from the temperature-dependent PL spectra of T_Br_-T_C_ (Supplementary Fig. 8), which exhibit a notable enhancement below 213 K, corresponding to a dominant phosphorescence emission at lower temperatures. The nearly unchanged PL intensity from room temperature (RT) to 213 K may be attributed to the counteracting effects between reduced TADF and increased fluorescence with temperature decreasing (Supplementary Fig. 8 and 9). Therefore, we speculate that the two long-lived components in T_Br_-T_C_ at RT originate from TADF emissions of CT_1_ and CT_2_ singlets, which transition into phosphorescent emissions from their triplets below 213 K. These results coincide with recent observations on 4,6-di(thianthren-1-yl)pyrimidine (PYH) and 2-chloro-4,6-di(thianthren-1-yl)pyrimidine (PYC)^40^, showing the temperature-dependent emission transition from TADF at RT to phosphorescence at low temperatures. The proposed dual TADF emission mechanism in T_Br_-T_C_ is summarized in Fig. 3d.

### Visualization of CT exciton transport facilitated by HAE

**Fig. 4:**
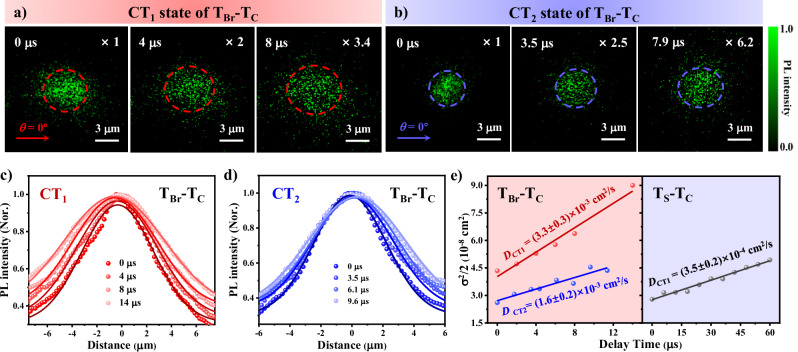
Imaging and modeling of CT exciton transport. Time-dependent TADF intensity images of **a** CT_1_ and **b** CT_2_ excitons in T_Br_-T_C_ collected at 670-750 nm and 490-550 nm, respectively. Scale bars are 3 μm. The dash lines represent the exciton distribution obtained from the Gaussian fittings. Normalized 1D TADF intensity profiles of **c** CT_1_ and **d** CT_2_ excitons in T_Br_-T_C_ extracted from the *θ*= 0° direction in panels **a** and **b**, respectively, at the indicated delay times. The solid lines are their Gaussian fittings. **e** The determination of TADF-related exciton diffusion coefficient by the linear fitting of 1D time-dependent Gaussian variances (${\sigma }^{2}\left(t\right)$) along the *θ* = 0° direction.

We next proceed to investigate the influence of HAE on CT exciton transport. Our previous study demonstrates that although the singlet CT state typically exhibits a limited transport distance of tens of nanometers, micron-scale transport can be achieved through a triplet-assisted mechanism in the presence of a long-lived triplet state and efficient TADF (Fig. 1)^19^. We therefore focus on this long-distance CT transport by employing TADF imaging on a ms timescale (see Methods for details)^19,41^. The diffusion of CT_1_ and CT_2_ excitons in T_Br_-T_C_ was primarily investigated by analyzing their TADF intensity images at various delay times (Fig. 4a, b). As the PL intensity is proportional to the exciton density, the spatial expansion of PL distribution over time directly reflects the exciton diffusion away from the initial excitation site (see Supplementary Note 1 for detailed discussion)^41,42^. To avoid the interference from initial fluorescence, the time for the second data point of TADF imaging (t = 4 μs for CT_1_ state and 1.75 μs for CT_2_ state) was reset to zero, thereby resulting in a broader initial TADF distribution (with radius of ~3.5 μm and ~3.1 μm in Fig. 4a, b, respectively) compared to the laser excitation spot ( ~ 565 nm in radius, Supplementary Fig. 10). With delay time increasing, the TADF spot of both CT states expands into a discernible elliptic pattern, indicating the anisotropic CT migration in T_Br_-T_C_ (Fig. 4a, b). Notably, the direction of maximum migration efficiency was defined as *θ* = 0°, as illustrated in Figs. 4a and 4b. For quantitative evaluation, time-dependent 1D TADF intensity profiles of both CT states were extracted along the representative directions of *θ* = 0° (Fig. 4c, d) and 90° (Supplementary Fig. S11a, b), which are well described by Gaussian functions. The diffusion coefficient (*D*) of CT_1_ and CT_2_ excitons in T_Br_-T_C_ along the *θ* = 0° direction, obtained from linear fitting of time-dependent Gaussian variances, is determined to be (3.3 ± 0.3)×10^-3 ^cm^2^/s and (1.6 ± 0.2)×10^-3 ^cm^2^/s (Fig. 4e), respectively. Both *D* values are approximately twice those along the *θ* = 90° direction (Supplementary Fig. 11c–e). The anisotropic migration of CT excitons can be readily understood by the structural asymmetry of T_Br_-T_C_ (Supplementary Fig. 2c)^42–44^, while the smaller CT_2_ diffusivity relative to the CT_1_ state is attributed to its larger effective exciton mass caused by Br incorporation^45,46^.

For comparison, the CT_1_ exciton diffusion in T_S_-T_C_ was also investigated. An isotropic exciton transport is observed, with the *D* value determined to be (3.5 ± 0.2)×10^-4 ^cm^2^/s (Fig. 4e and Supplementary Fig. 12). This isotropic exciton diffusion may originate from the similar distances between adjacent D-A units across different directions in T_S_-T_C_ (Supplementary Fig. 1c)^19^. It is evident that Br incorporation significantly enhances exciton diffusivity for both CT states, particularly for the CT_1_ state, whose diffusivity is nearly an order of magnitude greater than that in T_S_-T_C_ (Fig. 4e). Although the adjacent transport distances for the two CT states in T_Br_-T_C_ (7.90 Å and 7.93 Å) are larger than that of the CT_1_ state in T_S_-T_C_ (7.58 Å, Supplementary Fig. 1 and 2), exciton transport in T_Br_-T_C_ is nevertheless significantly enhanced. This improvement can be attributed to stronger intermolecular interactions and the enhanced electronic coupling, as reflected by the larger SOC matrix elements in T_Br_-T_C_ (Supplementary Table 3). Therefore, these findings indicate that the HAE enhances CT transport not through reduced intermolecular distances, but through strengthened electronic interactions and SOC, thereby providing direct dynamic evidence for HAE-facilitated exciton transport.

Furthermore, we also verified the universality of HAE-promoted triplet CT exciton transport across different heavy atoms and material systems. First, the chlorine atom was used as an alternative heavy atom instead of bromine. The T_Cl_-T_C_ (*trans*-4-chlorostilbene-1,2,4,5-tetracyanobenzene) cocrystal was successfully synthesized and exhibits notable ^3^CT exciton transport, with a diffusion coefficient of ~(1.0 ± 0.1)×10^-3 ^cm^2^/s (Supplementary Fig. 13). This value lies between those of T_S_-T_C_ ((3.5 ± 0.2) × 10^-4 ^cm^2^/s) and T_Br_-T_C_ ((3.3 ± 0.3)×10^-3 ^cm^2^/s). The systematic trend in diffusivity correlates with the increasing atomic number of the incorporated halogen (Cl<Br), consistent with progressively stronger SOC. This observation supports the conclusion that HAE directly promotes CT exciton transport. Second, we examined another donor-acceptor system, FR-T_C_ (fluorene-1,2,4,5-tetracyanobenzene) and its brominated analogue BrFR-T_C_ (2-bromocarbazole-1,2,4,5-tetracyanobenzene) cocrystals^47^. In this pair, significant broadening of the TADF intensity profiles is observed only in BrFR-T_C_, with a ^3^CT diffusion coefficient determined to be ~(7.3 ± 0.4)×10^-4 ^cm^2^/s (Supplementary Fig. 14). These results directly demonstrate the universality of HAE-induced CT transport enhancement, which is irrespective of different heavy atoms and distinct cocrystal systems, indicating that the strategy is not material-specific but broadly applicable.

### Modulation of CT transport dynamics via HAE

**Fig. 5:**
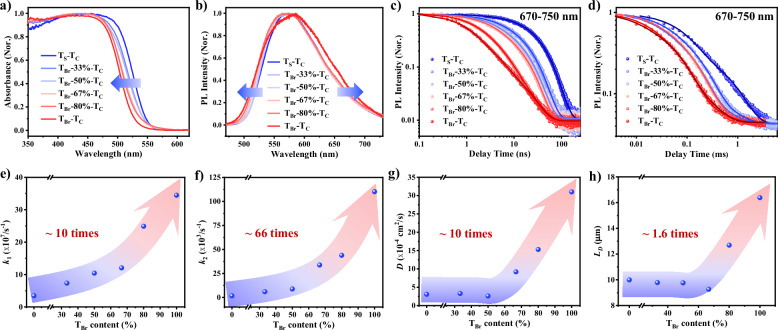
Optical properties and kinetic parameters of T_Br_-x-T_C_ cocrystals. **a** UV-Vis absorption and **b** PL spectra of T_Br_-x-T_C_ series. PL kinetics of the CT_1_ excitons in T_Br_-x-T_C_ series collected on **c** ns and **d** ms timescales. Solid lines in panels **c** and **d** are the multi-exponential fittings to these kinetics. The variation trends of **e** *k*_1_, **f** *k*_2_, **g** diffusion coefficient, and **h** diffusion distance of triplet CT_1_ state in T_Br_-x-T_C_ series with T_Br_ content along the optimal direction, as highlighted by shading.

Next, we advance our investigation by manipulating CT dynamics through the HAE. A series of ternary cocrystals with varying T_Br_ content (denoted as T_Br_-x-T_C_, x is the percentage of T_Br_ content) were synthesized (Supplementary Fig. 15), which exhibit structure changes gradually from T_S_-T_C_ to T_Br_-T_C_ (Supplementary Fig. 16). The absorption edge of T_Br_-x-T_C_ exhibits a continuous blueshift as T_Br_ content increases (Fig. 5a), and the corresponding PL emission is also gradually broadened with an increasingly pronounced double-peak feature (Fig. 5b). Figure 5c, d provide a detailed comparison of CT_1_ kinetics on both ns and ms timescales, respectively. These kinetics can be well fitted by triple-exponential functions, with detailed fitting parameters listed in Supplementary Table 4. Based on the experimentally determined rate constants of prompt fluorescence (${k}_{{PF}}$) and TADF (${k}_{{DF}}$), as well as their relative emission proportions (${\varphi }_{{PF}}$ and ${\varphi }_{{DF}}$, see Supplementary Note 2, Supplementary Fig. 17 and Table 5 for detailed discussion), the ISC and RISC rate constants (*k*_1_ and *k*_2_) can be determined (Supplementary Table 5, and see Supplementary Note 3 for more details). The calculated *k*_1_ in T_Br_-T_C_ is roughly consistent with the fitting result obtained by the transient absorption (TA) spectroscopy (Supplementary Fig. 18 and Table 6), indicating the reliability of our TADF-related analyses. The curves of *k*_1_ and *k*_2_ as a function of T_Br_ content are presented in Fig. 5e and f, both increasing remarkably above 50% T_Br_ content. Compared to T_S_-T_C_, T_Br_-T_C_ exhibits a 10-fold increase in *k*_1_ and a 66-fold increase in *k*_2_, clearly demonstrating the positive role of HAE in promoting TADF-related kinetics. The more efficient rate promotion of RISC compared to ISC is beneficial for the efficient extraction and utilization of triplet CT excitons, which is of great significance for promoting their application in optoelectronic fields^11,27,48^.

TADF imaging of the CT_1_ exciton diffusion was also conducted on T_Br_-x-T_C_ series (Supplementary Fig. 19), with the determined diffusivity along the direction with maximum migration efficiency illustrated in Fig. 5g. A distinct turning point also emerges at 50% T_Br_ content, beyond which the *D* value of CT_1_ state increases significantly, consistent with the variation trends of *k*_*1*_ and *k*_*2*_. This phenomenon arises from the SOC threshold effect, wherein a “stepwise enhancement” occurs once the SOC strength is sufficient to overcome the spin barrier when the heavy-atom content exceeds the threshold^49^. As discussed in our previous study, TADF imaging essentially reflects the diffusion of triplet CT excitons, driven by ^3^CT-dominated TADF kinetics^19^. Accordingly, the diffusion distance $\left({L}_{D}=2\sqrt{D\tau }\right)$ of the triplet CT_1_ (^3^CT_1_) state in T_Br_-x-T_C_ can be calculated based on the corresponding *D* values and triplet lifetimes (Supplementary Table 7). It is worth noting that, although the lifetime of ^3^CT_1_ excitons gradually decreases (Fig. 5d), the significantly enhanced *D* value in cocrystals with T_Br_ content above ~50% still enables long-distance ^3^CT_1_ transport, even surpassing that of T_S_-T_C_ (Fig. 5h). The ^3^CT_1_ excitons in T_Br_-T_C_ exhibit the largest *L*_*D*_ of 16.4 μm, which is ~1.6 times longer than that in T_S_-T_C_. As for the ^3^CT_2_ state, although it is expected to form upon the Br incorporation, it becomes clearly detectable only when T_Br_ content exceeds ~50% (Supplementary Figs. 20, 21). Similar to the ^3^CT_1_ state, the fitted ^3^CT_2_ diffusivity also increases progressively with increasing T_Br_ content (Supplementary Figs. 22, 23a), and reaches a maximum of ~1.6×10^-3 ^cm^2^/s in T_Br_-T_C_ ultimately (Fig. 4e). Based on the exponential fitting results of ^3^CT_2_ kinetics (Supplementary Fig. 24 and Table 8), the diffusion distance of ^3^CT_2_ excitons in T_Br_-x-T_C_ was calculated (Supplementary Table 7 and Fig. 23b), with a maximum of ~5.5 μm achieved at T_Br_ content exceeding 80%. These results clearly demonstrate that the TADF kinetics and CT transport can be synergistically modulated through HAE manipulation.

### Application of HAE-modulated long-distance CT exciton transport

**Fig. 6:**
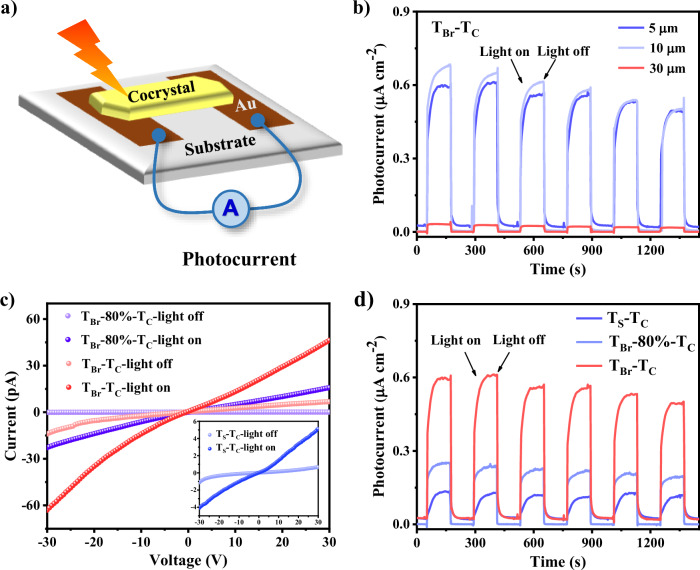
Photocurrent measurements on T_Br_-x-T_C_ cocrystals. **a** The schematic of the photocurrent measurements. **b** Time-dependent switching cycles of photocurrent response of T_Br_-T_C_ with electrode distances of 5 μm, 10 μm, and 30 μm. **c** The current-voltage curves and **d** time-dependent switching cycles of photocurrent response of T_S_-T_C_, T_Br_-80%-T_C_, and T_Br_-T_C_ with an electrode distance of 5 μm.

Achieving highly mobile and long-distance CT transport is crucial for developing efficient devices in photoelectronic applications^15–17,19^. Due to the efficient TADF in T_Br_-x-T_C_ series, the migrative ^3^CT_1_ excitons can regenerate into the singlet state through the effective RISC, thus resulting in an equivalent micron-scale singlet CT transport through a triplet-assisted mechanism (Fig. 1). By adjusting the T_Br_ content, this triplet-assisted CT transport can be further promoted through the enhanced SOC intensity (Fig. 5e–h). To evaluate the contribution of CT transport to the optoelectronic device performance, photocurrent measurements were conducted as a prototypical investigation (Fig. 6a and see Methods for details). As shown in Fig. 6b, the photocurrent density of T_Br_-T_C_ remains approximately constant when the electrode spacing (5 μm and 10 μm) is smaller than its average CT migration distance of ~16 μm. However, a noticeable decrease in photocurrent is observed when the electrode spacing (30 μm) exceeds this diffusion length. The CT excitons in T_Br_-T_C_ remain strongly bound even under a bias voltage of 20 V, as evidenced by the power-law dependence of PL intensity on excitation intensity (Supplementary Fig. 25)^50,51^. Efficient charge extraction at the electrode interfaces is also ensured by the favorable contact between the cocrystal and electrodes (Supplementary Fig. 26). These results directly confirm that the long-distance CT exciton transport, rather than the drift of free carriers, is the key factor governing the photocurrent response in CT cocrystals. Furthermore, a significantly enhanced photocurrent response was observed with increasing T_Br_ content (Fig. 6c). The photocurrent densities of T_Br_-80%-T_C_ and T_Br_-T_C_ are notably improved, showing approximately 2.3- and 6.0-fold enhancements compared to that of T_S_-T_C_, respectively (Fig. 6d). These findings further demonstrate that promoting long-distance CT transport through the heavy-atom functionalization holds great promise for effectively enhancing the performance of optoelectronic devices.

In summary, we have demonstrated that Br-atom functionalization is a highly effective strategy for promoting CT exciton transport in TADF cocrystals. The enhanced SOC intensity in T_Br_-T_C_ cocrystal not only accelerates the ISC and RISC kinetics but also substantially enhances CT exciton transport: the diffusion coefficient increases by an order of magnitude, and the diffusion length extends by 1.6 times compared with T_S_-T_C_. Furthermore, the CT transport dynamics and the resulting photocurrent responses can be effectively modulated by varying the T_Br_ content. Our investigations provide compelling evidence for manipulating exciton transport in organic semiconductors, highlighting heavy-atom engineering as an effective strategy for advancing high-performance optoelectronic applications.

## Methods

### Synthesis of T_S_-T_C_ cocrystal

A mixture of T_S_ (18.0 mg, 0.1 mmol) and T_C_ (35.6 mg, 0.2 mmol) was dissolved in 10 ml acetonitrile solvent and directly dropped onto the substrate. After the complete evaporation of solvent, yellow ribbon-like T_S_-T_C_ cocrystals were obtained.

### Synthesis of T_Br_-T_C_ cocrystal

A mixture of T_Br_ (25.9 mg, 0.1 mmol) and T_C_ (35.6 mg, 0.2 mmol) was dissolved in 10 ml acetonitrile solvent and directly dropped onto the substrate. After the complete evaporation of solvent, yellow ribbon-like T_Br_-T_C_ cocrystals were obtained.

### Synthesis of T_Br_-x-T_C_ cocrystals

For T_Br_-33%-T_C_, a mixture of T_S_ (12.1 mg, 0.067 mmol), T_Br_ (8.5 mg, 0.033 mmol) and T_C_ (35.6 mg, 0.2 mmol) was dissolved in 10 ml acetonitrile solvent and directly dropped onto the substrate. Yellow ribbon-like cocrystals were obtained after the complete evaporation of solvent. For T_Br_-50%-T_C_, a mixture of T_S_ (9.0 mg, 0.05 mmol), T_Br_ (13.0 mg, 0.05 mmol) and T_C_ (35.6 mg, 0.2 mmol) was used; for T_Br_-67%-T_C_, a mixture of T_S_ (5.9 mg, 0.033 mmol), T_Br_ (17.4 mg, 0.067 mmol) and T_C_ (35.6 mg, 0.2 mmol) was used; for T_Br_-80%-T_C_, a mixture of T_S_ (3.6 mg, 0.02 mmol), T_Br_ (20.7 mg, 0.08 mmol) and T_C_ (35.6 mg, 0.2 mmol) was used for the synthesis, while other conditions remained the same.

### Material characterizations

X-ray diffraction (XRD) pattern was obtained by using a X’pert Pro X-Ray Diffractometer (PANAlytical, Netherlands) using Cu Kα radiation. A scan rate of 5^o^ min^-1^ was applied in the range of 5–40^o^. Diffraction intensity data for single crystals of T_Br_-T_C_ was collected on a GeminiUltra diffractometer equipped with graphite-monochromatic Mo Kα radiation (λ = 0.71073 Å). The structure of T_Br_-T_C_ was solved by direct methods and refined by full-matrix least-squares techniques based on F^2^ using the SHELXS-97 programs. All the non-hydrogen atoms were refined with anisotropic parameters, while hydrogen atoms were placed in calculated positions and refined using a riding model. UV-Vis diffuse reflectance spectra (DRS) were recorded using a UV-Vis spectrophotometer (JASCO V-550) equipped with an integrating sphere, and BaSO_4_ powder was used as the reference for the baseline correction. ^1^H-NMR measurements were carried out using an AVANCE III HD 700 MHz liquid nuclear magnetic resonance spectrum with DMSO-d6 as the solvent.

### PL and delayed PL measurements

The PL kinetic measurements on a nanosecond timescale were performed on a home-built PL-scanned imaging microscopy coupled with a time-correlated single photon counting (TCSPC) module. A 375 nm pulse laser (PDL 800-B, PicoQuant) was focused on the sample through a 100× air objective lens (NA = 0.95, Olympus PLFLN) with the spot radius of ~565 nm. The excitation intensity is adjusted by a neutral density filter and measured with a power meter (PM100D S130VC, Thorlabs, USA). By fixing the excitation spot at a selected position on the sample, the PL emission from the entire sample can be collected using the fast rotation of a pair of galvanometer mirrors. PL kinetics were collected with a high-speed detector (HPM-100-50, Hamamatsu, Japan) equipped with appropriate bandpass filters. The steady-state PL emission spectra were obtained by a monochromator (SpectraPro-HRS-300, Princeton Instruments, USA) coupled with a charge-coupled device (CCD) camera (PIXIS 100, Princeton Instruments, USA).

The delayed PL imaging and kinetic measurements were performed on the same setup with an on-off modulated 375 nm pulse laser for excitation. The scanning images of delayed PL contain 256×256 pixels (64 nm/pixel). For delayed PL kinetics on the ms scale, a multipulse-excited TCSPC phosphorescence decay recording technique was employed to improve the detection sensitivity^19,52^. The delayed PL kinetics was collected by a high-speed detector (HPM-100-50, Hamamatsu, Japan) equipped with appropriate band-pass filters.

For measurements of temperature-dependent PL and delayed PL kinetics, a 100× air objective lens with NA = 0.6 (Olympus SLMPlan N) was used for instead.

The delayed PL spectra were measured by an ICMOS (Intensified Complementary Metal Oxide Semiconductor) camera (TRC411-S-HQB-F, Intelligent Scientific Systems, China). A 343 nm pulse laser was used for excitation. The delay time was controlled by a digital delay pulse generator (DG645, Stanford Research Systems, USA), and the gate width was set to 8 ms.

### Ultrafast transient absorption spectroscopy measurements

The femtosecond transient absorption (fs-TA) setup is based on a regenerative amplified Ti:sapphire laser system from Coherent (800 nm, 35 fs, 6 mJ/pulse, and 1 kHz repetition rate), nonlinear frequency mixing techniques, and the Femto-TA100 spectrometer (Time-Tech Spectra). The 800 nm output pulse from the regenerative amplifier was split into two parts with a 50% beam splitter. The transmitted part was used to pump a TOPAS Optical Parametric Amplifier (OPA), which generates a 350 nm laser pulse as the pump beam. The reflected 800 nm beam was attenuated with a neutral density filter and focused into a 2 mm thick CaF_2_ window to generate a white light continuum (WLC) from 340 nm to 800 nm, used for the probe beam. After the sample, the probe beam was collimated and then focused into a fiber-coupled spectrometer with CMOS sensors and detected at a frequency of 1 kHz. The delay between the pump and probe pulses was controlled by a motorized delay stage. The pump pulses were chopped by a synchronized chopper at 500 Hz, and the absorbance change was calculated with two adjacent probe pulses (pump-blocked and pump-unblocked). All experiments were performed at room temperature.

### Photocurrent response measurements

The single crystal of cocrystal was directly placed on the gold electrode with the interelectrode distance of 5 μm, 10 μm, and 30 μm, respectively. The current-voltage (I-V) and photocurrent-time (I-t) curves under light-on and light-off conditions were measured by a semiconductor parameter analyzer (Tektronix, Keithley 4200-SCS). For the measurement of I-t curves, the voltage was set to a fixed value of 20 V, and a halogen lamp was used for excitation.

### Theoretical calculations

All simulation calculations were carried out using the Gaussian 09 program package. Density functional theory (DFT) calculations on the geometrical and electronic properties of the ground state were performed using the B3LYP density functional method with the basis set genecp (C, H, N for 6-31 g(d,p) and Br for lanl2dz). Time-dependent DFT (TD-DFT) calculations were also performed using this method.

## Supplementary information

Supplementary Information

## Source data

Source Data

Transparent Peer Review file

## Data Availability

The crystallographic data of both T_S_-T_C_ and T_Br_-T_C_ generated in this study have been deposited in the Cambridge Crystallographic Data Centre (CCDC) database under CCDC numbers 2521586 and 2521253 [www.ccdc.cam.ac.uk/data_request/cif]. All raw data generated within the article and the Supplementary Information are available from the Source Data File. All data are available from the corresponding author upon request. Source data are provided with this paper.
