# Supplementary material for: Heavy-atom functionalization promotes triplet-assisted charge-transfer exciton transport in organic cocrystals
Source: Nat Commun. 2026 Apr 22;17:5533. doi: 10.1038/s41467-026-72317-8 (PMC13287730; doi:10.1038/s41467-026-72317-8)
Supplement: Supplementary file 1 — Supplementary Information [file 41467_2026_72317_MOESM1_ESM.pdf]

Supplementary Information for

Heavy-atom functionalization promotes triplet-assisted charge-transfer exciton transport in organic cocrystals

Yejun Xiao*,[1], Yaxiong Wei[2], Min Zhang[1,3], Rui Cai[4], Xuan Liu[5], Peng Xu[1], Shengye Jin[1], Jing Leng*,[1], and Wenming Tian*,[1]

[1] State Key Laboratory of Chemical Reaction Dynamics, Dalian Institute of Chemical Physics, Chinese Academy of Sciences

Dalian 116023, China

[2] Anhui Province Key Laboratory for Control and Applications of Optoelectronic Information Materials, School of Physics and Electronic Information, Anhui Normal University

Wuhu 241002, China

[3] University of Chinese Academy of Sciences

Beijing 100049, China

[4] Instrumental Analysis Center, Dalian University of Technology

Dalian 116024, China

[5] State Key Laboratory of Photoelectric Conversion and Utilization of Solar Energy, Dalian National Laboratory for Clean Energy, Dalian Institute of Chemical Physics, Chinese Academy of Sciences

Dalian 116023, China

Corresponding Author

yjxiao@dicp.ac.cn

ljjx@dicp.ac.cn

tianwm@dicp.ac.cn

Supplementary Note 1

Estimation of exciton diffusion parameters.

The exciton diffusion in lateral dimension can be described by a two-dimensional (2D) diffusion equation:

$$\frac{\partial n(x,y,t)}{\partial t} = D\left\{\frac{\partial^2 n(x,y,t)}{\partial x^2} + \frac{\partial^2 n(x,y,t)}{\partial y^2}\right\} - k_1 n(x,y,t) \quad (1)$$

where $n(x, y, t)$ is the concentration of excitons at time t and position (x, y); D is the diffusion coefficient; k_1 is the first order recombination coefficient. As the exciton population follows a time-dependent 2D Gaussian-type distribution due to the Gaussian pump beam:

$$I_{PL}(x,y,t) \propto n(x,y,t) = Nexp\left[-\frac{(x-x_0)^2}{2\sigma_{x,t}^2} - \frac{(y-y_0)^2}{2\sigma_{y,t}^2}\right] \quad (2)$$

where x_0 and y_0 are the central position of Gaussian excitation beam; $\sigma_{x,t}^2$ and $\sigma_{y,t}^2$ are the time-dependent deviation of Gaussian profiles along x and y directions. Therefore, a general solution to Supplementary Equation 1 is:

$$n(x,y,t) = \frac{1}{4\pi Dt}\exp\left(-\frac{x^2+y^2}{4Dt}\right) \quad (3)$$

In terms of Supplementary Equations 2 and 3, the Gaussian variance of 1D PL profile extracted from PL image (i.e., the cross section of PL image) can be directly related to the diffusion coefficient D as follows:

$$\sigma_{x,t}^2 = \sigma_{x,0}^2 + l_x^2 = \sigma_{x,0}^2 + 2Dt \quad (4)$$

where l_x is the distance of exciton diffusing away (along x-direction) from the initial position at delay time t. Therefore, the diffusion coefficient D can be obtained from the linear fitting of Gaussian variances at different times:

$$D = \frac{\sigma_{x,t}^2 - \sigma_{x,0}^2}{2t} = \frac{l_x^2}{2t} \quad (5)$$

and the 2D diffusion distance L_D can be estimated by $L_D = \sqrt{l_x^2 + l_y^2} = 2\sqrt{D\tau}$.

Supplementary Note 2

Estimation of the relative emission proportion of prompt fluorescence (PF) and TADF components (φ_{PF} and φ_{DF}) in T_S-T_C and T_{Br}-x-T_C cocrystals.

The TADF intensity under single-pulse excitation can be described the following equation:

$$I_{DF}(t) = I_{DF_0} exp\left(-\frac{t}{\tau_{DF}}\right) = \eta I_0 exp\left(-\frac{t}{\tau_{DF}}\right) \quad (6)$$

where I_{DF_0} is the initial TADF intensity at $t = 0$; τ_{DF} is the lifetime of TADF emission; η is the weight of TADF intensity relative to PF intensity at $t = 0$ under single-pulse excitation; I_0 is the initial PF intensity at $t = 0$.

Under multi-pulse excitation (i.e., based on the principle of multipulse-excited TCSPC PDR technique),[1,2] the accumulated TADF intensity can be written as:

$$\begin{aligned} I_{DF}(t) &= I_{DF_0} exp\left(-\frac{t}{\tau_{DF}}\right) + I_{DF_0} exp\left(-\frac{t+T}{\tau_{DF}}\right) + \cdots + I_{DF_0} exp\left(-\frac{t+nT}{\tau_{DF}}\right) \\ &= I_{DF_0} exp\left(-\frac{t}{\tau_{DF}}\right)\left[1 + exp\left(-\frac{T}{\tau_{DF}}\right) + \cdots + exp\left(-\frac{nT}{\tau_{DF}}\right)\right] \\ &= I_{DF_0} exp\left(-\frac{t}{\tau_{DF}}\right)\frac{1-exp\left(-\frac{nT}{\tau_{DF}}\right)}{1-exp\left(-\frac{T}{\tau_{DF}}\right)} \end{aligned} \quad (7)$$

where T is the time interval between two pulse excitations; n is the number of the multi-pulse excitation. As $nT >> \tau_{DF}$, the Supplementary Equation 7 can be approximated as:

$$I_{DF}(t) \approx \frac{I_{DF_0} exp\left(-\frac{t}{\tau_{DF}}\right)}{1-exp\left(-\frac{T}{\tau_{DF}}\right)} \quad (8)$$

Accordingly, the maximum TADF intensity at $t = 0$ can be obtained:

$$I_{max} = I_{DF}(0) = \frac{I_{DF_0}}{1-exp\left(-\frac{T}{\tau_{DF}}\right)} = \frac{\eta I_0}{1-exp\left(-\frac{T}{\tau_{DF}}\right)} \quad (9)$$

When $T << \tau_{DF}$, $1 - exp\left(-\frac{T}{\tau_{DF}}\right)$ roughly equals to $\frac{T}{\tau_{DF}}$. Therefore, the weight of TADF can be estimated by:

$$\eta = \frac{I_{max}T}{I_0\tau_{DF}} \quad (10)$$

and the relative emission proportion of PF and TADF components (φ_{PF} and φ_{DF}) to the photoluminescence quantum yield ($PLQY$) can be calculated by:

$$\varphi_{DF} = \frac{\emptyset_{DF}}{PLQY} = \frac{\eta\tau_{DF}}{\eta\tau_{DF}+(1-\eta)\tau_{PF}} \quad (11)$$

$$\varphi_{PF} = \frac{\emptyset_{PF}}{PLQY} = 1 - \varphi_{DF} \quad (12)$$

where $PLQY = \emptyset_{PF} + \emptyset_{DF}$; and the $\emptyset_{PF}$ and $\emptyset_{DF}$ are the realistic efficiencies of PF and TADF emission components, respectively.

In our cases, the average lifetime of PF (τ_{ave} in Supplementary Table 4) was used as τ_{PF} for calculation, and the long-lived TADF component of τ_3 in Supplementary Table 4 was interpreted as the intrinsic τ_{DF}. Moreover, based on the experimentally obtained ratio of I_{max}/I_0 (Supplementary Fig. 17), the parameters of η, φ_{PF} and φ_{DF} can be calculated. The resulting values are summarized in Supplementary Table 5.

Supplementary Note 3

Estimation of rate constants in T_S-T_C and T_{Br}-x-T_C cocrystals.

Based on the possible photophysical processes in T_{Br}-T_C as illustrated in Fig. 3d, and assuming that the dynamic processes related to CT_1 and CT_2 states are independent of each other, the rate equation of singlet CT_1 ($^1CT_1$) state can be written as:[2]

$$\frac{dn(^1\mathrm{CT}_1)}{dt} = -\left(\frac{k_0k_2+k_1k_3}{k_1+k_2}\right) n(^1\mathrm{CT}_1) \quad (13)$$

$$k_{steady} = k_{DF} = \frac{k_0k_2+k_1k_3}{k_1+k_2} \quad (14)$$

where k_{DF} is the rate constant of the TADF decay.

When $k_1 \gg k_2$, Supplementary Equation 14 can be reduced

$$k_{DF} = k_3 + k_0\frac{k_2}{k_1} \quad (15)$$

Under steady-state approximation, the $\frac{k_2}{k_1}$ can be further calculated by:

$$\frac{k_2}{k_1} = \frac{n(^1\mathrm{CT}_1)}{n(^3\mathrm{CT}_1)} = \frac{\emptyset_{DF}k_{DF}}{\emptyset_{PF}k_{PF}} = \frac{\varphi_{DF}k_{DF}}{\varphi_{PF}k_{PF}} \quad (16)$$

where k_{PF} is the rate constant of prompt fluorescence decay. $\emptyset_{PF}$ and $\emptyset_{DF}$ can be written as:

$$k_{PF} = k_0 + k_1 \tag{17}$$

$$\emptyset_{PF} = \frac{k_0^r}{k_0+k_1} \tag{18}$$

$$\emptyset_{DF} = \frac{k_1}{k_0+k_1} \cdot \frac{k_0^r k_2}{k_0 k_2 + k_1 k_3} = \frac{k_0^r}{k_0+k_1} \cdot \frac{k_1 k_2}{k_0 k_2 + k_1 k_3} = \frac{k_0^r}{k_0+k_1} \cdot \frac{k_2}{k_0 \frac{k_2}{k_1} + k_3} \tag{19}$$

where k_0^r is the radiative recombination rate constant of the singlet CT_1 state. Putting Supplementary Equations 15 and 18 into 19, $\emptyset_{DF}$ can be written as:

$$\emptyset_{DF} = \emptyset_{PF} \cdot \frac{k_2}{k_{DF}} \tag{20}$$

Furthermore, according to Supplementary Equations 11 and 12, k_2 can be written as:

$$k_2 = \frac{\emptyset_{DF}}{\emptyset_{PF}} \cdot k_{DF} = \frac{\varphi_{DF}}{\varphi_{PF}} \cdot k_{DF} \tag{21}$$

Based on the experimentally obtained values of k_{PF}, k_{DF}, φ_{PF} and φ_{DF} (listed in Supplementary Table 5), the rate constants of ISC (k_1) and RISC (k_2) processes in T_{Br}-T_C can be calculated. The kinetic model of CT excitons in T_S-T_C and T_{Br}-x-T_C series cocrystals is consistent with that of T_{Br}-T_C. Accordingly, a series of k_1 and k_2 values were obtained for T_S-T_C and T_{Br}-x-T_C, as listed in Supplementary Table 5.

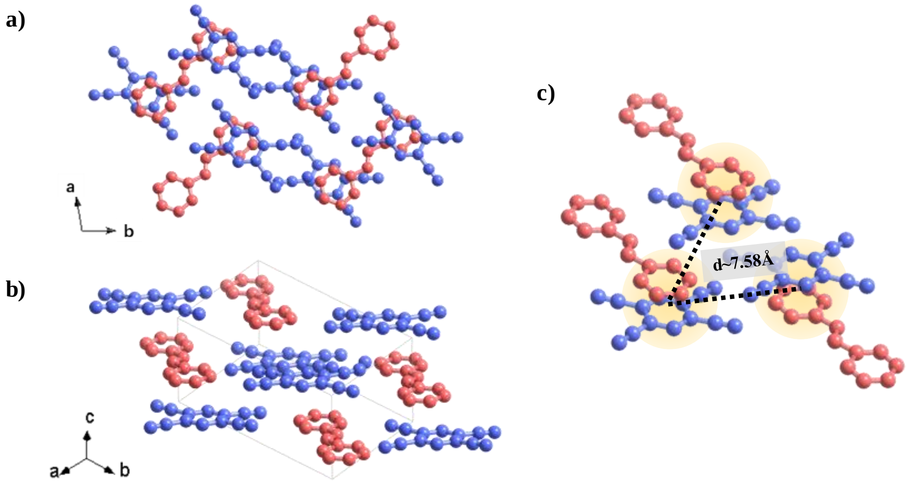

Supplementary Fig. 1 Single crystal structure of T_S-T_C viewed along the (a) *c* axis and (b) diagonal direction of the *a* and *b* axes. (c) The exciton transport distance of ~7.58 Å between the adjacent CT_1 states in T_S-T_C. Color representation: red, TSB; blue, TCNB.

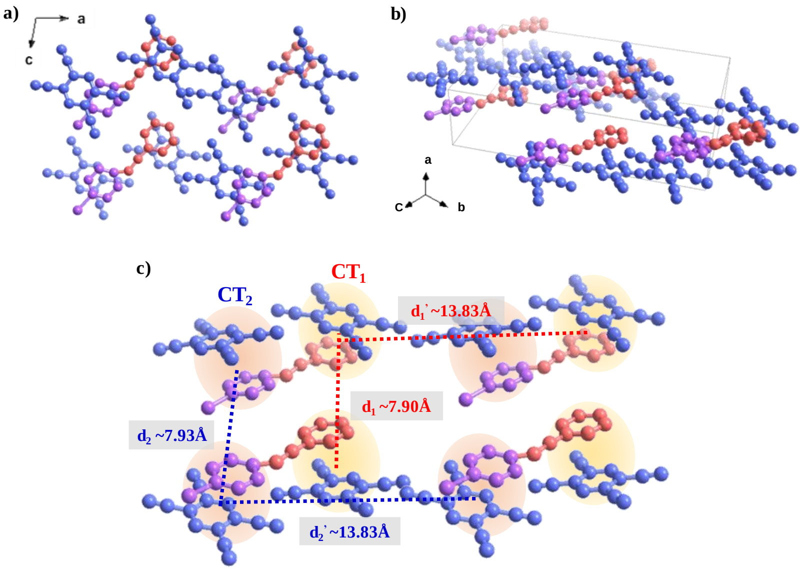

Supplementary Fig. 2 Single crystal structure of T_{Br}-T_C viewed along the (a) *b* axis and (b) diagonal direction of the *b* and *c* axes. (c) The anisotropic exciton transport distances for CT_1 and CT_2 states in T_{Br}-T_C. Color representation: purple and red, parts of Br-incorporated TSB molecule with and without Br functionalization; blue, TCNB.

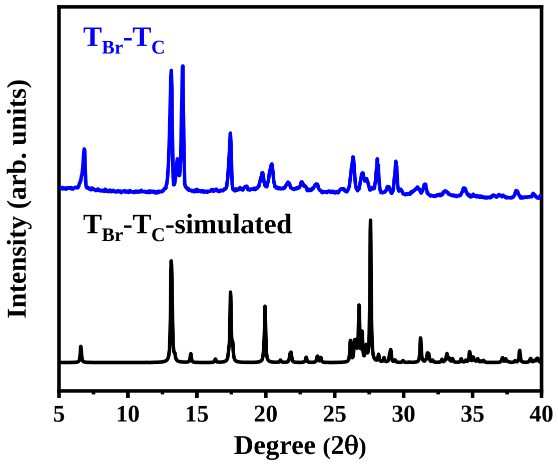

Supplementary Fig. 3 XRD patterns of T_{Br}-T_C and T_{Br}-T_C-simulated.

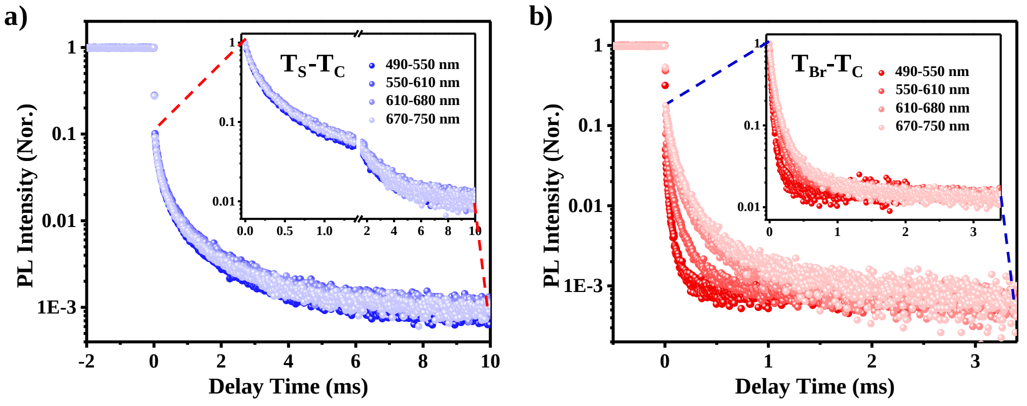

Supplementary Fig. 4 Comparison of ms-scale PL kinetics of (a) T_S-T_C and (b) T_{Br}-T_C collected at different emission wavelengths. Insert of (a) and (b) show the comparison of delayed PL kinetics after deducting initial fluorescence interference.

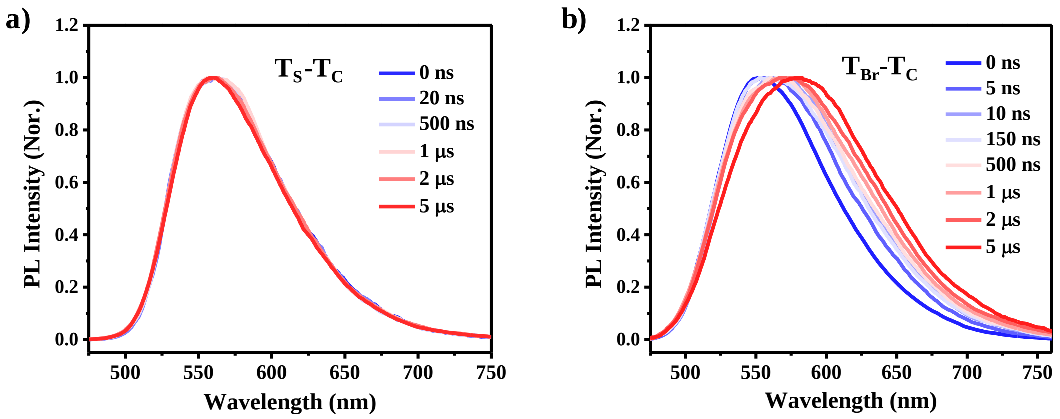

Supplementary Fig. 5 Normalized PL spectra of (a) T_S-T_C and (b) T_{Br}-T_C at different delay times measured by ICMOS camera under the 343 nm excitation. The constant PL emission spectra of T_S-T_C indicates a single-component CT emission, while the continuous redshift of emission peak from ~555 nm to ~585 nm suggests the multi-component emission in T_{Br}-T_C.

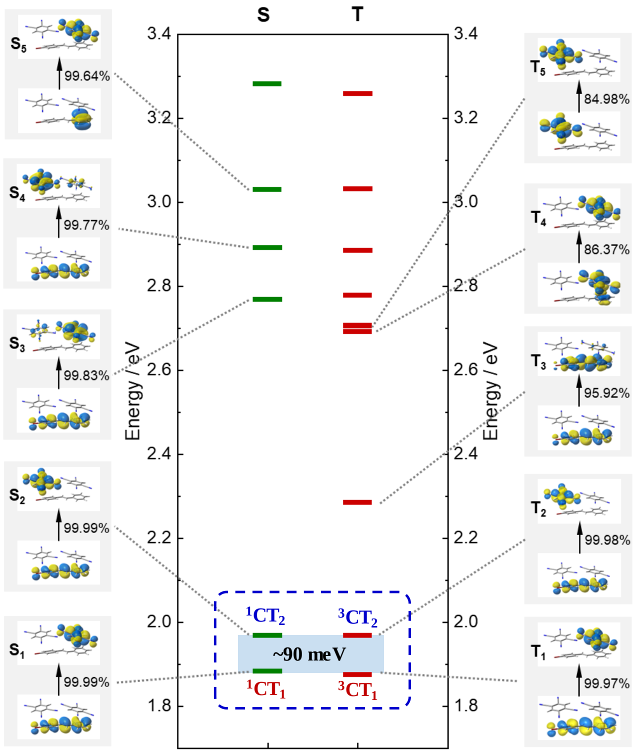

Supplementary Fig. 6 Computed vertical energy levels in T_{Br}-T_C. Selected states are illustrated by NTOs. State characters are interpreted and occupation numbers are indicated. Detailed calculation parameters are listed in Supplementary Table 2.

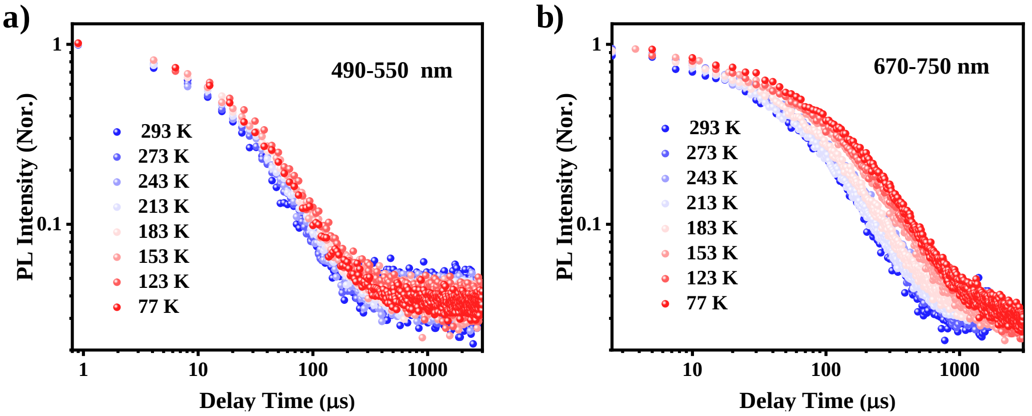

Supplementary Fig. 7 Comparison of temperature-dependent ms-scale delayed PL kinetics of CT_2 and CT_1 excitons in T_{Br}-T_C, collected at (a) 490-550 nm and (b) 670-750 nm, respectively. The CT_2 kinetics in T_{Br}-T_C shows almost identical profiles at different temperatures, whereas CT_1 excitons exhibit significantly prolonged lifetimes at temperatures below 213 K.

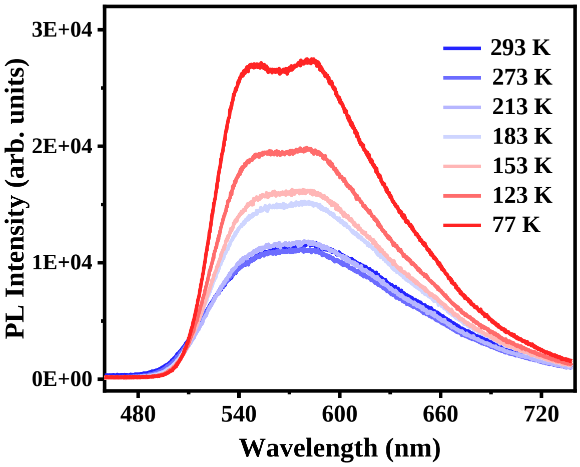

Supplementary Fig. 8 Comparison of temperature-dependent PL spectra of T_{Br}-T_C under the 375 nm excitation, showing a notable intensity increase below 213 K.

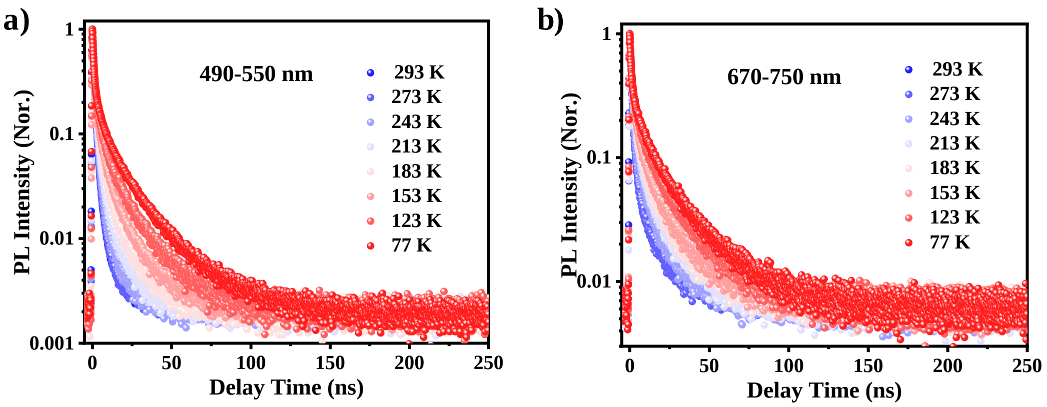

Supplementary Fig. 9 Comparison of temperature-dependent ns-scale PL kinetics of CT_2 and CT_1 states in T_{Br}-T_C, collected at (a) 490-550 nm and (b) 670-750 nm, respectively. The prolonged PL lifetimes observed at low temperatures indicate enhanced luminescence efficiency of T_{Br}-T_C.

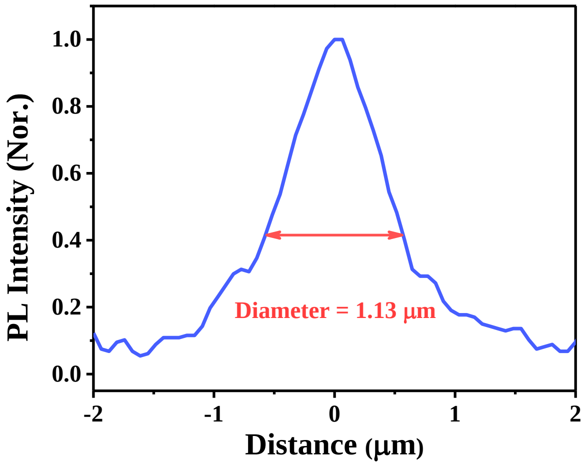

Supplementary Fig. 10 Diameter of the initial PL distribution at t = 0 ns after the focused excitation, estimated to be ~1.13 μm.

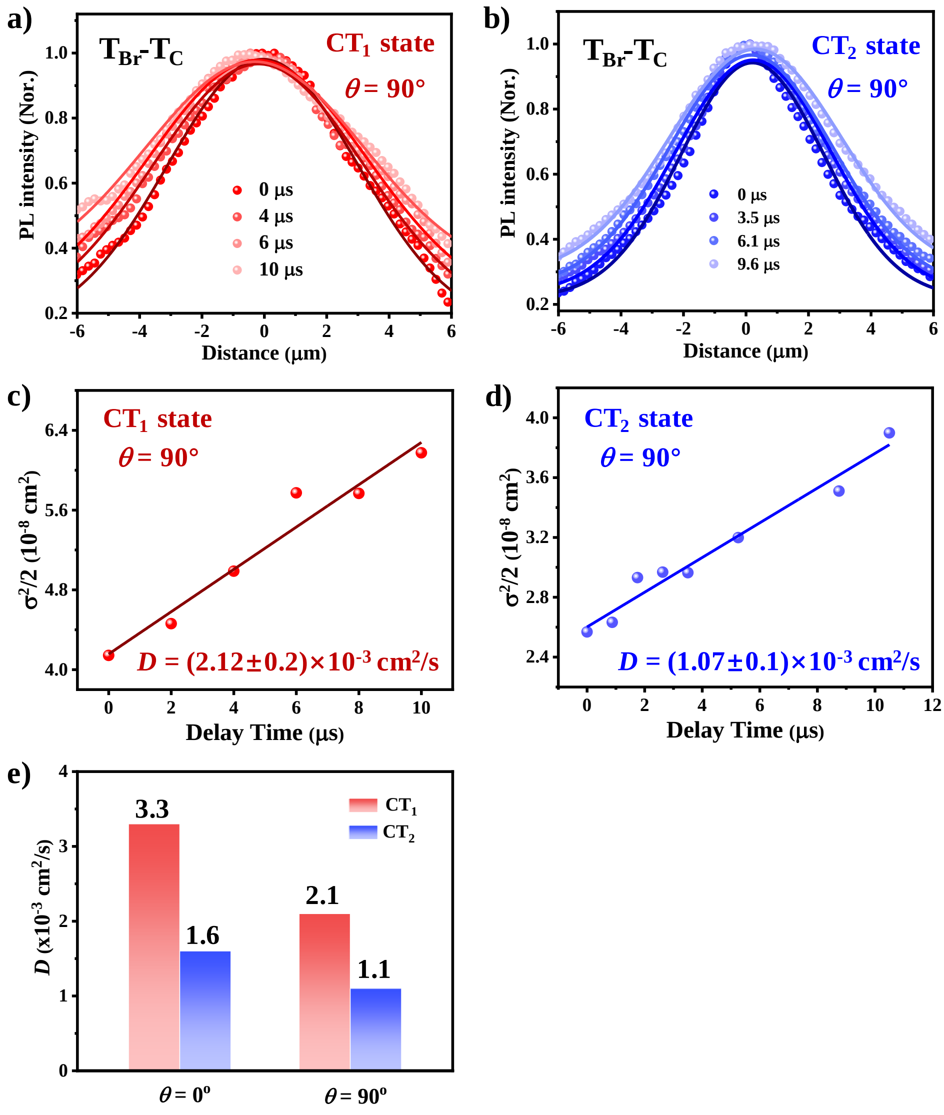

Supplementary Fig. 11 The anisotropic CT exciton transport in T_{Br}-T_C collected at 490-550 nm (CT_2 state) and 670-750 nm (CT_1 state), respectively. Normalized time-dependent 1D TADF intensity profiles of (a) CT_1 and (b) CT_2 states in T_{Br}-T_C extracted from the $\theta = 90°$ direction as shown in Fig. 4a and 4b, respectively. The solid lines are their Gaussian fittings. The determination of TADF-related exciton diffusion coefficient for the (c) CT_1 and (d) CT_2 states in T_{Br}-T_C along the $\theta = 90°$ direction. (e) The comparison of CT_1 and CT_2 exciton transport along different directions in T_{Br}-T_C.

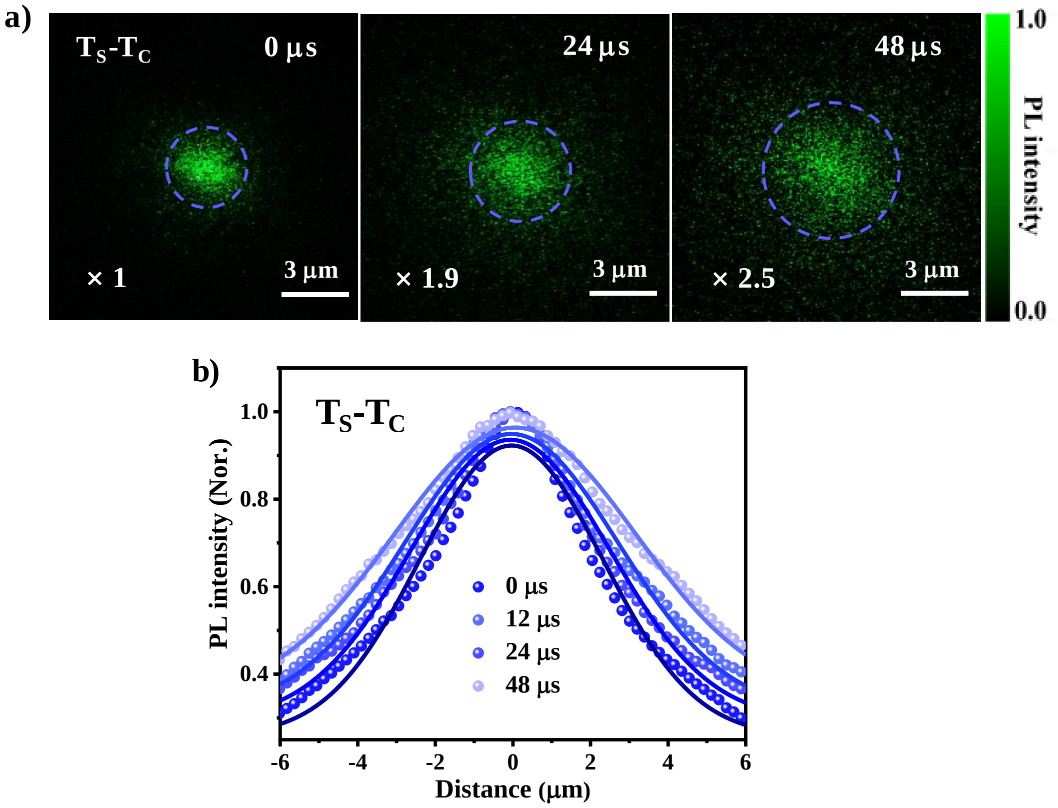

Supplementary Fig. 12 TADF imaging of the CT_1 state in T_S-T_C. (a) TADF intensity images of T_S-T_C at different delay times after excitation. Scale bars are 3 μm. (b) Normalized 1D TADF intensity profiles extracted from panel a, along with their Gaussian fittings, showing the spatial broadening of the TADF-related exciton distribution.

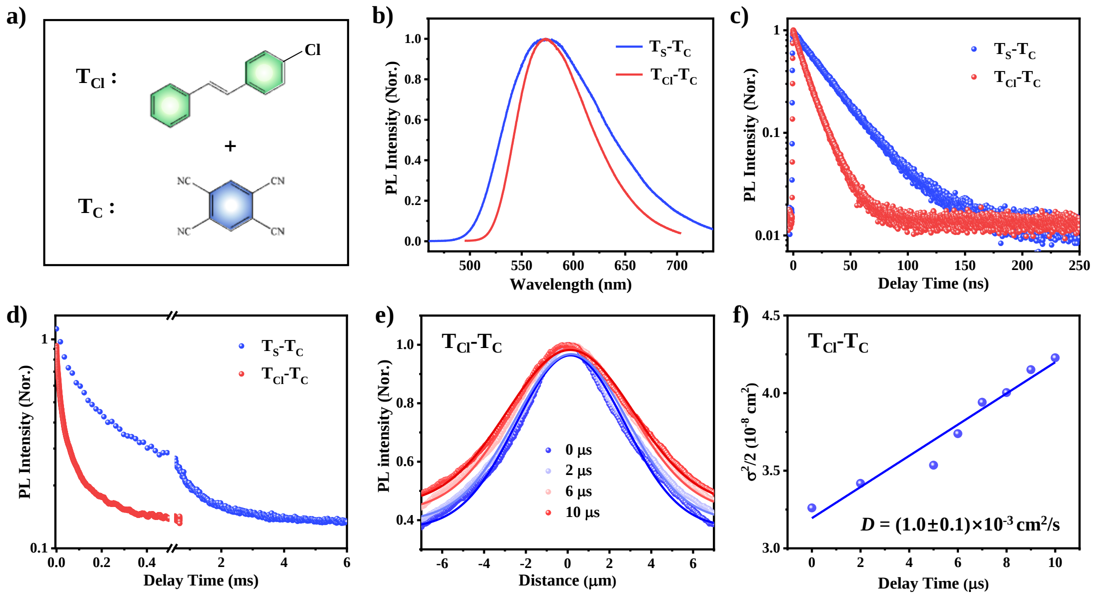

Supplementary Fig. 13 Imaging and modeling of CT exciton transport in T_{Cl}-T_C. (a) Chemical structures of T_{Cl} and T_C. Comparison of the normalized (b) PL spectra, (c) PL kinetics on ns timescale, and (d) PL kinetics on ms timescale between T_S-T_C and T_{Cl}-T_C. (e) Normalized 1D TADF intensity profiles of T_{Cl}-T_C at different delay times, along with their Gaussian fittings. (f) The determination of the ^{3}CT diffusion coefficient through linearly fitting of the 1D time-dependent Gaussian variances ($\sigma^2(t)$).

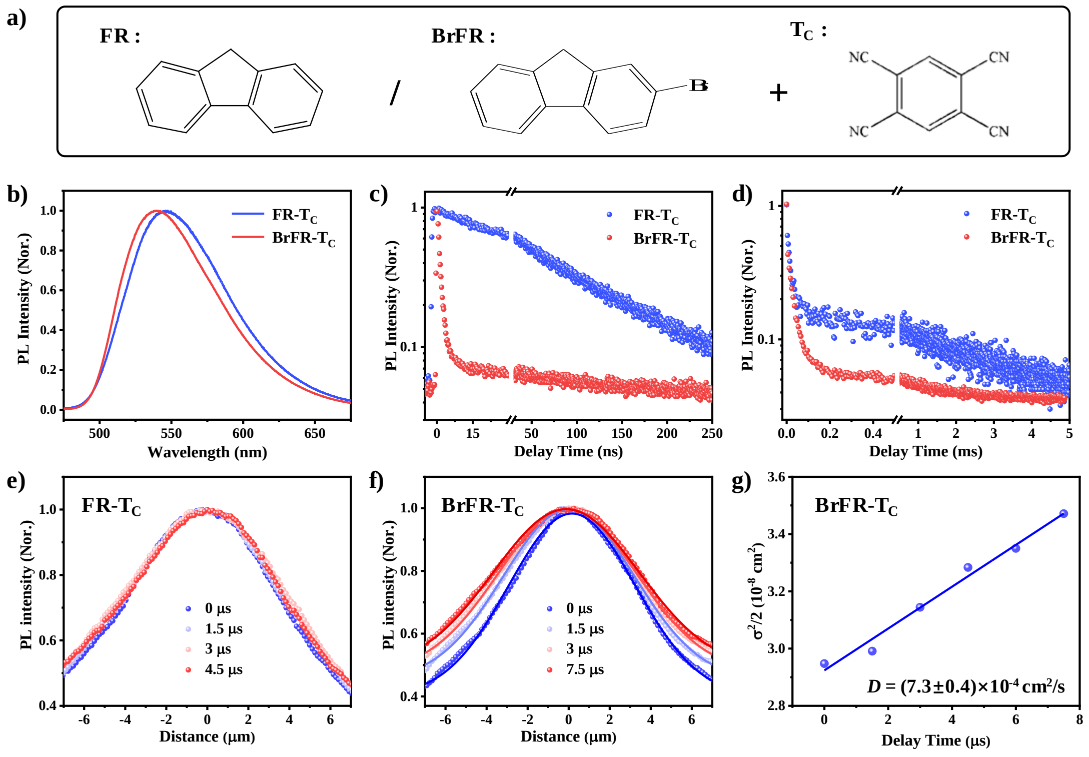

Supplementary Fig. 14 Imaging and modeling of CT exciton transport in FR-T_C and BrFR-T_C. (a) Chemical structures of FR, BrFR and T_C. Comparison of the normalized (b) PL spectra, (c) PL kinetics on ns timescale, and (d) PL kinetics on ms timescale between FR-T_C and BrFR-T_C. Normalized 1D TADF intensity profiles of (e) FR-T_C and (f) BrFR-T_C at different delay times, along with their Gaussian fittings. (f) The determination of the ^{3}CT diffusion coefficient in BrFR-T_C through linearly fitting of the 1D time-dependent Gaussian variances ($\sigma^2(t)$).

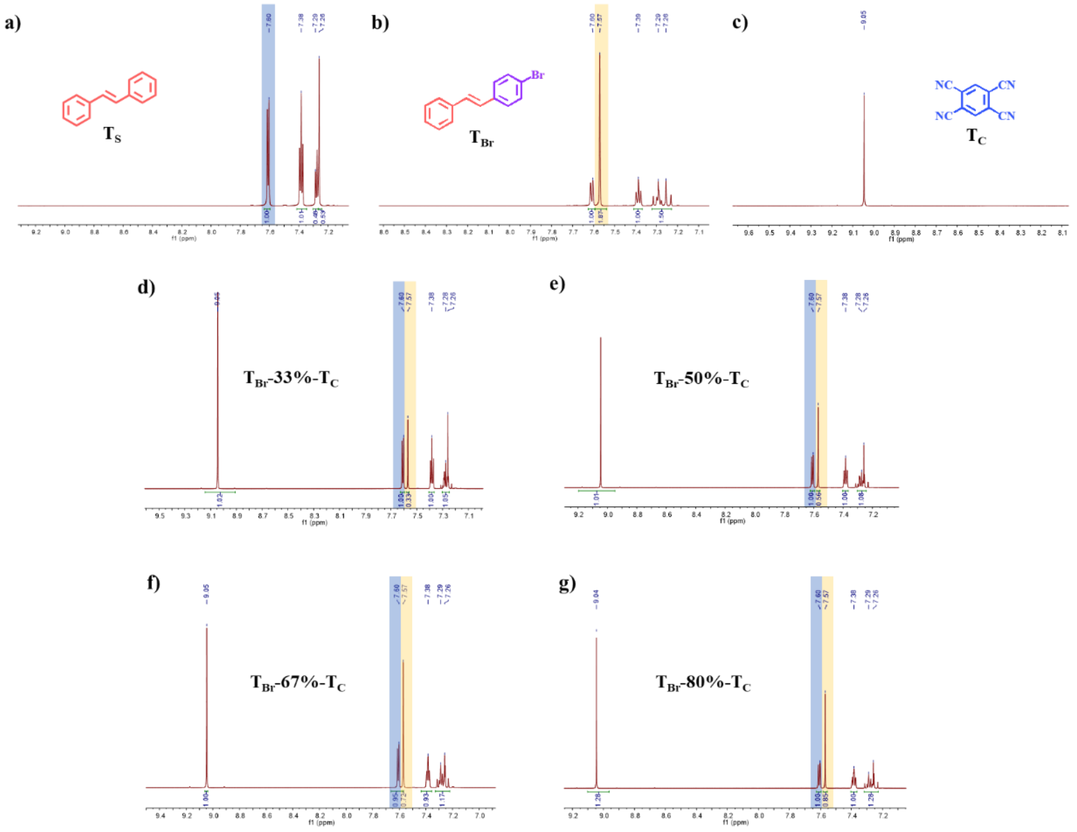

Supplementary Fig. 15 ^{1}H-NMR spectra of (a) T_S; (b) T_{Br}; (c) T_C; (d) T_{Br}-33%-T_C; (e) T_{Br}-50%-T_C; (f) T_{Br}-67%-T_C and (g) T_{Br}-80%-T_C. Based on the characteristic peaks of T_S at 7.60 (blue shading) and the T_{Br} parts containing Br group at 7.57 (yellow shading), the actual T_{Br} content in T_{Br}-x-T_C series can be determined by calculating the area ratio of these two peaks, which closely approximates the feed ratio.

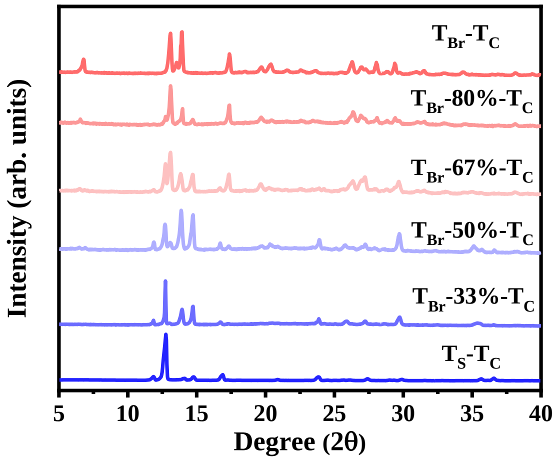

Supplementary Fig. 16 XRD patterns of T_S-T_C and T_{Br}-x-T_C cocrystals, showing the gradual structural changes from T_S-T_C to T_{Br}-T_C.

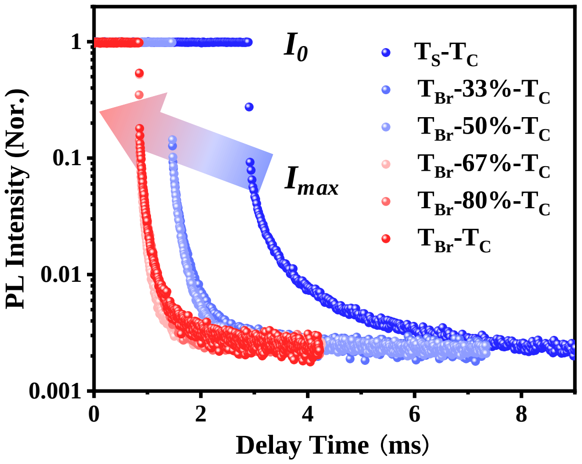

Supplementary Fig. 17 Millisecond-scale PL kinetics of the CT_1 excitons in T_S-T_C and T_{Br}-x-T_C cocrystals collected at 690-750 nm. The TADF intensity at the second data point after the laser off was interpreted as the I_{max} to avoid the initial interference from the prompt fluorescence. Based on the I_{max}, the relative emission proportion of prompt fluorescence (PF) and TADF components (φ_{PF} and φ_{DF}) can be calculated, and see Supplementary Note 2 and Table 5 for more details.

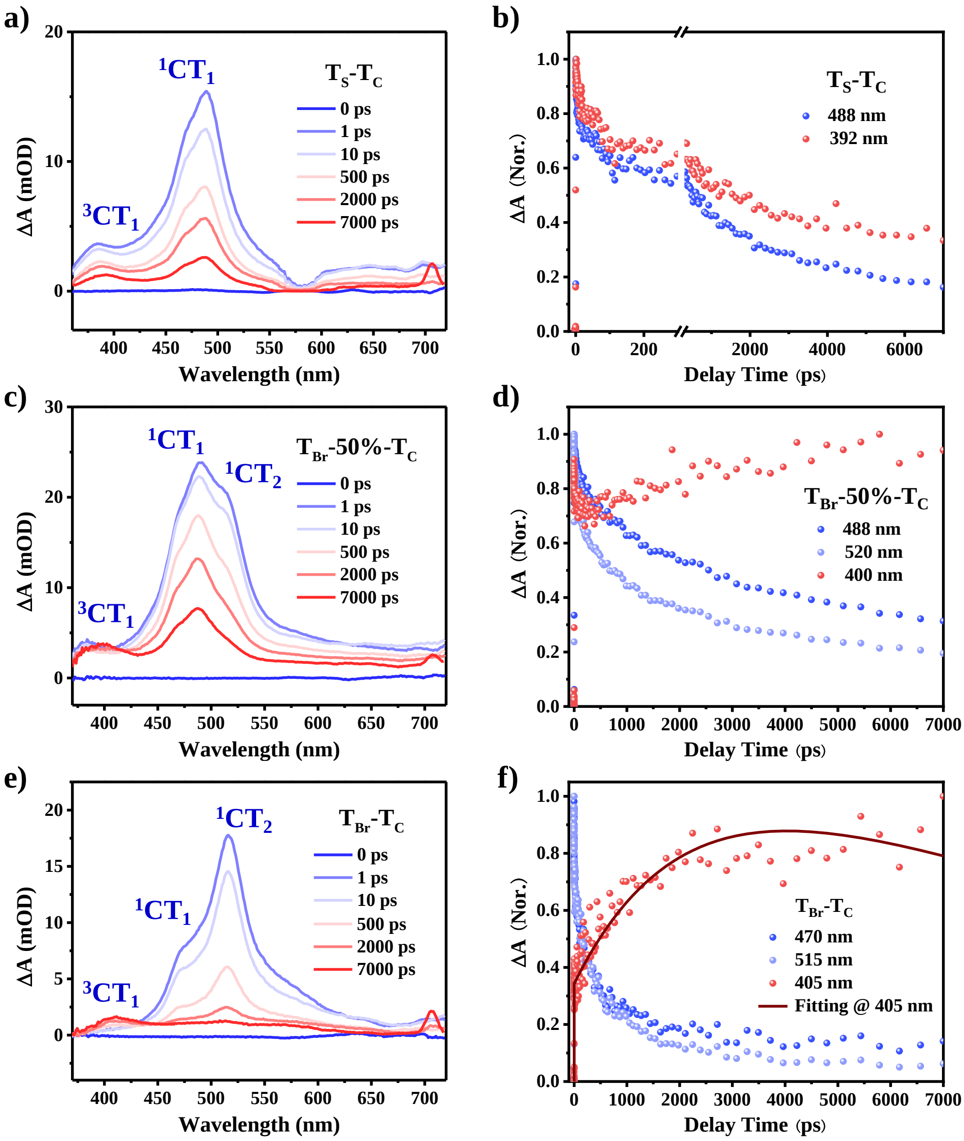

Supplementary Fig. 18 TA spectra of (a) T_S-T_C, (c) T_{Br}-50%-T_C and (e) T_{Br}-T_C at indicated delay times under the 350 nm excitation. Comparison of normalized TA kinetics probed at indicated wavelengths in (b) T_S-T_C, (d) T_{Br}-50%-T_C and (f) T_{Br}-T_C. The gradually prominent rising kinetics at ~400 nm as the T_{Br} content increases reflects the singlet-to-triplet ISC process of CT_1 excitons. The solid line in panel f is a multi-exponential fit to the rising kinetics with its fitting parameters listed in Supplementary Table 6.

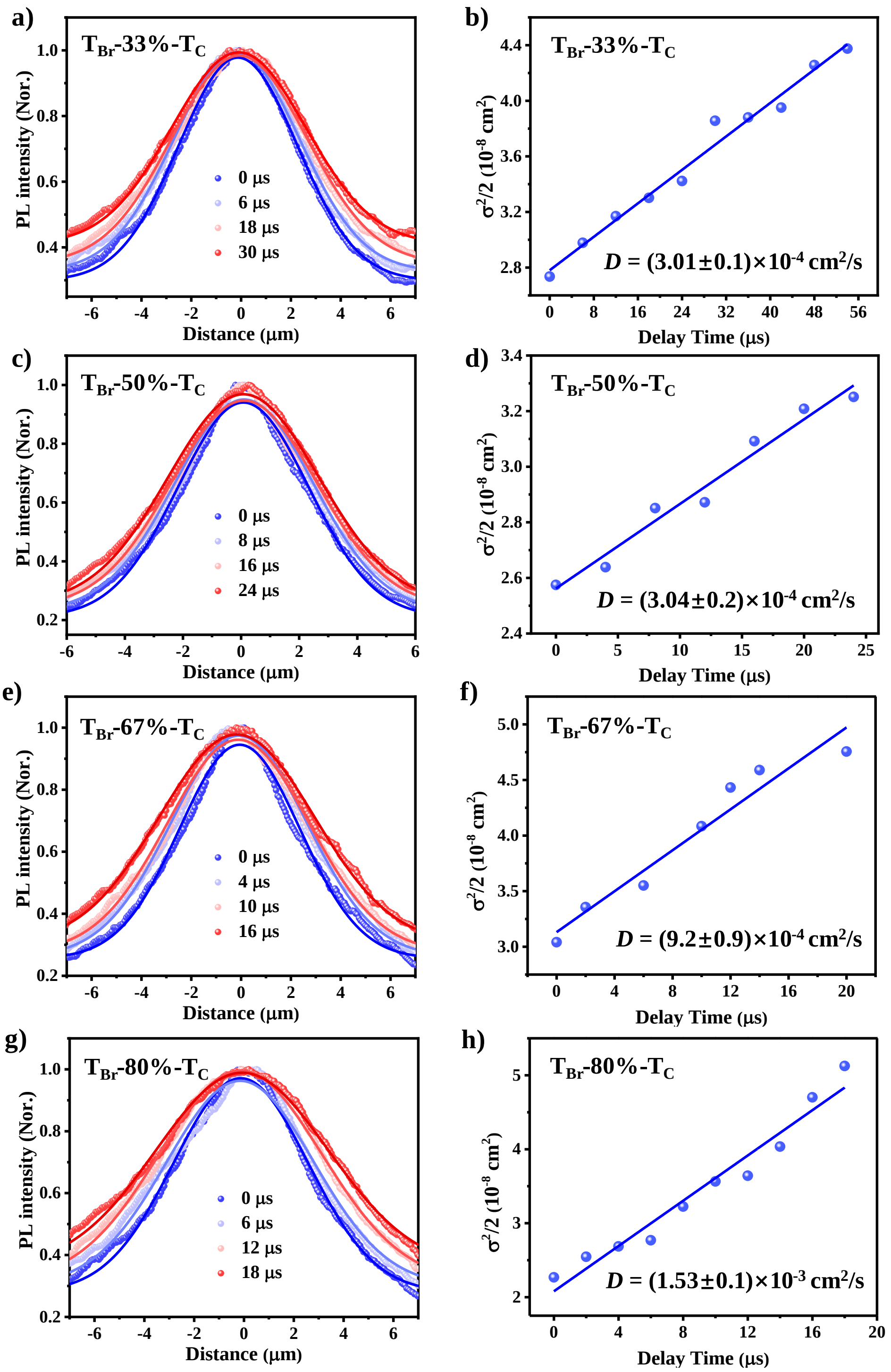

Supplementary Fig. 19 TADF imaging of CT_1 exciton transport along the direction of maximum migration efficiency in T_{Br}-x-T_C cocrystals. Normalized time-dependent 1D TADF intensity profiles of (a) T_{Br}-33%-T_C, (c) T_{Br}-50%-T_C, (e) T_{Br}-67%-T_C and (g) T_{Br}-80%-T_C collected at 670-750 nm under the 375 nm excitation, along with their Gaussian fittings. The determination of CT_1 exciton diffusion coefficient in (b) T_{Br}-

33%-T_C, (d) T_{Br}-50%-T_C, (f) T_{Br}-67%-T_C and (h) T_{Br}-80%-T_C by the linearly fitting of 1D time-dependent Gaussian variances ($\sigma^2(t)$). A significant increase in the D value can be clearly observed when T_{Br} content exceeds ~50%; see Fig. 5g and Supplementary Table 7 for more details.

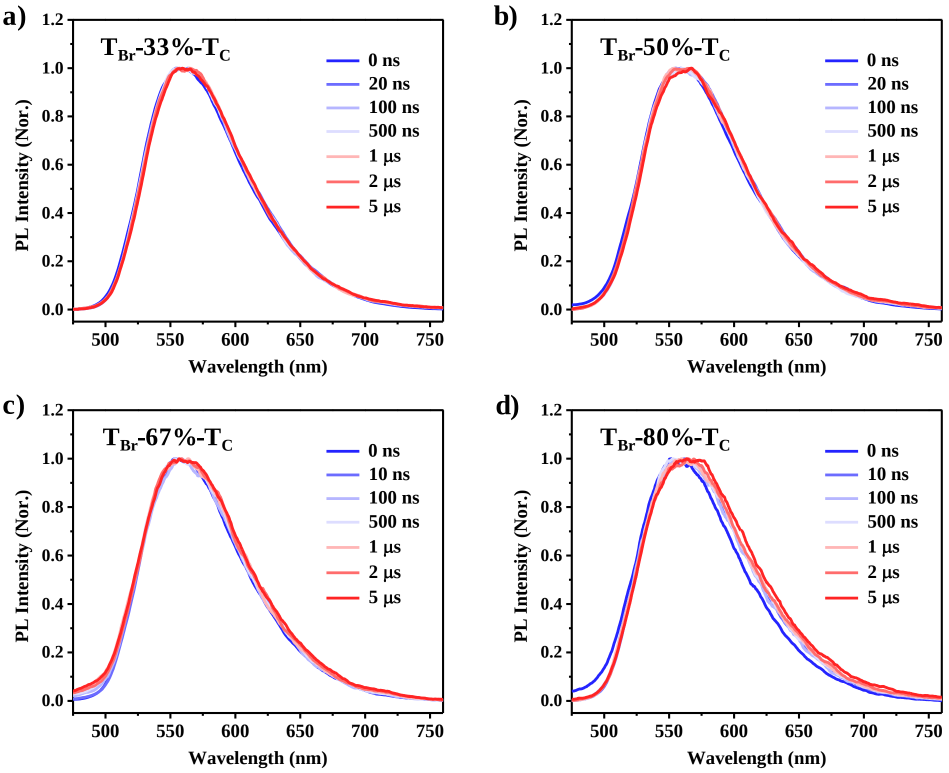

Supplementary Fig. 20 Normalized PL spectra of (a) T_{Br}-33%-T_C, (b) T_{Br}-50%-T_C, (c) T_{Br}-67%-T_C and (d) T_{Br}-80%-T_C at different delay times measured by ICMOS camera under the 343 nm excitation. An obvious spectral shift is observed when the T_{Br} content exceeds ~50%, indicating the emergence of CT_2 state in T_{Br}-x-T_C cocrystals with more than ~50% T_{Br} content.

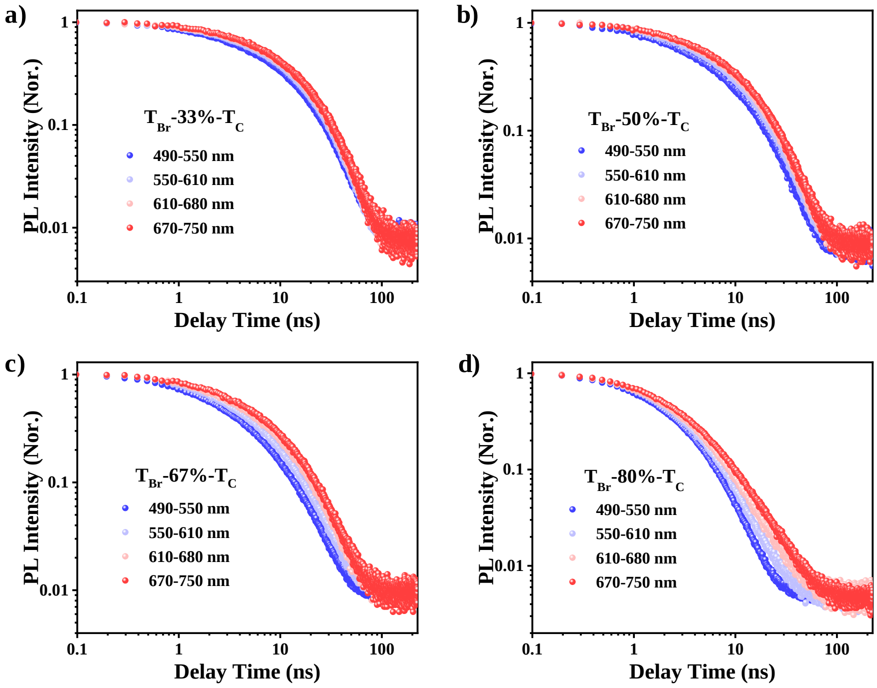

Supplementary Fig. 21 Comparison of ns-scale PL kinetics of (a) T_{Br}-33%-T_C, (b) T_{Br}-50%-T_C, (c) T_{Br}-67%-T_C and (d) T_{Br}-80%-T_C collected at different wavelength ranges under the 375 nm excitation. The PL kinetics of T_{Br}-x-T_C begin to show wavelength-dependent behavior when the T_{Br} content exceeds 50%.

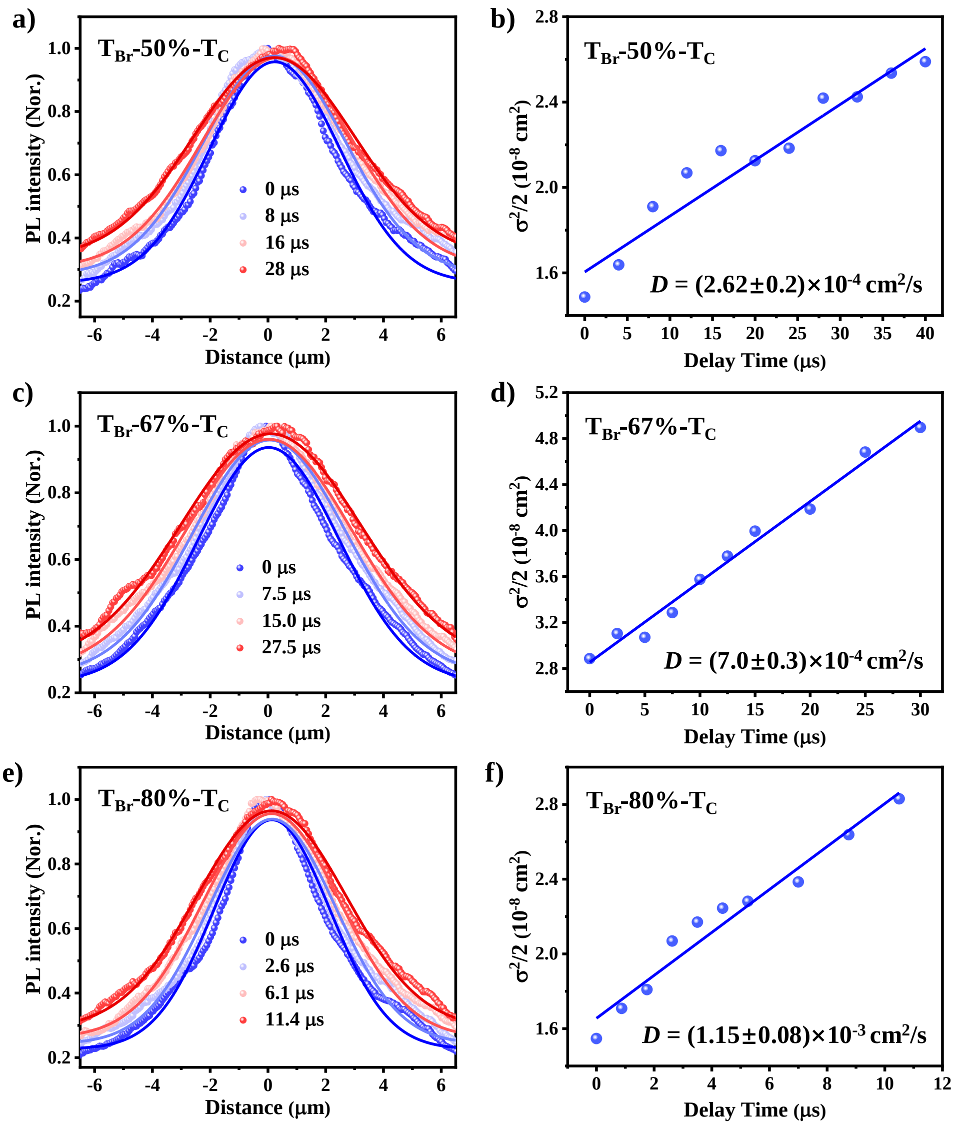

Supplementary Fig. 22 TADF imaging of the CT_2 exciton transport along the direction with maximum migration efficiency in T_{Br}-x-T_C cocrystals. Normalized time-dependent 1D TADF intensity profiles of (a) T_{Br}-50%-T_C, (c) T_{Br}-67%-T_C and (e) T_{Br}-80%-T_C collected at 490-550 nm under the 375 nm excitation, along with their Gaussian fittings. The determination of CT_2 diffusivity in (b) T_{Br}-50%-T_C, (d) T_{Br}-67%-T_C and (f) T_{Br}-80%-T_C by the linearly fitting of 1D time-dependent Gaussian variances ($\sigma^2(t)$).

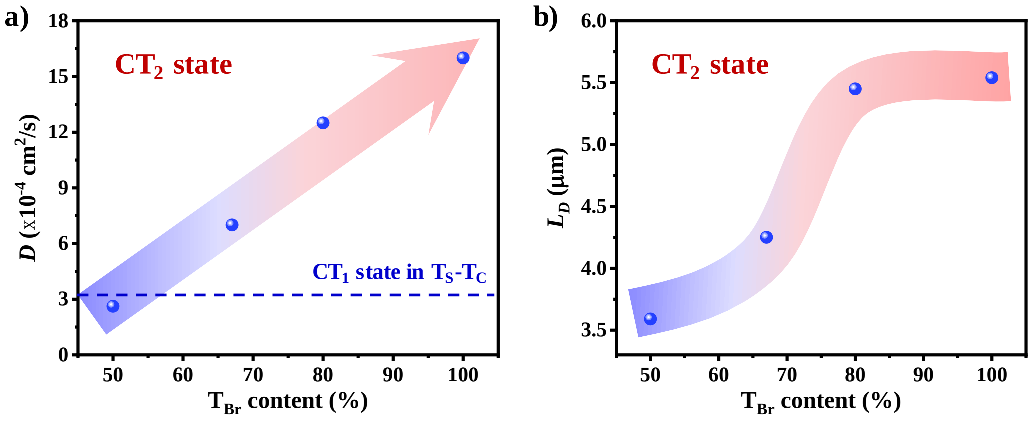

Supplementary Fig. 23 The variation trends of (a) diffusion coefficient and (b) diffusion distance of the triplet CT_2 state in T_{Br}-x-T_C series with T_{Br} content. The $^3CT_2$ diffusivity increases progressively with increasing T_{Br} content, reaching a maximum of ~1.6×10^{-3} cm^2/s in T_{Br}-T_C. The L_D of $^3CT_2$ excitons, calculated according to the fitting results of $^3CT_2$ kinetics listed in Supplementary Table 8, shows a maximum of ~5.5 μm when T_{Br} content exceeds 80%.

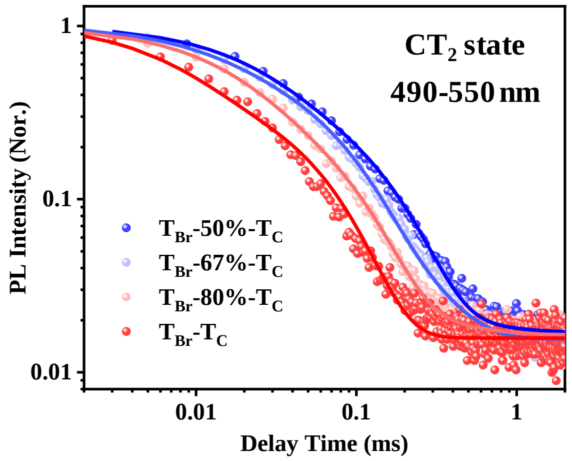

Supplementary Fig. 24 Millisecond-scale TADF kinetics of the CT_2 state in T_{Br}-x-T_C series collected at 490-550 nm. Solid lines are the multi-exponential fittings to these kinetics with fitting parameters listed in Supplementary Table 8.

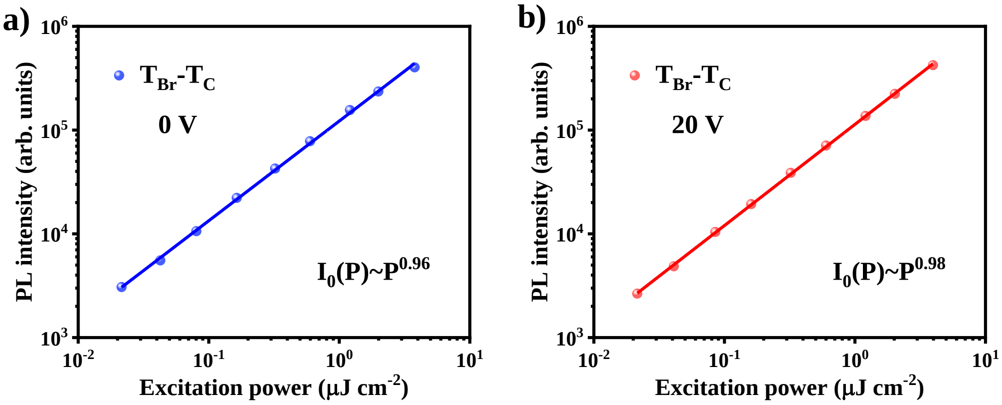

Supplementary Fig. 25 Initial maximum PL intensity (I_0, at delay time t ≈ 0 ps) as a function of excitation intensity (P) for T_{Br}-T_C measured at (a) 0 V and (b) 20 V bias voltage. Both plots follow the law of $I_0 \propto P^{\alpha}$ (solid lines), where the α values are determined to be ~1, indicating that CT excitons dominated in T_{Br}-T_C at both 0 V and 20 V.

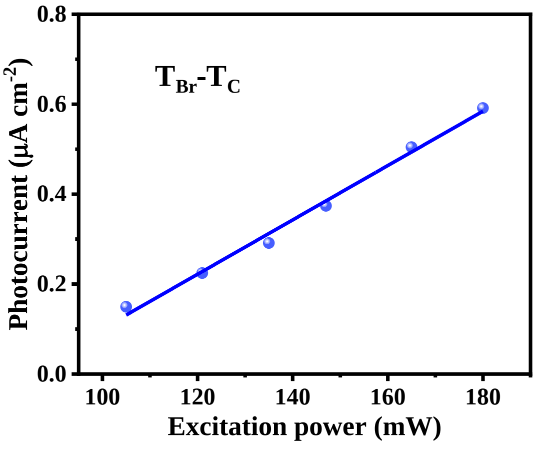

Supplementary Fig. 26 Excitation-intensity dependent photocurrent density of T_{Br}-T_C with electrode spacing of 10 μm. A clear linear relationship was established, indicating good electrode contact and efficient charge extraction at the electrode interface.

Supplementary Table 1. Crystallographic data for T_{Br}-T_C measured at 100 K.

Compound	T_{Br}-T_C
Formula	C34 H15 Br N8
Fw	615.44
Crystal system	Monoclinic
Space group	*Cc*
Temp., K	100
a, Å	26.8159(5)
b, Å	6.81290(10)
c, Å	15.5824(3)
α, deg	90
β, deg	90.063(2)
γ, deg	90
V, $Å^3$	2846.81(9)
Z	4
Dc, g/ cm^3	1.436
μ,mm^{-1}	2.283
reflns collected	30818
2θ range, deg	6.592-151.5
F(000)	1240.0
GOF on F^2	1.026
R_1/ wR_2 (I>2 (I))	R_1 = 0.0686, wR_2 = 0.1773
R_1/ wR_2 (all data)	R_1 = 0.0700, wR_2 = 0.1798

Supplementary Table 2. Selected electronic excitation energies (eV) and oscillator strengths (f), configurations of the low-lying singlet and triplet excited states of T_{Br}-T_{C}, based on the optimized ground state geometries.

Excited State	1:	Triplet-A	1.8749 eV	661.28 nm	f=0.0000	<S**2>=2.000
141 ->142		0.66926				
141 ->143		-0.22089				
Excited State	2:	Singlet-A	1.8850 eV	657.74 nm	f=0.0041	<S**2>=0.000
141 ->142		0.66926				
141 ->143		-0.22311				
Excited State	3:	Triplet-A	1.9688 eV	629.76 nm	f=0.0000	<S**2>=2.000
141 ->142		0.22149				
141 ->143		0.66909				
Excited State	4:	Singlet-A	1.9704 eV	629.23 nm	f=0.0003	<S**2>=0.000
141 ->142		0.22303				
141 ->143		0.66895				
Excited State	5:	Triplet-A	2.2867 eV	542.20 nm	f=0.0000	<S**2>=2.000
139 ->154		-0.10213				
141 ->144		0.15983				
141 ->146		0.65808				
141 <-146		0.10325				
Excited State	6:	Triplet-A	2.6938 eV	460.26 nm	f=0.0000	<S**2>=2.000
132 ->144		0.13235				
133 ->144		0.14561				
134 ->142		0.15212				
135 ->142		0.54281				
135 ->143		-0.20819				
140 ->142		-0.22440				
Excited State	7:	Triplet-A	2.7083 eV	457.79 nm	f=0.0000	<S**2>=2.000
132 ->145		0.12851				
133 ->144		-0.10061				
133 ->145		-0.15641				
134 ->143		0.11812				
136 ->142		0.23061				
136 ->143		0.58548				
Excited State	8:	Singlet-A	2.7706 eV	447.49 nm	f=0.0090	<S**2>=0.000
141 ->144		0.66625				

141 ->145	-0.23065				
Excited State 9:	Triplet-A	2.7809 eV	445.85 nm	f=0.0000	<S**2>=2.000
141 ->144	0.64079				
141 ->145	-0.23308				
141 ->146	-0.16885				
Excited State 10:	Triplet-A	2.8872 eV	429.43 nm	f=0.0000	<S**2>=2.000
141 ->144	0.24121				
141 ->145	0.66056				
Excited State 11:	Singlet-A	2.8929 eV	428.58 nm	f=0.0034	<S**2>=0.000
141 ->144	0.23075				
141 ->145	0.66576				
Excited State 12:	Singlet-A	3.0321 eV	408.91 nm	f=0.0119	<S**2>=0.000
140 ->142	0.65880				
140 ->143	-0.24372				
Excited State 13:	Triplet-A	3.0344 eV	408.59 nm	f=0.0000	<S**2>=2.000
135 ->142	0.19260				
140 ->142	0.61991				
140 ->143	-0.22890				
Excited State 14:	Triplet-A	3.2591 eV	380.43 nm	f=0.0000	<S**2>=2.000
133 ->143	0.11664				
134 ->143	0.10520				
139 ->142	0.32499				
139 ->143	0.54856				
140 ->143	-0.17979				
Excited State 15:	Singlet-A	3.2831 eV	377.64 nm	f=0.0016	<S**2>=0.000
139 ->142	0.40719				
139 ->143	0.51994				
140 ->142	-0.10004				
140 ->143	-0.21558				
Excited State 16:	Triplet-A	3.2867 eV	377.23 nm	f=0.0000	<S**2>=2.000
134 ->142	-0.12136				
139 ->142	0.59083				
139 ->143	-0.32665				

Supplementary Table 3. Simulated spin-orbit coupling matrix elements between the singlet and triplet CT states in T_S-T_C and T_{Br}-T_C, respectively.

Cocrystal	$\langle S_1\|H_{SO}\|T_1\rangle$ (cm^{-1})	$\langle S_2\|H_{SO}\|T_2\rangle$ (cm^{-1})
**T_S-T_C**	0.007	/
**T_{Br}-T_C**	0.011	0.103

Supplementary Table 4. Fitting parameters of the prompt fluorescence (PF) kinetics and TADF kinetics of the CT_1 state in T_S-T_C and T_{Br}-x-T_C cocrystals, shown in Fig. 5c and 5d, respectively.

Cocrystal	PF				TADF		
	τ_1 (a_1) (ns)	τ_2 (a_2) (ns)	τ_3 (a_3) (ns)	τ_{ave} (ns)	τ_1 (a_1) (μs)	τ_2 (a_2) (μs)	τ_3 (a_3) (μs)
**T_S-T_C**	/	/	29.16 (100%)	29.16	/	93.80 (71.5%)	758.88 (28.5%)
**T_{Br}-33%-T_C**	/	4.75 (26.1%)	16.76 (73.9%)	13.63	26.40 (25.9%)	116.47 (60.5%)	457.19 (13.6%)
**T_{Br}-50%-T_C**	/	3.70 (26.1%)	14.07 (61.3%)	9.59	21.59 (34.0%)	107.34 (58.9%)	484.81 (7.1%)
**T_{Br}-67%-T_C**	/	3.16 (45.9%)	12.65 (54.1%)	8.29	11.81 (19.5%)	58.80 (51.7%)	196.35 (28.8%)
**T_{Br}-80%-T_C**	1.47 (46.7%)	4.36 (44.9%)	16.28 (8.4%)	4.01	14.69 (34.1%)	64.70 (59.5%)	322.45 (6.4%)
**T_{Br}-T_C**	0.97 (64.4%)	3.31 (26.3%)	15.06 (9.3%)	2.90	6.68 (14.4%)	47.85 (66.7%)	223.56 (18.9%)

Note: The kinetics are fitted by a multi-exponential function, $y=\sum_i a_i \exp(-t/\tau_i)$. The average lifetimes of PF emission are calculated using the formular $\tau_{ave}=\sum_i a_i \tau_i$. The multi-exponential TADF kinetics of the CT_1 state in T_S-T_C and T_{Br}-x-T_C cocrystals originate from the diffusion of CT_1 excitons and the spectral overlap between the CT_1 and CT_2 states (corresponding to τ_1 and τ_2), and the long-lived component τ_3 represents the intrinsic lifetime of the $^3CT_1$ state.

Supplementary Table 5. Photophysical parameters of CT_1 states in T_S-T_C and T_{Br}-x-T_C cocrystals, as shown in Fig. 5e and 5f, respectively.

Cocrystal	I_{max}/I_0	η	φ_{DF}	φ_{PF}	k_{PF} (10^7 s^{-1})	k_{DF} (10^3 s^{-1})	k_1 (10^7 s^{-1})	k_2 (10^3 s^{-1})
T_S-T_C	0.092	4.85×10^{-5}	0.560	0.440	3.44	1.32	3.44	1.68
T_{Br}-33%-T_C	0.092	8.05×10^{-5}	0.730	0.270	7.34	2.19	7.34	5.92
T_{Br}-50%-T_C	0.102	8.42×10^{-5}	0.810	0.190	10.43	2.06	10.43	8.78
T_{Br}-67%-T_C	0.137	2.79×10^{-4}	0.869	0.131	12.06	5.09	12.06	33.76
T_{Br}-80%-T_C	0.142	1.76×10^{-4}	0.934	0.066	24.94	3.10	24.90	43.87
T_{Br}-T_C	0.180	3.22×10^{-4}	0.961	0.039	34.48	4.47	34.48	110.15

Note: The average lifetime of PF (τ_{ave}) and the intrinsic $^3CT_1$ lifetime τ_3 (see Supplementary Table 4) were used for the calculation of photophysical parameters. The ratio of I_{max}/I_0 was obtained from the experimentally measured ms-scale PL kinetics shown in Supplementary Fig. 17. The η, φ_{PF} and φ_{DF} were calculated through Supplementary Equation 10, 11 and 12 (see Supplementary Note 2 for details), and the rate constants of ISC and RISC (k_1 and k_2) were obtained from Supplementary Equation 16 and 21 (see Supplementary Note 3 for details).

Supplementary Table 6. Fitting parameters of fs-TA kinetics of T_{Br}- T_C probed at 400 nm shown in Supplementary Fig. 18f.

Cocrystal	τ_1 (a_1) (ps)	τ_2 (a_1) (ps)	τ_3 (a_2) (ns)	k_{ISC} (10^8 s^{-1})
T_{Br}-T_C	0.454 (-60.5%)	2430 (-39.5%)	> 10 (100%)	4.12

Note: The kinetic curve was fitted by a multi-exponential function, $y=\sum_i a_i \exp(-t/\tau_i)$. The fast-rising component originates from the direct excitation of T_{Br}- T_C, and the slow-rising component is attributed to the ISC process of CT_1 excitons in T_{Br}-T_C. Accordingly, the ISC rate constant of CT_1 state was calculated by $k_{ISC} = 1/\tau_2$.

Supplementary Table 7. Exciton transport parameters of triplet CT_1 and CT_2 states in T_S-T_C and T_{Br}-x-T_C cocrystals, as shown in Fig. 5g, 5h and Supplementary Fig. 23, respectively.

Cocrystal	CT_1 state			CT_2 state		
	D (cm^2s^{-1})	τ (μs)	L_D (μm)	D (cm^2s^{-1})	τ (μs)	L_D (μm)
T_S-T_C	3.29×10^{-4}	758.88	9.99	/	/	/
T_{Br}-33%-T_C	3.15×10^{-4}	457.19	7.59	/	/	/
T_{Br}-50%-T_C	3.14×10^{-4}	484.81	7.80	2.6×10^{-4}	107.34	3.34
T_{Br}-67%-T_C	9.58×10^{-4}	196.35	8.67	7.7×10^{-4}	58.80	4.25
T_{Br}-80%-T_C	0.0018	322.45	15.24	0.0011	64.70	5.45
T_{Br}-T_C	0.003	223.56	16.40	0.0016	47.85	5.54

Note: The intrinsic lifetimes of the $^3CT_1$ (τ_3 in Supplementary Table 4) and $^3CT_2$ states (τ_2 in Supplementary Table 8) were used for the calculation, and the L_D was determined by $L_D = 2\sqrt{D\tau}$.

Supplementary Table 8. Fitting parameters for the TADF kinetics of CT_2 state in T_{Br}-x-T_C cocrystals, as shown in Supplementary Fig. 24.

Cocrystal	TADF (CT_2 state)		
	τ_1 (a_1) (μs)	τ_2 (a_2) (μs)	τ_3 (a_3) (μs)
T_{Br}-50%-T_C	21.59 (54.6%)	107.34 (44.7%)	484.81 (0.7%)
T_{Br}-67%-T_C	11.81 (35.7%)	58.80 (57.0%)	196.35 (7.3%)
T_{Br}-80%-T_C	14.69 (57.4%)	64.70 (41.7%)	322.45 (0.9%)
T_{Br}-T_C	6.68 (58.3%)	47.85 (41.7%)	/

Note: The multi-exponential TADF kinetics of the CT_2 state in T_{Br}-x-T_C originate from the diffusion of CT_2 excitons and the spectral overlap between the CT_1 and CT_2 states

(corresponding to τ_1 and τ_3, respectively), and the component τ_2 is attributed to the intrinsic lifetime of the $^3CT_2$ state.

Supplementary References

1 Baggaley, E. *et al.* Long-lived metal complexes open up microsecond lifetime imaging microscopy under multiphoton excitation: from FLIM to PLIM and beyond. *Chem. Sci.* **5**, 879-886 (2014).

2 Xiao, Y. *et al.* Observation of triplet-assisted long-distance charge-transfer exciton transport in single organic cocrystal. *Nat. Commun.* **16**, 8081 (2025).
