# Supplementary material for: Heavy-atom functionalization promotes triplet-assisted charge-transfer exciton transport in organic cocrystals
Source: Nat Commun. 2026 Apr 22;17:5533. doi: 10.1038/s41467-026-72317-8 (PMC13287730; doi:10.1038/s41467-026-72317-8)
Supplement: Supplementary file 2 — Source Data [file 41467_2026_72317_MOESM2_ESM.pdf]

nature portfolio

Peer Review File

Heavy-atom functionalization promotes triplet-assisted charge-transfer exciton transport in organic cocrystals

Corresponding Author: Professor Wenming Tian

This file contains all reviewer reports in order by version, followed by all author rebuttals in order by version.

Version 0:

Reviewer comments:

Reviewer #1

(Remarks to the Author)
Basically, it is a simple derivative work of their recent paper about triplet-assisted long-distance charge-transfer exciton transport in single organic cocrystals (Nat Commun 16, 8081 (2025)). The main new thing in this work is that the authors introduced the heavy atom Br in the previously reported cocrystal system. However, it is well known that the heavy atom effect is able to boost the spin flip (ISC and RISC) in TADF systems. In my opinion, the results, such as an enhanced exciton diffusivity, are easily expected and cannot afford readers enough new insights. Thus, I doubt the importance of this work is enough to be published in Nature Communications. Perhaps, it is more suitable for some more specialized journals.
In addition, there are some issues:
1. Checkcif and .cif files of their crystals should be provided as well as the CCDC numbers.
2. Only decorating Br on one phenyl ring of TSB makes the cocrystal show a large crystalline cell change comparing the Ts-Tc one. I strongly suggest the authors employ di-Br-functionalized TSB (E)-1,2-bis(4-bromophenyl)ethene as the Donor 2 rather than the currently employed trans-4-Bromostilbene.
3. Why can 1CT2 and 1CT1 states both generate TADF emission? In Figure 2d, is there sufficient internal conversion between these 1CT states?
4. I didn’t find an in-depth theoretical calculation of the cocrystals. It is very important to carry out the calculations, such as the SOC strength, to support their claims.
5. I didn’t find the total PLQY information of their samples. This is essential to determine the kinetic parameters.

Reviewer #2

(Remarks to the Author)
In this manuscript, the author promoted CT exciton transport in thermally activated delayed fluorescence (TADF) cocrystals through bromine-atom functionalization. The diffusivity of triplet CT excitons in TSB-Br-containing cocrystals can be enhanced by an order of magnitude, enabling a long-distance triplet-assisted CT transport exceeding 16 μm. The CT transport and TADF-related kinetics can be effectively modulated by adjusting the TSB-Br content in the cocrystals, which enhances the utilization of CT excitons and the photocurrent responses. This research is meaningful, and I would like to recommend its publication after minor revisions.
1. The observation of triplet-assisted long-distance charge-transfer exciton transport in single organic cocrystal and the concept in Scheme 1 have been reported by the authors recently (Nat. Commun. 2025, 16, 8081), which greatly reduces the merit of the study.
2. As discussed by the authors in the introduction, “Heavy atom effect (HAE) is a well-established strategy for device optimization, as it significantly enhances the SOC interaction in organic materials.23-26 Heavy atoms (e.g., Br, I)… typically exhibit stronger atomic SOC.24,26 The enhanced SOC effectively promotes both ISC and RISC processes, thereby enhancing the efficiency of TADF emission.24,27,28 Moreover, incorporation of heavy atoms often strengthens intermolecular interactions and increases electron delocalization through improved orbital overlap, which collectively facilitates carrier transport.29,30” Therefore, HAE strategy should be a widely used method to simultaneously enhance TADF-related kinetics and CT exciton transport.
3. Although the authors provide evidence that heavy atom functionalization can promote CT transport, only one example (TSB-Br-containing cocrystals) was provided. The authors should provide two more examples to illustrate the effectiveness of this strategy.
4. In Figure 3, the authors state that “With delay time increasing, the TADF spot of both CT states expands into a discernible

elliptic pattern, indicating the anisotropic CT migration in TBr-TC (Fig. 3a and 3b).” However, the elliptic pattern is highly difficult to discern, and these patterns look more like a circle.
5. Some related references might be included in the revised manuscript: 10.1002/anie.202416181; 10.1002/anie.202005933; 10.1002/anie.201712104
6. There are some spelling and grammar errors in the main text. Please check and correct them. For example, page 4 in “These limitations substantially diminish the actual utilization of CT exctons despite their potential for long-distance migration”, “exctons” should be “excitons”.

Reviewer #3

(Remarks to the Author)
The manuscript titled “Heavy atom functionalization promotes triplet-assisted charge-transfer exciton transport in organic cocrystals” by Xiao et al. investigated a strategy for enhancing the diffusivity of charge-transfer (CT) excitons in organic cocrystals. Through the heavy atom effect, the authors observed promoted ISC and RISC kinetics, as well as a long CT migration distance exceeding 16 μm. These compelling results demonstrate that HAE can serve as an effective approach to promote CT transport in organic materials. Overall, the findings of the current manuscript are interesting, the experiments are robust, and the conclusions are well supported by experimental results. I therefore would like to recommend that the paper be accepted if the following minor points could be well addressed.
Q1: In the theoretical calculations of energy levels in TBr-TC, the CT2 and CT1 states are identified. However, the calculated energy difference between these two states is smaller than that observed experimentally, and the PL spectra of TS-TC and TBr-TC appear to be similar, could the authors elucidate the underlying reasons for these discrepancies?
Q2: The authors claim that the Br content directly affects the CT transport dynamics. When the Br content is lower than 50%, is the CT2 state completely absent, or is its signal simply too weak to be detected?
Q3: Have the authors explored substitution with other heavy atoms (for example, iodine), and if so, does it lead to similar effects on charge-transfer exciton transport?
Q4: In the text, the authors attributed the enhancement of photocurrent to the increased migration distance of CT excitons. However, photocurrent can be influenced by many other factors, such as exciton dissociation, charge extraction, electrode contact. It would be helpful if the authors could provide additional experimental or computational evidence to clarify whether enhanced exciton migration is the dominant factor underlying the observed photocurrent improvement.
Q5: The authors mentioned that structural asymmetry affects charge transport, do the authors consider that the introduction of heavy atoms may modify structural asymmetry, for example by altering crystal packing, molecular orientation, or π-π interactions? If so, could such structural changes also contribute to the observed CT exciton transport behavior?

Version 1:

Reviewer comments:

Reviewer #1

(Remarks to the Author)
The authors have adequately addressed my concerns. I recommend this work for publication.

Reviewer #2

(Remarks to the Author)
it can be accepted now

Reviewer #3

(Remarks to the Author)
I think the authors have made quite some efforts to revise the manuscript.

(1) When looking at other reviewers’ comments (Q4 of Reviewer 3), I feel the section trying to use photocurrent to justify the exciton transport requires significant addition justifications (I doubt PL measurement gives direct evidence that the exciton are not dissociated and are responsible for the photocurrent. Also the attenuation of photocurrent as a function of channel length requires a much more detailed investigation for the claim the authors intend to make) and would like suggest the authors to remove the section.

(2) The authors should change the title to better connect with the fundamental physics/chemistry that enables the better transport

Version 2:

Reviewer comments:

Reviewer #3

(Remarks to the Author)
The authors should bear in mind that it is not always a desirable thing if the excitons do not dissociate in an optoelectronic devices, such as a photodetector. The device shown essentially is governed by diffusion rather than drift of charge carriers. As the authors verify already in their rebuttal, had the device been operating in a drift model, there is no limit on channel length (~10um). It does support the strong bonding of excitons.

Other than the above point, I am OK with the revision.

Open Access This Peer Review File is licensed under a Creative Commons Attribution 4.0 International License, which permits use, sharing, adaptation, distribution and reproduction in any medium or format, as long as you give appropriate credit to the original author(s) and the source, provide a link to the Creative Commons license, and indicate if changes were made.
In cases where reviewers are anonymous, credit should be given to 'Anonymous Referee' and the source.
The images or other third party material in this Peer Review File are included in the article's Creative Commons license, unless indicated otherwise in a credit line to the material. If material is not included in the article's Creative Commons license and your intended use is not permitted by statutory regulation or exceeds the permitted use, you will need to obtain permission directly from the copyright holder.
To view a copy of this license, visit https://creativecommons.org/licenses/by/4.0/

Point-by-point response to manuscript NCOMMS-25-91317

Reviewer #1:

Basically, it is a simple derivative work of their recent paper about triplet-assisted long-distance charge-transfer exciton transport in single organic cocrystals (Nat Commun 16, 8081 (2025)). The main new thing in this work is that the authors introduced the heavy atom Br in the previously reported cocrystal system. However, it is well known that the heavy atom effect is able to boost the spin flip (ISC and RISC) in TADF systems. In my opinion, the results, such as an enhanced exciton diffusivity, are easily expected and cannot afford readers enough new insights. Thus, I doubt the importance of this work is enough to be published in Nature Communications. Perhaps, it is more suitable for some more specialized journals.

Response: We sincerely thank the reviewer for the constructive and insightful comments, and apologize for the ambiguous key points in the original manuscript resulting from our unclear expressions. While the heavy-atom effect is known to accelerate spin flip kinetics (ISC/RISC) in TADF systems, its effectiveness in facilitating CT exciton transport has not been directly demonstrated. So far, the HAE-induced carrier mobility enhancement has been proposed based on theoretical calculations and verified only through electrical measurements, which are insufficiently effective for characterizing exciton transport at the microscale. Therefore, direct and visualized experimental validation for HAE-enhanced exciton transport is highly necessary for the controllable kinetic modulation and rational material design. To address this issue, the novelty and significance of our present work include:

1. **Addressing two critical, previously unresolved bottlenecks.**

Our recent work demonstrated triplet-assisted extension of singlet CT transport to the micrometer scale, but identified (i) low triplet CT exciton diffusivity (~10^{-4} cm²/s) and (ii) inefficient RISC as limiting factors for practical application. The present study explicitly targets and resolves both bottlenecks by

chemical modification (Br incorporation), not merely reproducing prior observations.

2. Providing direct, quantitative, and visual evidence that HAE enhances CT exciton transport.

Using spatially resolved TADF-scanned imaging microscopy, we obtain direct visualization of CT exciton dynamics. Compared with T_S-T_C, the Br-functionalized cocrystal exhibits a *10-fold increase* in triplet CT exciton diffusivity (3.3×10^{-3} cm^2/s) and a record-long CT transport distance exceeding 16 μm (*1.6-fold extension*). These optical, spatial measurements directly probe CT exciton transport, distinct from charge carrier mobility verified through electrical tests, thereby providing the first direct experimental evidence that HAE can promote CT exciton transport over long distances.

3. Potential applications for device optimization.

We demonstrate that HAE simultaneously increases ISC (*~10-fold*) and RISC (*~66-fold*) rate constants and that systematic variation of Br content tunes CT transport dynamics. Importantly, these changes lead to a *6-fold enhancement* in photocurrent response in device measurement, directly validating practical relevance beyond spectroscopic observation.

Taken together, these advances extend beyond the simple observation that heavy atom substitution enhances ISC/RISC. Our data support a mechanistic chain in which Br-induced enhancement of SOC increases ISC and RISC rates and thereby improves triplet utilization. Moreover, the strengthened electronic coupling between adjacent sites promotes triplet-assisted (Dexter) transfer of CT excitons, which in turn yields a measurable increase in CT-exciton diffusivity and a corresponding improvement in device photocurrent. For these reasons, we believe this work provides novel and generalizable insights that merit publication in Nature Communications.

To address the reviewer's comments and improve the clarity, the following sentences have been revised and highlighted with yellow background on page 4 of the revised manuscript.

"Moreover, some theoretical studies have suggested that the incorporation of heavy atoms may facilitate the transport of charged carriers by increasing intermolecular orbital overlap.[29,30] These findings suggest that HAE could potentially improve not only TADF-related kinetics but also charge transport properties. However, whether such enhancements extend to CT exciton transport remains unclear. To date, the influence of HAE on CT exciton transport has not been systematically investigated, and direct compelling experimental evidences are still lacking."

In addition, there are some issues:

1. Checkcif and .cif files of their crystals should be provided as well as the CCDC numbers.

Response: We thank the reviewer for the kind suggestion. In the revised version, we have supplemented the CIF and checkCIF files of T_S-T_C and T_{Br}-T_C cocrystals as Supplementary Data 1-4 in the Supplementary Information. The crystallographic data of T_S-T_C and T_{Br}-T_C have been deposited in the Cambridge Crystallographic Data Centre, and the assigned CCDC numbers are 2521586 and 2521253, respectively. To address the review's comments, the following sentences have been added in *Data Availability* and highlighted with yellow background on page 19 of the revised manuscript.

"The crystallographic data of both T_S-T_C and T_{Br}-T_C for this study have been deposited in the Cambridge Crystallographic Data Centre (CCDC), and are accessible through CCDC numbers 2521586 and 2521253."

2. Only decorating Br on one phenyl ring of TSB makes the cocrystal show a large crystalline cell change comparing the Ts-Tc one. I strongly suggest the authors employ di-Br-functionalized TSB (E)-1,2-bis(4-bromophenyl)ethene as the Donor 2 rather than the currently employed trans-4-Bromostilbene.

Response: Thanks for the reviewer's insightful and constructive comments. We do agree with the reviewer that the dibromo-functionalized TSB can serve as a promising donor for cocrystal synthesis and the investigation of the HAE-

influenced CT exciton transport dynamics. Therefore, we synthesized the T_{Br2}-T_C cocrystal composed of dibromo-functionalized TSB donors and TCNB acceptors (Fig. R1a), and subsequently conducted PL measurements on it.

As shown in Fig. R1b, the PL emission of T_{Br2}-T_C is notably broadened compared to that of T_S-T_C and T_{Br}-T_C. The PL kinetics on ns timescale gradually accelerate as the Br amount increases (Fig. R1c). However, on the ms timescale, the dibromo-functionalization significantly prolongs the lifetime of the long-lived component (Fig. R1d). Therefore, we hypothesize that the long-lived emission in T_{Br2}-T_C arises from the phosphorescence from triplet CT state, which is enabled by the greatly enhanced SOC intensity, rather than the TADF emission from singlet CT state as observed in T_S-T_C and T_{Br}-T_C. The differences in emission process further lead to distinct long-distance CT transport mechanisms: a triplet-assisted mechanism in T_S-T_C (see Scheme 1), whereas a pure triplet transport in T_{Br2}-T_C. The changes in transport mechanism will inevitably complicate the investigation into the HAE-influenced CT transport dynamics. In addition, it can also be noticed that the initial phosphorescence intensity of T_{Br2}-T_C is much lower than the TADF intensities of T_S-T_C and T_{Br}-T_C (Fig. R1d), which severely hinders high-quality PL imaging on ms timescale due to the limited detection sensitivity. Therefore, although the monobromo-functionalization causes greater structural changes, both the strong TADF emission feature and the triplet-assisted long-distance CT transport process can be preserved in T_{Br}-T_C. Under the current circumstances, T_{Br}-T_C, rather than T_{Br2}-T_C, emerges as a more suitable reference for comparison with T_S-T_C to investigate how HAE affects CT exciton transport.

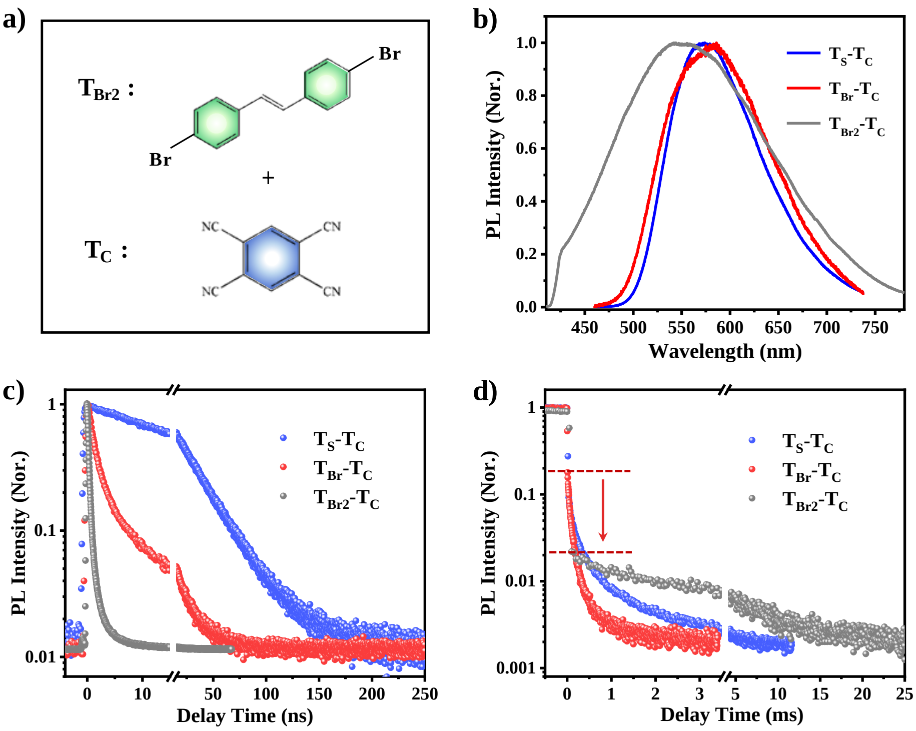

Fig. R1 (a) Chemical structures of dibromo-functionalized TSB donor (T_{Br2}) and TCNB acceptor (T_C) in T_{Br2}-T_C cocrystal. (b) PL spectra of T_S-T_C, T_{Br}-T_C and T_{Br2}-T_C under the 375 nm excitation. Comparison of (c) ns-scale and (d) ms-scale PL kinetics of T_S-T_C, T_{Br}-T_C and T_{Br2}-T_C collected at the wavelength range of 670-750 nm.

Moreover, to address the reviewer's concern regarding our investigation and verify the universality of HAE-promoted CT exciton transport, two sets of cocrystals were synthesized, and their CT transport properties were respectively compared before and after the heavy atom functionalization. Firstly, the chlorine atom was used for heavy atom functionalization instead of the bromine atom. The T_{Cl}-T_C (*trans*-4-Chlorostilbene-1,2,4,5-tetracyanobenzene) cocrystal was successfully synthesized, which exhibits notable ^{3}CT exciton transport with the diffusion coefficient determined to be ~(1.0±0.1)×10^{-3} cm^2/s (Fig. R2). This value lies between the ^{3}CT diffusion coefficients of T_S-T_C ((3.5±0.2)×10^{-4} cm^2/s) and T_{Br}-T_C ((3.3±0.3)×10^{-3} cm^2/s), indicating that HAE directly promotes CT exciton transport, and this promotion positively correlates with the relativistic mass of the introduced heavy atoms. Secondly, in the case of FR-T_C (fluorene-1,2,4,5-tetracyanobenzene) and BrFR-T_C (2-bromocarbazole-1,2,4,5-tetracyanobenzene), the PL decay kinetics on both ns and ms timescales are accelerated because of the

incorporation of Br atom (Fig. R3a-d). Moreover, the HAE-promoted CT exciton transport can be verified as an obvious broadening of the TADF intensity profiles is observed only in BrFR-T_C (Fig. R3e and R3f), and the ^{3}CT diffusion coefficient is determined to be ~$(7.3\pm0.4)\times10^{-4}$ cm^2/s (Fig. R3g). These results indicate that the promotion effect of heavy atom incorporation on CT exciton transport is universal, irrespective of the types of heavy atoms and the cocrystal systems.

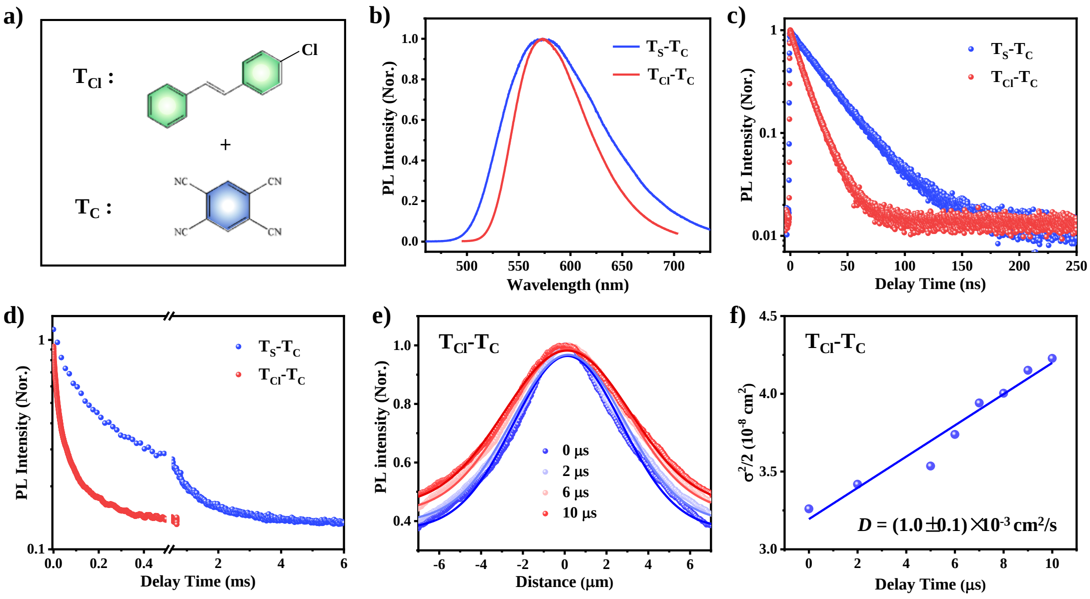

Fig. R2 (Supplementary Fig. 13) Imaging and modeling of CT exciton transport in T_{Cl}-T_C. (a) Chemical structures of T_{Cl} and T_C. Comparison of the normalized (b) PL spectra, (c) PL kinetics on ns timescale, and (d) PL kinetics on ms timescale between T_S-T_C and T_{Cl}-T_C. (e) Normalized 1D TADF intensity profiles of T_{Cl}-T_C at different delay times, along with their Gaussian fittings. (f) The determination of the ^{3}CT diffusion coefficient through linearly fitting of the 1D time-dependent Gaussian variances ($\sigma^2(t)$).

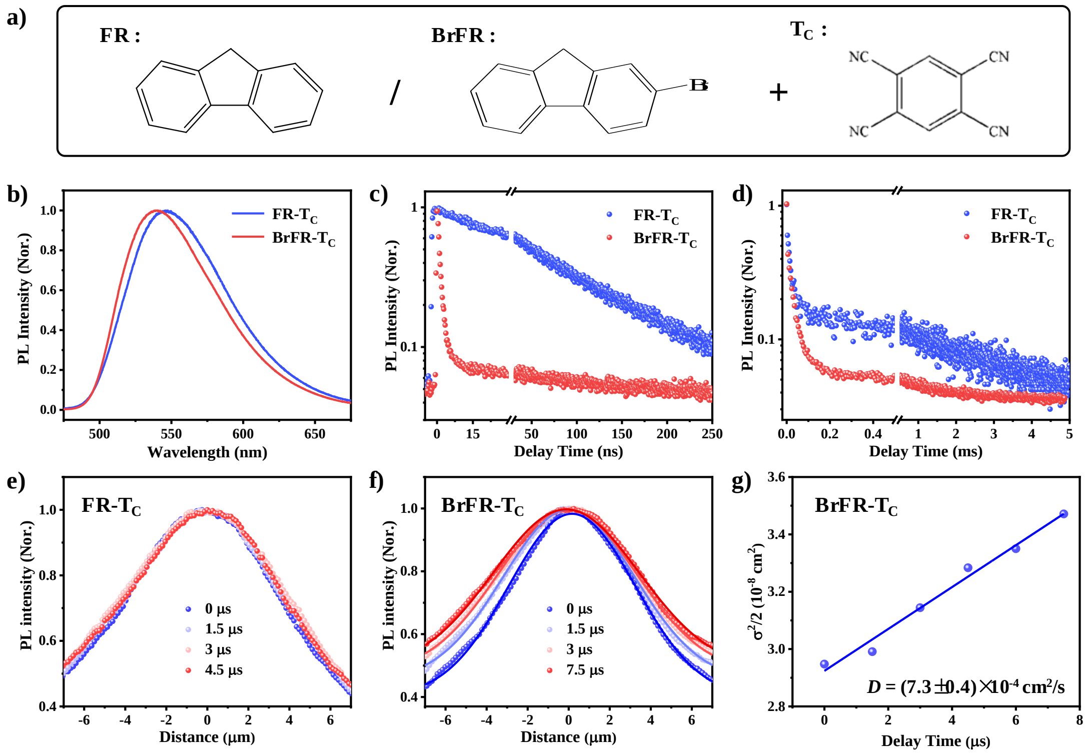

Fig. R3 (Supplementary Fig. 14) Imaging and modeling of CT exciton transport in FR-T_C and BrFR-T_C. (a) Chemical structures of FR, BrFR and T_C. Comparison of the normalized (b) PL spectra, (c) PL kinetics on ns timescale, and (d) PL kinetics on ms timescale between FR-T_C and BrFR-T_C. Normalized 1D TADF intensity profiles of (e) FR-T_C and (f) BrFR-T_C at different delay times, along with their Gaussian fittings. (f) The determination of the ^{3}CT diffusion coefficient in BrFR-T_C through linearly fitting of the 1D time-dependent Gaussian variances ($\sigma^2(t)$).

To better address the reviewer's comments, following changes have been revised in our manuscript.

(1) Fig. R2 and R3 (Supplementary Fig. 13 and 14) have been added and highlighted with yellow backgrounds on pages S13-14 of the revised SI.

(2) Following sentences have been revised in the main text, which are highlighted with yellow backgrounds on pages 11-12.

"Furthermore, we also verified the universality of HAE-promoted triplet CT exciton transport across different heavy atoms and material systems. First, the chlorine atom was employed as an alternative heavy atom instead of the bromine atom. The T_{Cl}-T_C (*trans*-4-Chlorostilbene-1,2,4,5-tetracyanobenzene) cocrystal was successfully synthesized and exhibits notable ^{3}CT exciton transport, with a diffusion coefficient of

~(1.0±0.1)×10^{-3} cm^2/s (Supplementary Fig. 13). This value lies between those of T_S-T_C ((3.5±0.2)×10^{-4} cm^2/s) and T_{Br}-T_C ((3.3±0.3)×10^{-3} cm^2/s). The systematic trend in diffusivity correlates with the increasing atomic number of the incorporated halogen (Cl<Br), consistent with progressively stronger SOC. This observation supports the conclusion that HAE directly promotes CT exciton transport. Second, we examined another donor-acceptor system, FR-T_C (fluorene-1,2,4,5-tetracyanobenzene) and its brominated analogue BrFR-T_C (2-bromocarbazole-1,2,4,5-tetracyanobenzene) cocrystals.[47] In this pair, significant broadening of the TADF intensity profiles is observed only in BrFR-T_C, with a ^{3}CT diffusion coefficient determined to be ~(7.3±0.4)×10^{-4} cm^2/s (Supplementary Fig. 14). These results directly demonstrate the universality of HAE-induced CT transport enhancement, which is irrespective of different heavy atoms and distinct cocrystal systems, indicating that the strategy is not material-specific but broadly applicable."

(3) The following reference has been added in the revision (highlighted with a yellow background on page 24).

47 Yao, W. *et al*. Synthesis and photophysical properties of charge transfer cocrystals based on TCNB and fluorene and its derivatives. *CrystEngComm* **26**, 2155-2165 (2024).

3. Why can 1CT2 and 1CT1 states both generate TADF emission? In Figure 2d, is there sufficient internal conversion between these 1CT states?

Response: Thanks for the reviewer's thoughtful comments. We fully understand the reviewer's concern regarding the potential internal conversion (IC) between the $^1CT_1$ and $^1CT_2$ states, which may impede the TADF emission from the $^1CT_2$ state if the IC rate is too fast. However, as can be seen from Fig. 1c, the CT_1 and CT_2 states in T_{Br}-T_C actually localize on different subunits—the one with Br group and the one without it. The spatial separation between these two subunits might remarkably slow down the energy transfer rate (k_{EnT}) between the singlet states of CT_1 and CT_2 (Fig. 2d), which completely differs from the IC between excited states

within a single moiety. In this case, the generation of TADF emission from the $^1CT_2$ state is entirely possible due to the competition between $^1CT_2$ emission and IC process.

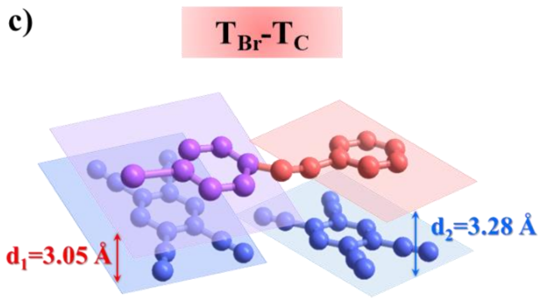

Fig. 1 (**c**) The A-D-A unit structure of T_{Br}-T_C.

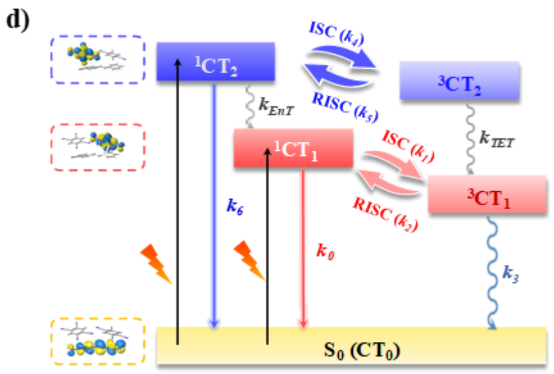

Fig. 2 (**d**) Schematic diagram for dual TADF emission mechanism in T_{Br}-T_C.

In fact, although energy transfer occurs from the CT_2 to CT_1 state, we indeed observed long-lived TADF emission from the $^1CT_2$ state of T_{Br}-T_C (see Fig. 2c and discussion on pages 8-9 in the main text). Based on the temperature-dependent PL measurements, an obvious temperature threshold was observed. Both the PL spectra intensity and the CT state lifetime increase significantly below 213 K (Supplementary Fig. 7 and 8), indicating the temperature-dependent emission transition from TADF at RT to phosphorescence at low temperatures. Therefore, we believe that the proposed dual TADF emission mechanism in T_{Br}-T_C is reasonable, and similar dual TADF models have also been reported in other studies (*Angew. Chem. Int. Ed.* 2020, 59, 17081; *ACS Appl. Mater. Interfaces* 2021, 13, 49066).

4. I didn't find an in-depth theoretical calculation of the cocrystals. It is very important to carry out the calculations, such as the SOC strength, to support their claims.

Response: We are grateful to the reviewer for the constructive comments and thoughtful suggestions. Following the reviewer's advice, we have performed a thorough theoretical calculation of the SOC matrix element between each singlet and triplet CT state in T_S-T_C and T_{Br}-T_C, respectively. As listed in Table R1 (Supplementary Table 3 in revised manuscript), it is evident that, apart from a large SOC constant (0.103 cm^{-1}) between the $^1CT_2$ and $^3CT_2$ (S_2 and T_2) states in T_{Br}-T_C, the SOC constant (0.011 cm^{-1}) between the $^1CT_1$ and $^3CT_1$ (S_1 and T_1) states also increased remarkably compared to that in T_S-T_C (0.007 cm^{-1}). These results directly verify the comprehensive improvement of SOC intensity in T_{Br}-T_C, which support our statement that the incorporation of heavy atoms can simultaneously promote ISC/RISC rates and facilitate CT exciton transport due to the enhanced SOC intensity.

To address the reviewer's comments, the following changes have been made in the revised manuscript.

(1) Table R1 (Supplementary Table 3) has been added and highlighted with yellow background on page S26 of the revised SI.

Table R1 (Supplementary Table 3) Simulated spin-orbit coupling matrix elements between the singlet and triplet CT states in T_S-T_C and T_{Br}-T_C, respectively.

Cocrystal	$\langle S_1 \| H_{SO} \| T_1 \rangle$ (cm^{-1})	$\langle S_2 \| H_{SO} \| T_2 \rangle$ (cm^{-1})
T_S-T_C	0.007	/
T_{Br}-T_C	0.011	0.103

(2) The following sentences have been added in the revision (highlighted with yellow background on page 8).

"The SOC matrix elements between the singlet and triplet CT states in T_{Br}-T_C and T_S-T_C were also calculated (Supplementary Table 3). For T_{Br}-T_C, the values of $<S_1|H_{SO}|T_1>$ and $<S_2|H_{SO}|T_2>$ were 0.011 and 0.103 cm^{-1}, respectively, both exceeding the corresponding value of $<S_1|H_{SO}|T_1>$ in T_S-T_C (0.007 cm^{-1}), directly verifying the HAE-induced SOC enhancement in T_{Br}-T_C. The enhanced SOC intensities lead to the accelerated PL kinetics for both CT_1 and CT_2 states in T_{Br}-T_C. Moreover, the distinct kinetic features of these two CT states, along with a potential CT_2-to-CT_1 energy transfer, contribute to the wavelength-dependent kinetic behavior observed in T_{Br}-T_C (Fig. 2b and 2c)."

5. I didn't find the total PLQY information of their samples. This is essential to determine the kinetic parameters.

Response: We sincerely appreciate the reviewer's constructive comments. In accordance with the reviewer's valuable suggestion, the total PLQY of T_S-T_C and T_{Br}-T_C was measured. It was found that the PLQY decreases from 12.4% (T_S-T_C) to 2.6% (T_{Br}-T_C) with the incorporation of Br atom. This reduction can be ascribed to the enhanced non-radiative recombination in T_{Br}-T_C induced by the HAE, which is commonly observed in heavy-atom-functionalized materials (*Adv. Mater.* 2017, 29, 1701987; *Angew. Chem. Int. Ed.* 2025, 64, e202502380).

PLQY is the ratio of emitted photons to absorbed photons, quantifying the total efficiency of radiative decay pathways from all excited states in a material. In our cases, it encompasses both prompt fluorescence (PF) and delayed fluorescence (DF) efficiencies, i.e., $PLQY = \emptyset_{PF} + \emptyset_{DF}$. Although the total PLQY is important, it alone is insufficient for calculating the target kinetic parameters, because the determination of rate constants of k_{ISC} (k_1) and k_{RISC} (k_2) actually requires the ratio of PF and DF efficiencies (i.e., $\frac{\emptyset_{PF}}{\emptyset_{DF}}$, see Supplementary Note 3 for details).

Herein, based on the principle of multipulse-excited TCSPC phosphorescence decay recording technique employed in this work (see Supplementary Note 2 for

details), the relative emission proportions of PF and DF components to the total PLQY (φ_{PF} and φ_{DF}) can be calculated by:

$$\varphi_{DF} = \frac{\emptyset_{DF}}{PLQY} = \frac{\eta\tau_{DF}}{\eta\tau_{DF} + (1-\eta)\tau_{PF}}$$

$$\varphi_{PF} = \frac{\emptyset_{PF}}{PLQY} = 1 - \varphi_{DF}$$

where η is the weight of TADF intensity relative to PF intensity at t = 0 under single-pulse excitation; τ_{DF} and τ_{PF} are the lifetimes of DF and PF emissions, respectively. Based on the calculated φ_{PF} and φ_{DF}, the ratio of $\frac{\emptyset_{PF}}{\emptyset_{DF}} = \frac{\varphi_{PF}}{\varphi_{DF}}$ can be obtained. Moreover, by using the experimentally obtained rate constants k_{PF} and k_{DF}, as well as their relative emission proportions (φ_{PF} and φ_{DF}), the ISC and RISC rate constants (k_1 and k_2) can be determined (Supplementary Table 5, and see Supplementary Note 3 for details).

Supplementary Table 5. Photophysical parameters of CT_1 states in T_S-T_C and T_{Br}-x-T_C cocrystals, as shown in Fig. 4e and 4f, respectively.

Cocrystal	I_{max} / I_0	η	φ_{DF}	φ_{PF}	k_{PF} (10^7 s^{-1})	k_{DF} (10^3 s^{-1})	k_1 (10^7 s^{-1})	k_2 (10^3 s^{-1})
T_S-T_C	0.092	4.85×10^{-5}	0.560	0.440	3.44	1.32	3.44	1.68
T_{Br}-33%-T_C	0.092	8.05×10^{-5}	0.730	0.270	7.34	2.19	7.34	5.92
T_{Br}-50%-T_C	0.102	8.42×10^{-5}	0.810	0.190	10.43	2.06	10.43	8.78
T_{Br}-67%-T_C	0.137	2.79×10^{-4}	0.869	0.131	12.06	5.09	12.06	33.76
T_{Br}-80%-T_C	0.142	1.76×10^{-4}	0.934	0.066	24.94	3.10	24.90	43.87
T_{Br}-T_C	0.180	3.22×10^{-4}	0.961	0.039	34.48	4.47	34.48	110.15

To enhance the clarity in expressing the relative emission proportions (φ_{PF} and φ_{DF}) and the realistic efficiencies ($\emptyset_{PF}$ and $\emptyset_{DF}$) of PF and DF components, the following changes have been made in revised main text and SI.

(1) The following sentence has been revised in the revision (highlighted with a yellow background on page 12).

"Based on the experimentally determined rate constants of prompt fluorescence (k_{PF}) and TADF (k_{DF}), as well as their relative emission proportions (φ_{PF} and φ_{DF}, see Supplementary Note 2, Supplementary Fig. 17 and Table 5 for detailed discussion), the

ISC and RISC rate constants (k_1 and k_2) can be determined (Supplementary Table 5, and see Supplementary Note 3 for more details)."

(2) The expressions for the relative emission proportions (φ_{PF} and φ_{DF}) and the realistic efficiencies ($\emptyset_{PF}$ and $\emptyset_{DF}$) of PF and DF components in Supplementary Note 2, Supplementary Note 3, Supplementary Fig. 17, and Supplementary Table 5 have been rigorously revised and are all highlighted with yellow backgrounds in revised SI.

Reviewer #2:

In this manuscript, the author promoted CT exciton transport in thermally activated delayed fluorescence (TADF) cocrystals through bromine-atom functionalization. The diffusivity of triplet CT excitons in TSB-Br-containing cocrystals can be enhanced by an order of magnitude, enabling a long-distance triplet-assisted CT transport exceeding 16 μm. The CT transport and TADF-related kinetics can be effectively modulated by adjusting the TSB-Br content in the cocrystals, which enhances the utilization of CT excitons and the photocurrent responses. This research is meaningful, and I would like to recommend its publication after minor revisions.

Response: We are deeply grateful to the reviewer for his/her thorough review and positive comments.

1. The observation of triplet-assisted long-distance charge-transfer exciton transport in single organic cocrystal and the concept in Scheme 1 have been reported by the authors recently (Nat. Commun. 2025, 16, 8081), which greatly reduces the merit of the study.

Response: We sincerely thank the reviewer for this insightful comment, and apologize for the ambiguous key points in the original manuscript resulting from our unclear expressions. Although the triplet-assisted long-distance CT exciton

transport has been observed in our previous work, this study further makes impactful advancements. The novelty and significance of our present work include:

1. Addressing two critical, previously unresolved bottlenecks.

Our recent work demonstrated triplet-assisted extension of singlet CT transport to the micrometer scale, but identified (i) low triplet CT exciton diffusivity (~10^{-4} cm^2/s) and (ii) inefficient RISC as limiting factors for practical application. The present study explicitly targets and resolves both bottlenecks by chemical modification (Br incorporation), not merely reproducing prior observations.

2. Providing direct, quantitative, and visual evidence that HAE enhances CT exciton transport.

Using spatially resolved TADF-scanned imaging microscopy, we obtain direct visualization of CT exciton dynamics. Compared with T_S-T_C, the Br-functionalized cocrystal exhibits a *10-fold increase* in triplet CT exciton diffusivity (3.3×10^{-3} cm^2/s) and a record-long CT transport distance exceeding 16 μm (*1.6-fold extension*). These optical, spatial measurements directly probe CT exciton transport, distinct from charge carrier mobility verified through electrical tests, thereby providing the first direct experimental evidence that HAE can promote CT exciton transport over long distances.

3. Potential applications for device optimization.

We demonstrate that HAE simultaneously increases ISC (*~10-fold*) and RISC (*~66-fold*) rate constants and that systematic variation of Br content tunes CT transport dynamics. Importantly, these changes lead to a *6-fold enhancement* in photocurrent response in device measurement, directly validating practical relevance beyond spectroscopic observation.

Therefore, these advances extend beyond our previous investigation on long-distance CT exciton transport. The findings in this work further offer compelling evidence that HAE can serve as an effective strategy to promote long-distance CT transport, and demonstrate its significance and potential applications for device

optimization. For these reasons, we believe this work provides novel and generalizable insights that merit publication in Nature Communications.

2. As discussed by the authors in the introduction, "Heavy atom effect (HAE) is a well-established strategy for device optimization, as it significantly enhances the SOC interaction in organic materials.23-26 Heavy atoms (e.g., Br, I)… typically exhibit stronger atomic SOC.24,26 The enhanced SOC effectively promotes both ISC and RISC processes, thereby enhancing the efficiency of TADF emission.24,27,28 Moreover, incorporation of heavy atoms often strengthens intermolecular interactions and increases electron delocalization through improved orbital overlap, which collectively facilitates carrier transport.29,30" Therefore, HAE strategy should be a widely used method to simultaneously enhance TADF-related kinetics and CT exciton transport.

Response: We sincerely thank the reviewer for the insightful comments and apologize for any confusion caused by our previous expression. While the heavy-atom effect is known to accelerate spin flip kinetics (ISC/RISC) in TADF systems, its effectiveness in facilitating CT exciton transport has not been directly demonstrated. So far, the HAE-induced carrier mobility enhancement has been proposed based on theoretical calculations and verified only through electrical measurements, which are insufficiently effective for characterizing exciton transport at the microscale. Therefore, direct and visualized experimental validation for HAE-enhanced exciton transport is highly necessary for the controllable kinetic modulation and rational material design.

In this work, we systematically investigated the influence of HAE on long-distance CT exciton transport in two TADF cocrystals (T_{Br}-T_C and T_S-T_C) by using TADF-scanned imaging microscopy. These optical, spatial measurements directly probe CT exciton transport, distinct from charge carrier mobility verified through electrical tests, thereby providing the first direct experimental evidence that HAE can promote CT exciton transport over long distances.

To address the reviewer's comments and improve the clarity, the following sentences have been revised and highlighted with yellow background on page 4 of the revised manuscript.

"Moreover, some theoretical studies have suggested that the incorporation of heavy atoms may facilitate the transport of charged carriers by increasing intermolecular orbital overlap.[29,30] These findings suggest that HAE could potentially improve not only TADF-related kinetics but also charge transport properties. However, whether such enhancements extend to CT exciton transport remains unclear. To date, the influence of HAE on CT exciton transport has not been systematically investigated, and direct compelling experimental evidences are still lacking."

3. Although the authors provide evidence that heavy atom functionalization can promote CT transport, only one example (TSB-Br-containing cocrystals) was provided. The authors should provide two more examples to illustrate the effectiveness of this strategy.

Response: We thank for the reviewer's constructive and insightful comments. To verify the universality of HAE-promoted CT exciton transport, two sets of cocrystals were synthesized, and their CT transport properties were respectively compared before and after the heavy atom functionalization. Firstly, the chlorine atom was used for heavy atom functionalization instead of the bromine atom. The T_{Cl}-T_C (*trans*-4-Chlorostilbene-1,2,4,5-tetracyanobenzene) cocrystal was successfully synthesized, which exhibits notable ^{3}CT exciton transport with the diffusion coefficient determined to be ~(1.0±0.1)×10^{-3} cm^2/s (Fig. R2). This value lies between the ^{3}CT diffusion coefficients of T_S-T_C ((3.5±0.2)×10^{-4} cm^2/s) and T_{Br}-T_C ((3.3±0.3)×10^{-3} cm^2/s), indicating that HAE directly promotes CT exciton transport, and this promotion positively correlates with the relativistic mass of the introduced heavy atoms. Secondly, in the case of FR-T_C (fluorene-1,2,4,5-tetracyanobenzene) and BrFR-T_C (2-bromocarbazole-1,2,4,5-tetracyanobenzene), the PL decay kinetics on both ns and ms timescales are accelerated because of the incorporation of Br atom (Fig. R3a-d). Moreover, the HAE-promoted CT exciton

transport can be verified as an obvious broadening of the TADF intensity profiles is observed only in BrFR-T_C (Fig. R3e and R3f), and the ^{3}CT diffusion coefficient is determined to be ~$(7.3\pm0.4)\times10^{-4}$ cm^2/s (Fig. R3g). These results indicate that the promotion effect of heavy atom incorporation on CT exciton transport is universal, irrespective of the types of heavy atoms and the material systems.**

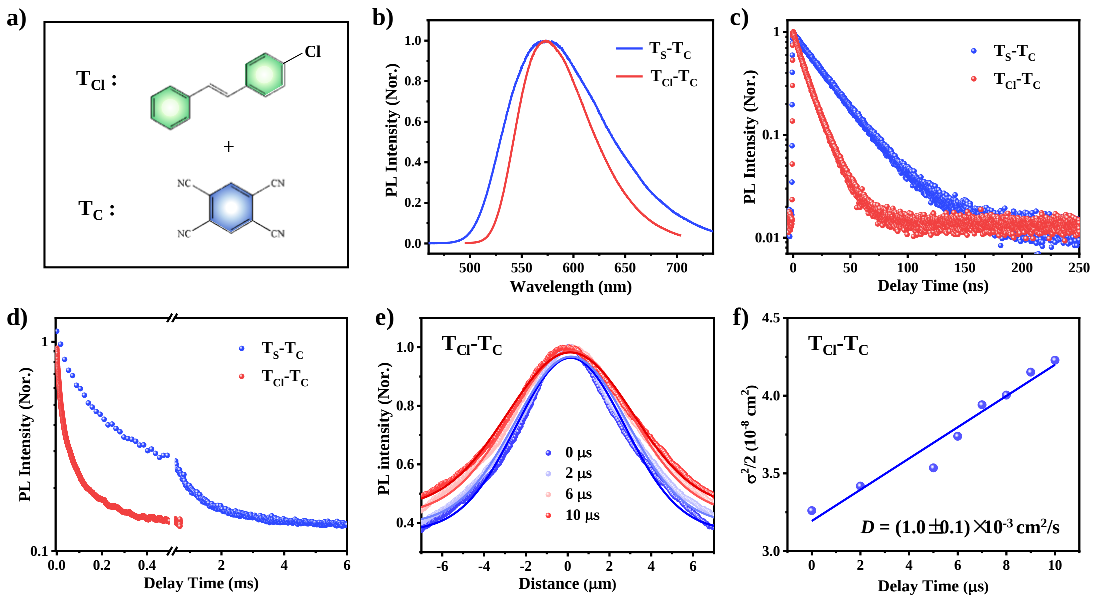

Fig. R2 (Supplementary Fig. 13) Imaging and modeling of CT exciton transport in T_{Cl}-T_C. (a) Chemical structures of T_{Cl} and T_C. Comparison of the normalized (b) PL spectra, (c) PL kinetics on ns timescale, and (d) PL kinetics on ms timescale between T_S-T_C and T_{Cl}-T_C. (e) Normalized 1D TADF intensity profiles of T_{Cl}-T_C at different delay times, along with their Gaussian fittings. (f) The determination of the ^{3}CT diffusion coefficient through linearly fitting of the 1D time-dependent Gaussian variances ($\sigma^2(t)$).

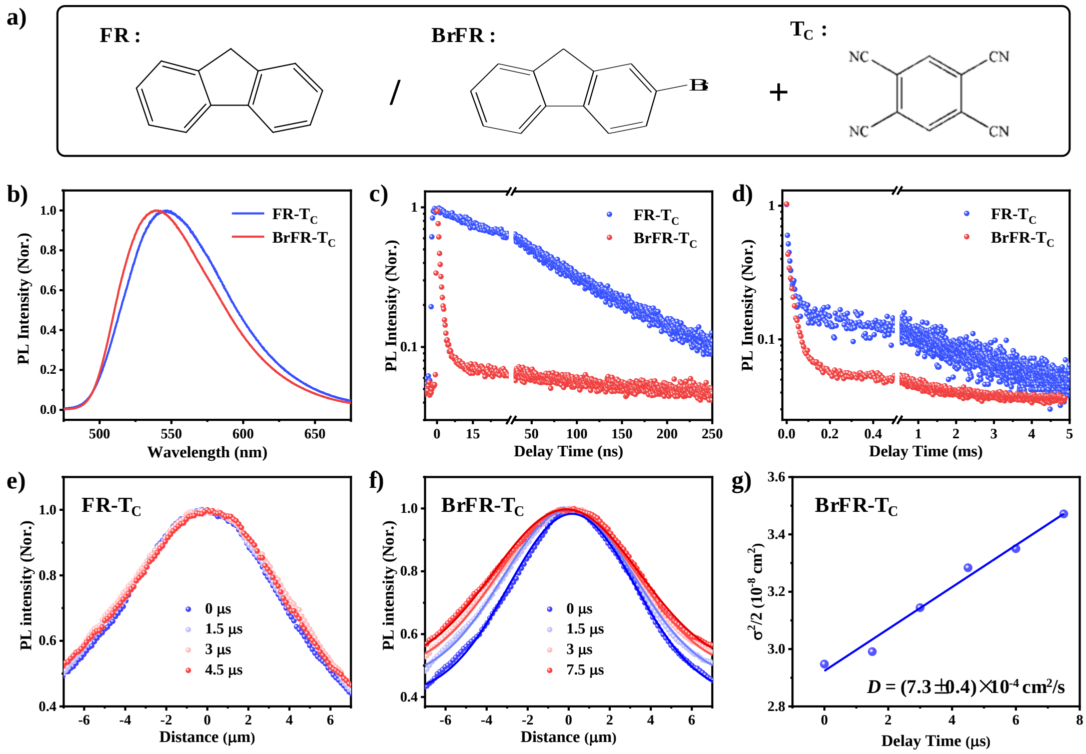

Fig. R3 (Supplementary Fig. 14) Imaging and modeling of CT exciton transport in FR-T_C and BrFR-T_C. (a) Chemical structures of FR, BrFR and T_C. Comparison of the normalized (b) PL spectra, (c) PL kinetics on ns timescale, and (d) PL kinetics on ms timescale between FR-T_C and BrFR-T_C. Normalized 1D TADF intensity profiles of (e) FR-T_C and (f) BrFR-T_C at different delay times, along with their Gaussian fittings. (f) The determination of the ^{3}CT diffusion coefficient in BrFR-T_C through linearly fitting of the 1D time-dependent Gaussian variances ($\sigma^2(t)$).

To better address the reviewer's comments, following changes have been revised in our manuscript.

(1) Fig. R2 and R3 (Supplementary Fig. 13 and 14) have been added and highlighted with yellow backgrounds on pages S13-14 of the revised SI.

(2) Following sentences have been revised in the main text, which are highlighted with yellow backgrounds on pages 11-12.

"Furthermore, we also verified the universality of HAE-promoted triplet CT exciton transport across different heavy atoms and material systems. First, the chlorine atom was employed as an alternative heavy atom instead of the bromine atom. The T_{Cl}-T_C (*trans*-4-Chlorostilbene-1,2,4,5-tetracyanobenzene) cocrystal was successfully synthesized and exhibits notable ^{3}CT exciton transport, with a diffusion coefficient of

~(1.0±0.1)×10^{-3} cm^2/s (Supplementary Fig. 13). This value lies between those of T_S-T_C ((3.5±0.2)×10^{-4} cm^2/s) and T_{Br}-T_C ((3.3±0.3)×10^{-3} cm^2/s). The systematic trend in diffusivity correlates with the increasing atomic number of the incorporated halogen (Cl<Br), consistent with progressively stronger SOC. This observation supports the conclusion that HAE directly promotes CT exciton transport. Second, we examined another donor-acceptor system, FR-T_C (fluorene-1,2,4,5-tetracyanobenzene) and its brominated analogue BrFR-T_C (2-bromocarbazole-1,2,4,5-tetracyanobenzene) cocrystals.[47] In this pair, significant broadening of the TADF intensity profiles is observed only in BrFR-T_C, with a ^{3}CT diffusion coefficient determined to be ~(7.3±0.4)×10^{-4} cm^2/s (Supplementary Fig. 14). These results directly demonstrate the universality of HAE-induced CT transport enhancement, which is irrespective of different heavy atoms and distinct cocrystal systems, indicating that the strategy is not material-specific but broadly applicable."

(3) The following reference has been added in the revision (highlighted with a yellow background on page 24).

47 Yao, W. *et al*. Synthesis and photophysical properties of charge transfer cocrystals based on TCNB and fluorene and its derivatives. *CrystEngComm* **26**, 2155-2165 (2024).

4. In Figure 3, the authors state that "With delay time increasing, the TADF spot of both CT states expands into a discernible elliptic pattern, indicating the anisotropic CT migration in TBr-TC (Fig. 3a and 3b)." However, the elliptic pattern is highly difficult to discern, and these patterns look more like a circle.

Response: We appreciate the reviewer's valuable comment. To address the reviewer's concern, the linear fitting of 1D time-dependent Gaussian variances ($\sigma^2(t)$) along the θ = 0° and θ = 90° directions of both CT_1 and CT_2 states were compared. For both CT_1 and CT_2 states, the Gaussian variances extracted from the θ = 0° direction are all larger than those along the θ = 90° direction at the indicated delay times (Fig. R4). More intuitively, both the normalized TADF

intensity profiles of the CT_1 state (at 4 μs) and the CT_2 state (at 3.5 μs) exhibit a broader distribution along the θ = 0° direction compared to that along the θ = 90° direction (Fig. R4). These results directly validate the anisotropic CT migration of both CT states in T_{Br}-T_C.

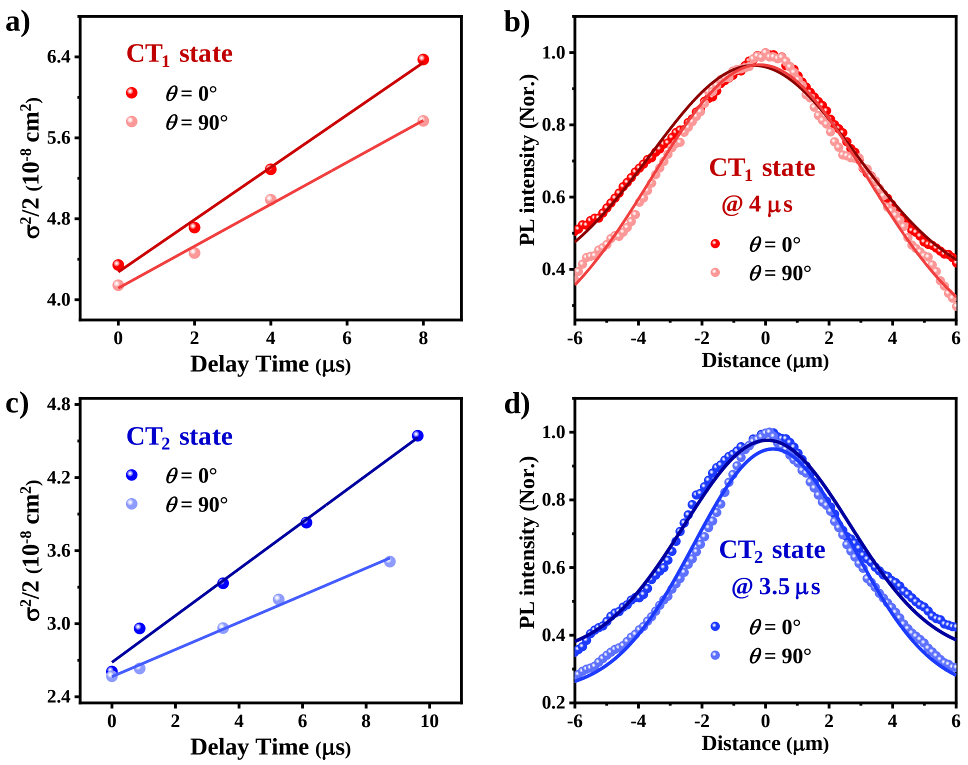

Fig. R4 Comparison of the linear fitting of 1D time-dependent Gaussian variances ($\sigma^2(t)$) along the θ = 0° and θ = 90°directions of (a) CT_1 state and (c) CT_2 state in T_{Br}-T_C. Comparison of normalized 1D TADF intensity profiles of (b) CT_1 and (d) CT_2 excitons in T_{Br}-T_C extracted from θ = 0° θ = 90°directions in Fig. 3**a** and 3**b,** respectively, at indicated delay times.

To enhance the clarity, the elliptic patterns marked in Fig. 3a and 3b have been corrected according to the Gaussian fitting results along the θ = 0° and θ = 90° directions, respectively. These corrections have been highlighted with yellow backgrounds on pages 10-11 of the revised main text.

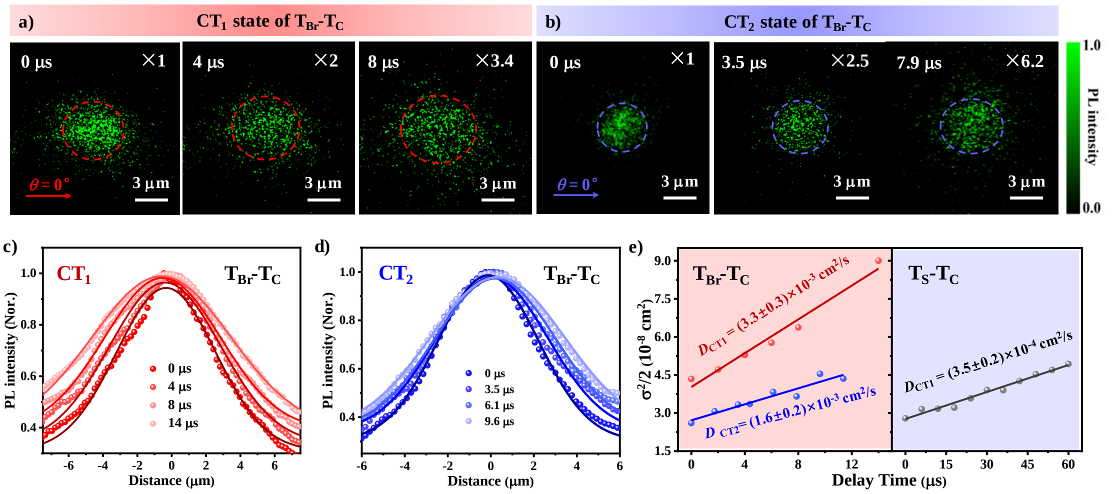

Fig. 3 Imaging and modeling of CT exciton transport. Time-dependent TADF intensity images of (**a**) CT$_1$ and (**b**) CT$_2$ excitons in T_{Br}-T_C collected at 670-750 nm and 490-550 nm, respectively. Scale bars are 3 μm. The dash lines represent the exciton distribution obtained from the Gaussian fittings. Normalized 1D TADF intensity profiles of (**c**) CT$_1$ and (**d**) CT$_2$ excitons in T_{Br}-T_C extracted from the $\theta = 0°$ direction in panel **a** and **b,** respectively, at indicated delay times. The solid lines are their Gaussian fittings. (**e**) The determination of TADF-related exciton diffusion coefficient by the linear fitting of 1D time-dependent Gaussian variances ($\sigma^2(t)$) along the $\theta = 0°$ direction.

5. Some related references might be included in the revised manuscript: 10.1002/anie.202416181; 10.1002/anie.202005933; 10.1002/anie.201712104

Response: Thank for the reviewer's kind suggestion. To address the reviewer's comments, the following changes have been made in the revised manuscript.

(1) The following sentences have been revised and highlighted with yellow background on pages 4-5 of the revised manuscript.

"In this work, organic TADF cocrystals with well-defined crystalline structures and intense TADF emissions were employed as model systems for the exploration of CT exciton transport dynamics.[31-34] Two representative TADF cocrystals were synthesized: one with bromine functionalization (*trans*-4-Bromostilbene-1,2,4,5-tetracyanobenzene, named as T_{Br}-T_C) and one without (*trans*-1,2-diphenylethylene-1,2,4,5-tetracyanobenzene, named as T_S-T_C). By using TADF-scanned imaging microscopy, the influence of HAE on the long-distance CT exciton transport was systematically investigated."

(2) The following references have been added to the revised manuscript and highlighted with yellow backgrounds on pages 22-23.

31 Wang, X. *et al*. Recent Advances of Organic Cocrystals in Emerging Cutting-Edge Properties and Applications. *Angew. Chem. Int. Ed.* **63**, e202416181 (2024).

32 Wang, Z. *et al*. Rational Control of Charge Transfer Excitons Toward High-Contrast Reversible Mechanoresponsive Luminescent Switching. *Angew. Chem. Int. Ed.* **59**, 17580-17586 (2020).

33 Ye, H. Q. *et al.* Molecular-Barrier-Enhanced Aromatic Fluorophores in Cocrystals with Unity Quantum Efficiency. *Angew. Chem. Int. Ed.* **57**, 1928-1932 (2018).

34 Sun, L., Wang, Y., Yang, F., Zhang, X. & Hu, W. Cocrystal engineering: a collaborative strategy toward functional materials. *Adv. Mater.* **31**, 1902328 (2019).

6. There are some spelling and grammar errors in the main text. Please check and correct them. For example, page 4 in "These limitations substantially diminish the actual utilization of CT exctons despite their potential for long-distance migration", "exctons" should be "excitons".

Response: We thank the reviewer for the valuable comments and apologize for any oversight in our manuscript. After carrying out a comprehensive and thorough revision, the spelling error of "exctons" on page 4 and the grammar error "putted" in *Methods—Photocurrent response measurements* section have been corrected to "excitons" and "placed", respectively. Both errors are highlighted with yellow backgrounds on pages 4 and 19.

Reviewer #3:

The manuscript titled "Heavy atom functionalization promotes triplet-assisted charge-transfer exciton transport in organic cocrystals" by Xiao et al. investigated a strategy for enhancing the diffusivity of charge-transfer (CT) excitons in organic

cocrystals. Through the heavy atom effect, the authors observed promoted ISC and RISC kinetics, as well as a long CT migration distance exceeding 16 μm. These compelling results demonstrate that HAE can serve as an effective approach to promote CT transport in organic materials. Overall, the findings of the current manuscript are interesting, the experiments are robust, and the conclusions are well supported by experimental results. I therefore would like to recommend that the paper be accepted if the following minor points could be well addressed.

Response: We are deeply grateful to the reviewer for his/her thorough review and positive feedback.

Q1: In the theoretical calculations of energy levels in TBr-TC, the CT2 and CT1 states are identified. However, the calculated energy difference between these two states is smaller than that observed experimentally, and the PL spectra of TS-TC and TBr-TC appear to be similar, could the authors elucidate the underlying reasons for these discrepancies?

Response: We thank the reviewer for the constructive and insightful comments. To enable a more intuitive comparison of the differences in PL spectra between T_S-T_C and T_{Br}-T_C, the multi-peak fitting was conducted on the PL spectrum of T_{Br}-T_C. As shown in the revised Fig. 2a, more obvious spectral differences between T_S-T_C and T_{Br}-T_C can be observed. Different from the single-peak emission of T_S-T_C centered at ~574 nm, T_{Br}-T_C exhibits two emission peaks centered at ~555 nm and ~585 nm, which can be attributed to emissions from the CT_2 and CT_1 states, respectively. Based on these two values, the energy difference between the CT_2 and CT_1 states ($\Delta E_{CT2\text{-}CT1}$) is estimated to be ~114 meV in T_{Br}-T_C, which is slightly larger than the theoretically calculated value of ~90 meV. To enhance the clarity, Fig. 2a has been revised and highlighted with a yellow background on page 7 of the main text.

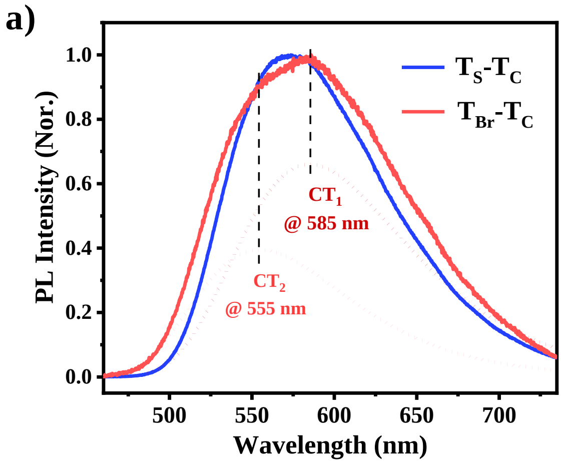

Fig. 2 (a) PL spectra of T_S-T_C and T_{Br}-T_C under the 375 nm excitation. The dash lines are the PL spectra of CT_1 (dark red) and CT_2 (light red) state emissions after multi-peak fitting, which are centered at ~585 nm and ~555 nm, respectively.

A minor discrepancy between the theoretically and experimentally determined energy level differences is commonly observed in numerous reported studies (*Nat. Commun.* 2024, 15, 4674; *Chem. Mater.* 2016, 28, 9088;), and errors in both theoretical calculations and experimental measurements are contributing factors. In terms of theoretical calculation, the molecular geometry obtained from computational optimization may deviate from the actual molecular configuration. As for the experimental factor, the $\Delta E_{CT2\text{-}CT1}$ is calculated based on the difference in peak positions of the CT_2 and CT_1 state emissions. The systematic errors in PL spectra measurements, the broadening of PL emissions caused by molecular vibration, and the mathematical errors in multi-peak fitting are all inevitable factors contributing to the uncertainty of the experimentally determined $\Delta E_{CT2\text{-}CT1}$. Therefore, we think that the minor discrepancy between the theoretical and experimental values of $\Delta E_{CT2\text{-}CT1}$ (90 meV and 114 meV, respectively) is unavoidable and reasonable in our case.

Q2: The authors claim that the Br content directly affects the CT transport dynamics. When the Br content is lower than 50%, is the CT2 state completely absent, or is its signal simply too weak to be detected?

Response: Thanks for the reviewer's valuable comments. It is reasonable to speculate that the CT_2 state should exist as long as the Br atom is incorporated. Although the measured PL spectra and PL kinetics only exhibit obvious time-dependent shifts and wavelength-dependent differences when the Br content exceeds 50% (Supplementary Fig. 20 and 21), the PL kinetics of both prompt fluorescence and TADF are accelerated upon the Br incorporation (Fig. 4c and 4d). Therefore, we believe that the CT_2 state should exist but is too weak to be detected when the Br content is lower than 50%. To address the reviewer's comments, the following sentence has been revised and highlighted with a yellow background on page 14 of the main text:

"As for the $^3CT_2$ state, although it is expected to form upon the Br incorporation, it becomes clearly detectable only when T_{Br} content exceeds ~50% (Supplementary Fig. 20 and 21)."

Q3: Have the authors explored substitution with other heavy atoms (for example, iodine), and if so, does it lead to similar effects on charge-transfer exciton transport?

Response: We sincerely thank for the reviewer's thoughtful suggestions. To further investigate the HAE-promoted CT exciton transport, chlorine and iodine atoms were employed for heavy atom functionalization instead of bromine. The T_{Cl}-T_C (*trans*-4-Chlorostilbene-1,2,4,5-tetracyanobenzene) cocrystal was successfully synthesized (Fig. R2), whereas the iodide donor T_I (*trans*-4-Iodostilbene) failed to form cocrystals with the T_C acceptor. The thermodynamic instability of the hypothetical T_I-T_C cocrystal and the kinetically favored single-component nucleation may account for the unsuccessful cocrystallization.

We next compared the CT exciton transport properties among the T_S-T_C, T_{Cl}-T_C and T_{Br}-T_C cocrystals. As shown in Fig. R2, T_{Cl}-T_C exhibits a notable ^3CT exciton transport. The diffusion coefficient was determined to be ~$(1.0\pm0.1)\times10^{-3}$ cm^2/s, which lies between the ^3CT diffusion coefficients of T_S-T_C ($(3.5\pm0.2)\times10^{-4}$ cm^2/s) and T_{Br}-T_C ($(3.3\pm0.3)\times10^{-3}$ cm^2/s). These results not only validate the universality of HAE-promoted CT exciton transport, but also indicate a positive

correlation between the promotion and the relativistic mass of the introduced heavy atoms. To better address the reviewer's comments, following changes have been revised in manuscript.

(1) Fig. R2 (Supplementary Fig. 13) has been added and highlighted with a yellow background on page S13 of the revised SI.

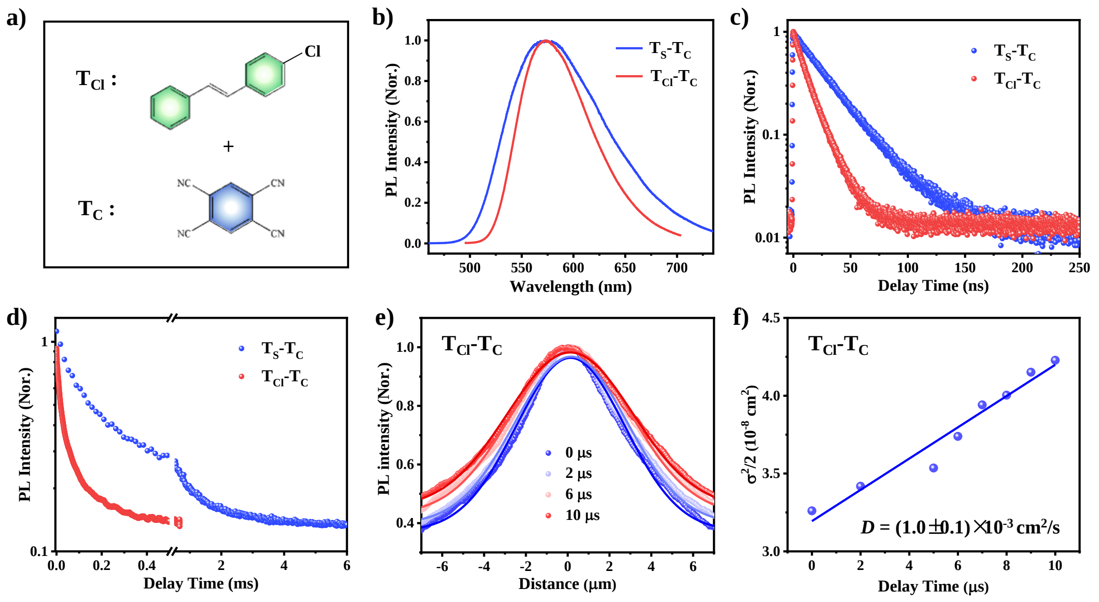

Fig. R2 (Supplementary Fig. 13) Imaging and modeling of CT exciton transport in T_{Cl}-T_C. (a) Chemical structures of T_{Cl} and T_C. Comparison of the normalized (b) PL spectra, (c) PL kinetics on ns timescale, and (d) PL kinetics on ms timescale between T_S-T_C and T_{Cl}-T_C. (e) Normalized 1D TADF intensity profiles of T_{Cl}-T_C at different delay times, along with their Gaussian fittings. (f) The determination of the ^{3}CT diffusion coefficient through linearly fitting of the 1D time-dependent Gaussian variances ($\sigma^2(t)$).

(2) Following sentences have been revised, which are highlighted with yellow backgrounds on pages 11-12 of the main text.

"Furthermore, we also verified the universality of HAE-promoted triplet CT exciton transport across different heavy atoms and material systems. First, the chlorine atom was employed as an alternative heavy atom instead of the bromine atom. The T_{Cl}-T_C (*trans*-4-Chlorostilbene-1,2,4,5-tetracyanobenzene) cocrystal was successfully synthesized and exhibits notable ^{3}CT exciton transport, with a diffusion coefficient of ~(1.0±0.1)×10^{-3} cm^2/s (Supplementary Fig. 13). This value lies between those of T_S-T_C

(($(3.5\pm0.2)\times10^{-4}$ cm^2/s) and T_{Br}-T_C (($3.3\pm0.3)\times10^{-3}$ cm^2/s). The systematic trend in diffusivity correlates with the increasing atomic number of the incorporated halogen (Cl<Br), consistent with progressively stronger SOC. This observation supports the conclusion that HAE directly promotes CT exciton transport."

"These results directly demonstrate the universality of HAE-induced CT transport enhancement, which is irrespective of different heavy atoms and distinct cocrystal systems, indicating that the strategy is not material-specific but broadly applicable."

Q4: In the text, the authors attributed the enhancement of photocurrent to the increased migration distance of CT excitons. However, photocurrent can be influenced by many other factors, such as exciton dissociation, charge extraction, electrode contact. It would be helpful if the authors could provide additional experimental or computational evidence to clarify whether enhanced exciton migration is the dominant factor underlying the observed photocurrent improvement.

Response: We thank the reviewer for the insightful comments and constructive suggestions. To address the reviewer's concern, the nature of carrier transport under a 20 V bias voltage was first verified via excitation-intensity dependent PL intensity measurements (Fig. R5). The initial maximum PL intensity (I_0, at delay time $t \approx 0$ ps) as a function of excitation intensity (P) follows the law of $I_0 \propto P^{\alpha}$, where α reflects the existence form of carriers. When carriers exist as excitons, $\alpha = 1$; when all carriers are free charges, $\alpha = 2$; when excitons and free charges coexist, $1 < \alpha < 2$. As shown in Fig. R5, the α values at 0 V and 20 V are both determined to be ~1, indicating the excited CT state in T_{Br}-T_C predominantly exists in the form of excitons even at 20 V bias. Therefore, the exciton dissociation within the cocrystal and the subsequent charge transport process are negligible.

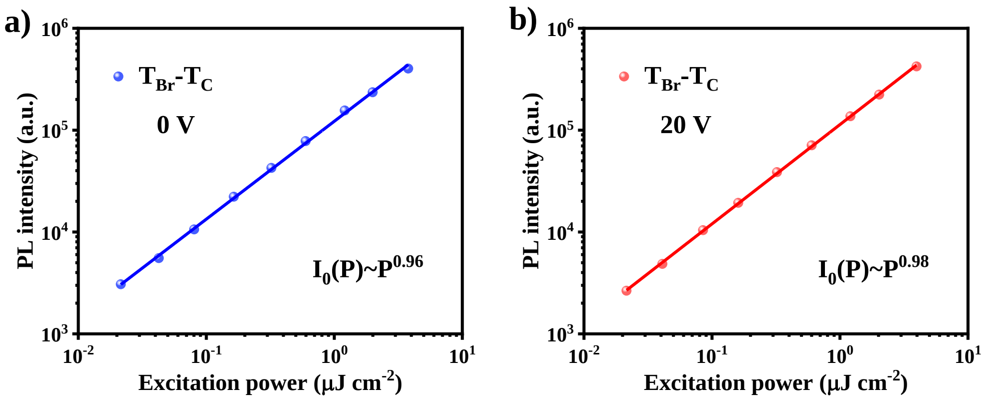

Fig. R5 (Supplementary Fig. 25) Initial maximum PL intensity (I_0, at delay time t ≈ 0 ps) as a function of excitation intensity (P) for T_{Br}-T_C measured at (a) 0 V and (b) 20 V bias voltage. Both plots follow the law of $I_0 \propto P^{\alpha}$ (solid lines), where the α values are determined to be ~1, indicating that CT excitons dominated in T_{Br}-T_C at both 0 V and 20 V.

Secondly, the influence of charge extraction at the electrode interface was evaluated via excitation-intensity dependent photocurrent measurements under a 20 V bias voltage. A clear linear relationship between the excitation intensity and the measured photocurrent intensity was established (Fig. R6), indicating good electrode contact and efficient charge extraction at the electrode interface.

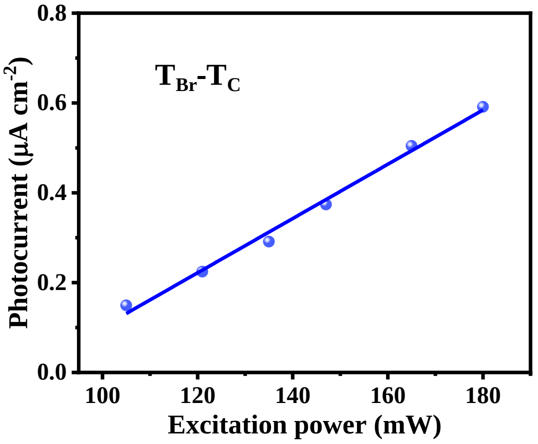

Fig. R6 (Supplementary Fig. 26) Excitation-intensity dependent photocurrent density of T_{Br}-T_C with electrode spacing of 10 μm. A clear linear relationship was established, indicating good electrode contact and efficient charge extraction at the electrode interface.

Thirdly, to further validate the contribution of enhanced CT exciton transport to the improved photocurrent, we measured photocurrent-time (I-t)

curves at varying interelectrode distances. As shown in Fig. R7, the photocurrent density of T_{Br}-T_C remains approximately constant when the electrode spacing (5 μm and 10 μm) is smaller than its average CT migration distance of ~16 μm. However, a noticeable decrease is observed when the spacing (30 μm) exceeds that value. These results directly confirm that the CT exciton transport is decisive for the photocurrent enhancement.

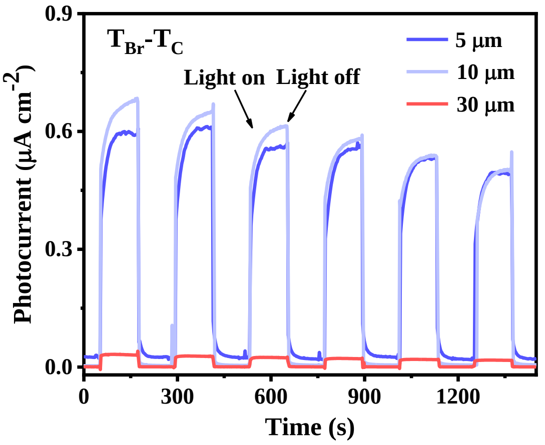

Fig. R7 (Fig. 5b) Time-dependent switching cycles of photocurrent response of T_{Br}-T_C with electrode spacing of 5 μm, 10 μm and 30 μm.

In short, after evaluating all the influence factors of exciton dissociation, charge extraction, and electrode contact, the decisive role of enhanced CT exciton transport in improving photocurrent can be confirmed. To improve our manuscript, following changes have been made in the revision.

(1) Fig. R7 (Fig. 5b) has been added on page 15 of the revised manuscript and highlighted with a yellow background.

(2) Fig. R5 and R6 (Supplementary Fig. 25 and 26) have been added and highlighted with yellow backgrounds on page S22 of the revised SI.

(3) Following sentences have been revised and highlighted with yellow backgrounds on page 14-15 of the revised main text.

"As shown in Fig. 5b, the photocurrent density of T_{Br}-T_C remains approximately constant when the electrode spacing (5 μm and 10 μm) is smaller than its average CT migration distance of ~16 μm. However, a noticeable decrease in photocurrent is

observed when the electrode spacing (30 μm) exceeds this diffusion length. The CT excitons in T_{Br}-T_C remain strongly bound under a bias voltage of 20 V (Supplementary Fig. 25), while efficient charge extraction at the electrode interfaces is facilitated by the favorable contact between cocrystal and electrode (Supplementary Fig. 26). These results directly confirm that the long-distance CT exciton transport is a key factor governing the photocurrent response in CT cocrystals. Furthermore, a significantly enhanced photocurrent response was observed with increasing T_{Br} content (Fig. 5c)."

(4) The methods of photocurrent response measurements have been revised and highlighted with yellow backgrounds on page 19 of the revised manuscript.

Q5: The authors mentioned that structural asymmetry affects charge transport, do the authors consider that the introduction of heavy atoms may modify structural asymmetry, for example by altering crystal packing, molecular orientation, or π-π interactions? If so, could such structural changes also contribute to the observed CT exciton transport behavior?

Response: We sincerely appreciate the reviewer's kind and insightful discussions. We concur with the reviewer that the HAE-induced structural changes substantially contribute to the transport behavior of both CT states in T_{Br}-T_C. In fact, we have considered the contributions of such structural changes, including the distances between D and A molecules, the transport distances between the adjacent D-A units, and the asymmetry of CT exciton transport pathways, to the transport properties of CT excitons, such as diffusivity and anisotropy.

Firstly, based on the single crystal structures of T_S-T_C and T_{Br}-T_C (Fig. 1b and 1c), we find that the D-A distances in T_{Br}-T_C (~3.05 Å and ~3.28 Å for segments with and without Br group, respectively) are both decreased compared to ~3.36 Å observed in T_S-T_C. This serves as direct evidence for the enhanced π-π interaction (CT coupling as well) in T_{Br}-T_C resulting from the heavy atom incorporation. This enhanced intermolecular interaction further modifies the properties of CT excitons in two aspects: (1) the CT_1 emission in T_{Br}-T_C (~585 nm) is slightly red-

shifted relative to that in T_S-T_C (~574 nm), as the enhanced CT coupling may lead to a narrower band gap of the CT_1 state (Fig. 2a); (2) although the adjacent transport distances for both CT states in T_{Br}-T_C (7.90 Å and 7.93 Å) are larger than that of the CT_1 state in T_S-T_C (7.58 Å, Supplementary Fig. 1 and 2), their diffusivities are both increased ((3.3±0.3)×10^{-3} cm^2/s and (1.6±0.2)×10^{-3} cm^2/s in T_{Br}-T_C *vs*. (3.5±0.2)×10^{-4} cm^2/s in T_S-T_C) due to the stronger intermolecular interaction and enhanced molecular orbital overlap in T_{Br}-T_C.

Secondly, the incorporation of Br atoms breaks the spatial symmetry of the A-D-A unit and subsequently results in the crystallization of T_{Br}-T_C in *Cc* space group, which has lower symmetry compared to the triclinic *P-1* space group of T_S-T_C (Fig. 1b and 1c). The altered crystal packing significantly influences the transport pathways of CT excitons (Supplementary Fig. 1 and 2), which directly leads to the anisotropic transport of both CT excitons in T_{Br}-T_C (Fig. 3 and Supplementary Fig. 11).

Therefore, the incorporation of heavy atoms not only contributes to the enhancement of intermolecular interaction (π-π interaction and the overlap of molecular orbitals), but also modifies the structural symmetry. These two factors respectively affect the diffusivity and the anisotropy of CT exciton transport. To enhance the clarity of our manuscript, the following sentences have been revised in the revised manuscript and are highlighted with yellow backgrounds on page 11.

"Although the adjacent transport distances for the two CT states in T_{Br}-T_C (7.90 Å and 7.93 Å) are larger than that of CT_1 state in T_S-T_C (7.58 Å, Supplementary Fig. 1 and 2), exciton transport in T_{Br}-T_C is nevertheless significantly enhanced. This improvement can be attributed to stronger intermolecular interactions and the enhanced electronic coupling, as reflected by the larger SOC matrix elements in T_{Br}-T_C (Supplementary Table 3). Therefore, these findings indicate that the HAE enhances CT transport not through reduced intermolecular distances, but through strengthened electronic interactions and SOC, thereby providing direct dynamic evidence for HAE-facilitated exciton transport."

Point-by-point response to manuscript NCOMMS-25-91317A

Reviewer #1:

The authors have adequately addressed my concerns. I recommend this work for publication.

Response: We sincerely appreciate the reviewer's decision to accept our paper.

Reviewer #2:

it can be accepted now

Response: We sincerely appreciate the reviewer's decision to accept our paper.

Reviewer #3:

I think the authors have made quite some efforts to revise the manuscript.

Response: We are grateful to the reviewer for the positive feedback and valuable suggestions. We have carefully revised our manuscript and hope that it now aligns with the journal's standards.

(1) When looking at other reviewers' comments (Q4 of Reviewer 3), I feel the section trying to use photocurrent to justify the exciton transport requires significant addition justifications (I doubt PL measurement gives direct evidence that the exciton are not dissociated and are responsible for the photocurrent. Also the attenuation of photocurrent as a function of channel length requires a much more detailed investigation for the claim the authors intend to make) and would like suggest the authors to remove the section.

Response: We thank the reviewer for the valuable suggestions and sincerely apologize for the unclear description and explanation in the previous manuscript.

Firstly, for the PL measurements, the power-law dependence of PL intensity on excitation intensity ($I_0 \propto P^{\alpha}$) reflects the order of the recombination process. Excitonic radiative recombination is a first-order process yielding $\alpha \approx 1$, while bimolecular recombination of free electrons and holes is a second-order process,

resulting in α ≈ 2. This power-law dependence of PL intensity is a well-established method which has been widely used to distinguish whether the excited charges exist in the form of excitons or free carriers (*J. Am. Chem. Soc.*, 2021, 143, 19128; *J. Am. Chem. Soc.*, 2019, 141, 15557; *Phys. Chem. Chem. Phys.*, 2017, 19, 9143). In our measurements (Supplementary Fig. 25), the α values at 0 V and 20 V are both determined to be ~1, indicating that the excited CT excitons are not dissociated even at 20 V bias.

Secondly, to better clarify the relationship between photocurrent and electrode spacing, we can estimate the drift distance of free carriers (assuming that the excited charges exist as free carriers) using:

$$L_{drift} = \mu E \tau = \mu V \tau / d$$

where μ is the carrier mobility, E the applied electric field, τ the carrier lifetime, V the applied voltage, and d the electrode spacing. Using $\mu \approx 0.13$ cm^2/V·s (derived from the diffusion coefficient of 3.3×10^{-3} cm^2/s), $E \approx 3.7\times10^5$ V/m, and $\tau \approx 223.6$ µs, the drift distance at the maximum electrode spacing of 30 µm was estimated to be ~1075 µm. This value is much larger than the electrode spacing, indicating that variations in spacing do not limit free carrier transport, and the photocurrent density should remain essentially constant if free carriers dominate the transport. However, as shown in Fig. 5b, the photocurrent density of T_{Br}-T_C remains nearly constant for electrode spacings (5 and 10 µm) smaller than the CT exciton migration distance (~16 µm), but decreases significantly when the spacing (30 µm) exceeds that distance. These results directly confirm that the observed photocurrent behavior is primarily determined by the transport of CT excitons rather than free carriers.

Based on these additional analyses and clarifications, we believe that the photocurrent results provide useful evidence for the potential of long-distance CT transport to improve the performance of optoelectronic devices. Therefore, rather than removing this section, we have revised the relevant discussion in the manuscript. The following changes have been made in the revision.

(1) Following sentences have been revised and highlighted with yellow backgrounds on page 12 of the revised main text.

"The CT excitons in T_{Br}-T_C remain strongly bound even under a bias voltage of 20 V, as evidenced by the power-law dependence of PL intensity on excitation intensity (Supplementary Fig. 25).[50,51] Efficient charge extraction at the electrode interfaces is also ensured by the favorable contact between the cocrystal and electrodes (Supplementary Fig. 26). These results directly confirm that the long-distance CT exciton transport, rather than the drift of free carriers, is the key factor governing the photocurrent response in CT cocrystals."

(2) The following references have been added to the revised manuscript and highlighted with yellow backgrounds on page 21.

50 Sun, Q. *et al.* Ultrafast and high-yield polaronic exciton dissociation in two-dimensional perovskites, *J. Am. Chem. Soc.* **143**, 19128-19136 (2021).

51 Zhang, Z., Fang, W.-H., Long, R. & Prezhdo, O. V. Exciton dissociation and suppressed charge recombination at 2D perovskite edges: key roles of unsaturated halide bonds and thermal disorder. *J. Am. Chem. Soc.* **141**, 15557-15566 (2019).

(2) The authors should change the title to better connect with the fundamental physics/chemistry that enables the better transport.

Response: We thank the reviewer for the valuable suggestion regarding the title. After careful consideration, we believe that the current title accurately reflects the main focus and key findings of this work, particularly the emphasis on heavy-atom functionalization and triplet-assisted CT exciton transport. To improve the clarity and academic presentation, we have made a minor revision by introducing a hyphen in the title.

"Heavy-atom functionalization promotes triplet-assisted charge-transfer exciton transport in organic cocrystals"
